# Analysis of whole-genome re-sequencing data of ducks reveals a diverse demographic history and extensive gene flow between Southeast/South Asian and Chinese populations

**DOI:** 10.1186/s12711-021-00627-0

**Published:** 2021-04-13

**Authors:** Fan Jiang, Ruiyi Lin, Changyi Xiao, Tanghui Xie, Yaoxin Jiang, Jianhai Chen, Pan Ni, Wing-Kin Sung, Jianlin Han, Xiaoyong Du, Shijun Li

**Affiliations:** 1 Hubei Key Laboratory of Agricultural Bioinformatics, College of Informatics, Huazhong Agricultural University, Wuhan, 430070 People’s Republic of China; 2 Key Lab of Agricultural Animal Genetics, Breeding and Reproduction, Ministry of Education, College of Animal Science and Technology, Huazhong Agricultural University, Wuhan, 430070 People’s Republic of China; 3 College of Animal Science, Fujian Agriculture and Forestry University, Fuzhou, 350002 People’s Republic of China; 4 Institute for Systems Genetics, West China Hospital, Auspiciousness Peace Center, Gaopeng Avenue, Wuhou District, Chengdu, 610041 People’s Republic of China; 5 Department of Computer Science, National University of Singapore, Singapore, 117417 Singapore; 6 International Livestock Research Institute (ILRI), Nairobi, Kenya; 7 CAAS-ILRI Joint Laboratory on Livestock and Forage Genetic Resources, Institute of Animal Science, Chinese Academy of Agricultural Sciences (CAAS), Beijing, People’s Republic of China; 8 Joint International Research Laboratory of Modern Agricultural Technology, Ministry of Education, Jilin Agricultural University, Changchun, 130118 People’s Republic of China

## Abstract

**Background:**

The most prolific duck genetic resource in the world is located in Southeast/South Asia but little is known about the domestication and complex histories of these duck populations.

**Results:**

Based on whole-genome resequencing data of 78 ducks (*Anas platyrhynchos*) and 31 published whole-genome duck sequences, we detected three geographic distinct genetic groups, including local Chinese, wild, and local Southeast/South Asian populations. We inferred the demographic history of these duck populations with different geographical distributions and found that the Chinese and Southeast/South Asian ducks shared similar demographic features. The Chinese domestic ducks experienced the strongest population bottleneck caused by domestication and the last glacial maximum (LGM) period, whereas the Chinese wild ducks experienced a relatively weak bottleneck caused by domestication only. Furthermore, the bottleneck was more severe in the local Southeast/South Asian populations than in the local Chinese populations, which resulted in a smaller effective population size for the former (7100–11,900). We show that extensive gene flow has occurred between the Southeast/South Asian and Chinese populations, and between the Southeast Asian and South Asian populations. Prolonged gene flow was detected between the Guangxi population from China and its neighboring Southeast/South Asian populations. In addition, based on multiple statistical approaches, we identified a genomic region that included three genes (*PNPLA8*, *THAP5*, and *DNAJB9*) on duck chromosome 1 with a high probability of gene flow between the Guangxi and Southeast/South Asian populations. Finally, we detected strong signatures of selection in genes that are involved in signaling pathways of the nervous system development (e.g., *ADCYAP1R1* and *PDC*) and in genes that are associated with morphological traits such as cell growth (e.g., *IGF1R*).

**Conclusions:**

Our findings provide valuable information for a better understanding of the domestication and demographic history of the duck, and of the gene flow between local duck populations from Southeast/South Asia and China.

**Supplementary Information:**

The online version contains supplementary material available at 10.1186/s12711-021-00627-0.

## Background

Thanks to their high reproductive capacity, the duck (*Anas platyrhynchos*) industry achieves great economic benefits from meat, eggs, and feathers [1]. Ducks were among the earliest domesticated fowls in the world [2], which began at least 2000 years ago in China [3, 4]. Today’s domesticated ducks descend mainly from the wild mallard and from the spot-billed duck [5]. Based on whole-genome data and using diffusion approximation to the allele frequency spectrum [6], divergence between the Chinese domesticated ducks and the wild ducks has been estimated to have occurred ~ 2200 generations ago.

Domestication is considered a long-term diffusion process and involves gene flow between wild and domesticated populations, which can occur during or after domestication [7, 8]. Extensive gene flow has been reported between different domestic breeds [9] and between domestic animals and their wild counterparts during animal domestication [10]. Based on Admixture [11] and D-statistics [12, 13] analyses, Zhou et al. [14] found that limited gene flow occurred between the highly selected Pekin and Gaoyou ducks after domestication. In addition, using demographic modeling [15], Zhang et al. [6] found relatively high gene flow estimates between Chinese wild and domestic ducks, i.e. 1.12 and 3.92 migrants per generation from the meat and egg/dual-purpose breeds, respectively, into the wild duck.

Unlike their wild ancestors, domestic ducks have been subjected to strong human-driven selection, which have led to remarkable phenotypic changes in several important behavioral, physiological and morphological traits [16–19]. Previous analyses of whole-genome selective sweeps identified several genes related to developing brain and nervous system (e.g. *PDC*, *GRIK2* and *GRIN3A*) [6, 14, 20], feather coloring (e.g. *MITF*) [21–24] and meat production (e.g. *IGF2BP1* and *IGF2R*) [14, 25] during duck domestication. Investigating the genetic mechanisms that explain how genetic variation affects phenotypic diversity has recently emerged as a topic of great interest and is important to understand the genetic changes that underlie duck domestication. However, little is known about the domestication mechanism in the Southeast/South Asian duck populations.

The purpose of our study was to use whole-genome sequences to provide insights into the demographic history, gene flow, and domestication of Southeast/South Asian and Chinese duck populations. Such analyses are also important to assess the impact of human selection on these populations and to improve future conservation strategies.

## Methods

### Samples and sequencing

**Fig. 1:**
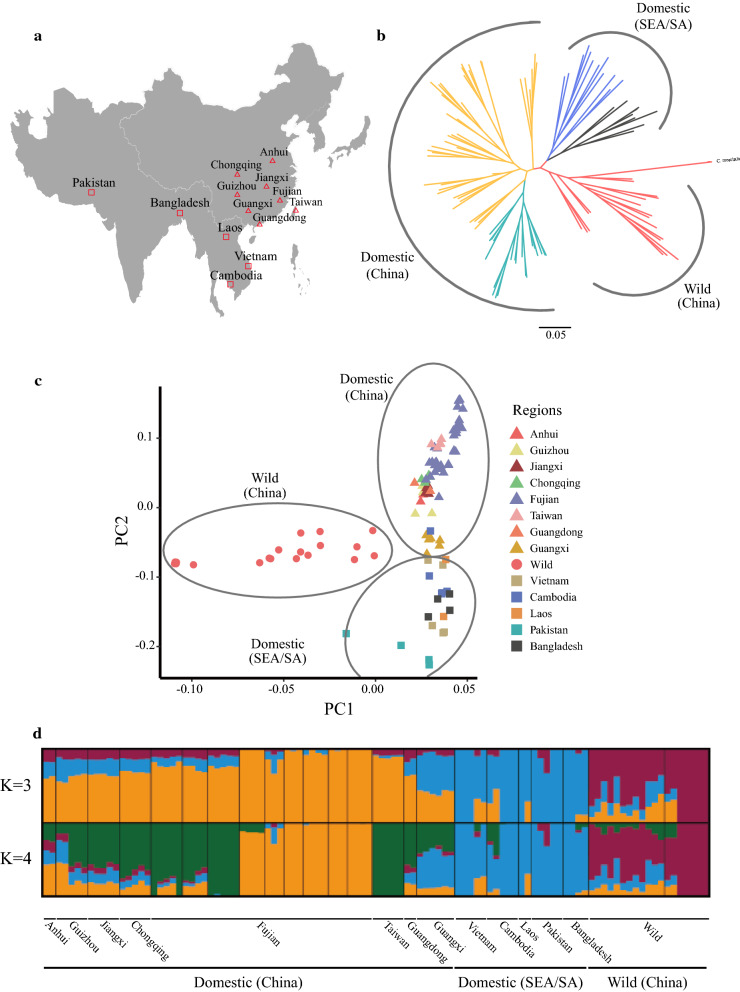
Population genetics of the studied duck populations. **a** Geographic location of the studied ducks (sampling map is drawn using the ggmap package in R). The symbols used for each population correspond to those in (**d**). Additional details are in Additional file 1: Table S1. **b** Neighbor-joining phylogenetic tree constructed using whole-genome SNP data. SEA/SA is an abbreviation for Southeast/South Asian, where blue and grey are Southeast Asian and South Asian populations, respectively. Cyan-blue represents LC × BY duck lineages from China. *Cairna moschata* is the outgroup. **c** Principal component (PC) plots using the first two principal components. The fraction of the variance explained is 8.66% for PC1 and 4.15% for PC2. **d** Genetic structure of the populations. The length of each colored segment represents the proportion of the individual’s genome from K = 3 to 4 ancestral populations. The geographic locations are at the bottom of the figure

In this study, we sequenced the genomes of 78 ducks, including 44 domestic ducks from eight breeds, nine wild ducks from two breeds, four indigenous muscovy (*Cairna moschata*) ducks from southern China, 12 indigenous breeding ducks from Southeast Asia (5 from Vietnam, 5 from Cambodia, and 2 from Laos), nine indigenous breeding ducks from South Asia (5 from Pakistan and 4 from Bangladesh) (Fig. 1a) and (see Additional file 1: Table S1). Animal samples were collected according to local ethical standards. Blood was sampled from the flippers using a standard procedure for venous blood collection. Ducks were kept alive after blood collection. Genomic DNA was extracted from the blood samples and sequenced on an Illumina HiSeq 2500 platform. Using a whole-genome shotgun strategy, we generated 1761.6 Gb of paired-end reads 150 bp long, and a sequence coverage of at least 5× was obtained for each individual (see Additional file 1: Table S1). In addition, to ensure reliability of the results for each population group, we downloaded the resequencing data of 21 Chinese indigenous ducks and 10 Chinese wild ducks from the NCBI database (SRP144280) (see Additional file 1: Table S1).

### Read mapping and variant calling

Raw reads were processed using the Trimmomatic (v-0.36) software [26] to remove adapter and low-quality sequences and then aligned to the *Anas platyrhynchos* genome (IASCAAS_PekingDuck_PBH1.5) using the BWA-MEM (v-0.7.15) software [27] with default parameters, except for the “-t -M -R” option. Alignment bam files were sorted using the Samtools (v-1.3.1) software package [28] and duplicated reads were removed using the Picard tools MarkDuplicates (v-1.108) software [29]. For global realignment of reads around insertions or deletions (indels) before variant calling, we used the RealignerTargetCreator and IndelRealigner tools in the Genome Analysis Toolkit (GATK,v-3.5) [30]. We used the HaplotypeCaller algorithm of the GATK pipeline in the genomic Variant Call Format (gVCF) mode to obtain the genotype likelihoods at each site in the reference genome for each individual. Single nucleotide polymorphisms (SNPs) and indels were simultaneously called using the HaplotypeCaller algorithm, which combines a local re-assembly of haplotypes with a more advanced hidden Markov model (HMM) likelihood function. Then, we merged individual gVCF files into a multi-individual VCF file using the Genotype GVCFs tool of the GATK pipeline. Filtering raw SNP candidates is an essential step in the genotyping workflow because it allows the shrinking of false positive calls due to biases in the sequencing data. Next, these variants were used as input for hard filtering in the GATK pipeline based on seven statistics to identify SNPs or indels: QUAL > 30.0, QD > 2.0, FS < 60.0, MQ > 40.0, MQRankSum > − 12.5, ReadPosRankSum > − 8.0, SOR > 10.0 (indels: QUAL > 30.0, QD > 2.0, FS < 200.0, ReadPosRankSum > − 20.0, SOR > 10.0). In addition, -clusterWindowSize was set to 10 and -clusterSize was set to 3, which means that the number of variants within a 10-bp window should not exceed 3. Generally, such consecutive SNPs can be considered as false positives and removed [31].

### Phylogenetic and population genetic analyses

For the phylogenetic and population genetic analyses, we used the PLINK (v-1.9) [32] software with parameters (-geno 0.05 -maf 0.05) to filter the SNPs selected above. In total, we discovered 4,054,630 high-quality SNPs (excluding the scaffolds that are not on autosomes) among the 109 individuals. This probably represents the most comprehensive catalog of genetic variants in ducks.

Genetic structure was inferred using the Admixture (v-1.3.0) software [11], which uses a maximum-likelihood based method to estimate individual ancestries from multilocus SNP genotype datasets. In order to explore divergence between populations, we set the pre-defined genetic clusters (K) with a range from 2 to 9 to cover the maximum number of lineages. To automate the process of analyzing the results of genotype clustering, we used the CLUMPAK (v-1.1) software [33] and obtained the optimal alignment of ancestry proportions.

Next, we constructed a phylogenetic tree based on the high-quality SNPs using the SNPhylo (v-20140701) software [34], which uses a maximum likelihood method for inference of phylogeny. One thousand bootstraps were used to assess branch reliability. The GCTA (v-1.91.6) [35] software was used for principal component analysis of genotypes at biallelic SNPs and to obtain the eigenvectors from the resulting covariance matrix.

### Inference of the historical pattern of effective population size

To better account for the distribution of variants across the genome of the different duck populations, we used the SMC++ (v-1.15.2) software [36] to estimate the divergence time and effective population size (Ne) without phasing. Compared to previous linkage disequilibrium (LD)-based approaches, this software allows the inclusion of more individuals and generates more accurate estimates of Ne for recent demographic history [36]. The input data from the VCF file were converted into SMC++ input format using the command “smc++ vcf2smc” with default parameters. Then, the historical population sizes were estimated with the command “smc++ estimate (-spline cubic)”. To obtain more reliable estimates of Ne, we performed 20 independent replicate analyses. In the demographic analyses, generation estimates were inferred by assuming that the average mutation rate was 1.91e−9 per base per generation and a generation interval of 1 year (based on an estimate from the chicken genome) [37]. Then, we estimated the time since divergence between the wild and domestic populations using the command “smc++ split” with default parameters. To ensure consistency of the samples, we selected only LT (mallard) wild ducks as test data. Furthermore, in order to more realistically and accurately simulate the dynamic changes of the actual demographic scenarios, we designed different simulation models based on the real data. Then, we used a standard ABC-GLM general linear model approach as implemented in the ABCtoolbox (v-2.0) [38] to evaluate the models (details in Appendix).

### Detecting gene-flow between populations using unlinked SNPs

Treemix (v.1.13) [39] analysis was performed to test the different admixture models for the Southeast/South Asian, Chinese indigenous, and the wild populations using unlinked SNPs. This method applies the covariance matrix of allele frequencies between populations to build a maximum likelihood graph that relates populations to their common ancestor, while taking admixture events (“migration”) into account to improve the fit of the inferred tree. To speed up the analysis, we iteratively used the previous graph with m − 1 migrations as the starting graph and added one migration edge, for a total of m migrations. We rooted the graphs with *C. moschata* (as outgroup) and applied the “-se” option to estimate standard errors of the migration proportions in blocks of 500 SNPs. Migration edges were added until 99.8% of the variance in ancestry between populations was explained by the model [40]. Three-population tests (*f*3 (A; B, C)) implemented in TreeMix were used to further examine evidence of admixture. We obtained significant negative *f*3-statistic values, which indicated that a target population A was admixed between two source populations B and C.

### Analysis of admixture using the D statistic

We used the D-statistic [12, 13] to test for introgression between the Chinese populations and the Southeast Asian or South Asian populations. Four populations (((P1, P2), P3) O), i.e. two ingroups, P1 and P2, a candidate introgressor, P3, and an out-group, O, were compared. In the absence of gene flow, the number of sites at which populations P2 and P3 share the same allele (*ABBA* pattern, where *A* and *B* are the ancestral and derived alleles, respectively) is expected to generate an equal number of sites at which populations P1 and P3 share the same allele (*BABA* pattern), resulting in an expected D-statistic of zero. However, a negative value of the D-statistic means that the potential recipient P1 is closer to the potential introgressor P3 than P2 is. A positive value of the D-statistic means that P2 is closer to P3 than P1 is. In our study, population P1 corresponded to the LC (Liancheng) samples, which had a relatively clean pedigree compared to other Chinese indigenous breeds (Fig. 1d), populations P2 corresponded to the Chinese indigenous populations, P3 corresponded to the Southeast/South Asian samples, and the outgroup (O) corresponded to the *C. moschata* population. For each target population (P2), we performed the D-statistics test against the Southeast/South Asian populations. Then, we evaluated the significance of each test and performed block jack-knifing with a non-overlapping block size of 2 Mb [12, 13]. The weighted block jackknife method tests whether admixture signals are uniform across the genome, and absolute Z-scores higher than 3 were considered as strong evidence of admixture. This analysis was implemented using a Perl script, which is available from github.com/owensgl/abba_baba.

We scanned the locations of gene flow between the Guangxi and Southeast/South Asian populations based on a modified *fd* value [41], using a 100-kb sliding window approach across the genome, with steps of 20 kb. Each window had to contain a minimum of 100 SNPs. The strongest candidate regions of gene flow (i.e. with the highest *fd* values) were visually assessed to determine the amount of gene flow. Then, we verified the top introgressed genomic region based on mean pairwise sequence divergence (*d*_*xy*_) and F_ST_ statistics between the Guangxi and Southeast/South Asian ducks. We also estimated the local ancestry of the top introgressed genomic region along the genomes of the recipients of Guangxi genetic material (Chinese indigenous and Southeast/South Asian groups as donors) by using the ChromoPainter v2 haplotype-based algorithm [42].

### Population admixture analysis using phased SNPs

#### Phasing

Before phasing of SNP genotypes, the data were pruned using the PLINK software to remove triallelic SNPs, sites with a rate of missing data higher than 0.05, and SNPs with a minor allele frequency lower than 0.05. Then, the genotype data were phased jointly for all individuals with the Shapeit2 software [43], using default options.

#### Coancestry matrix inference

To infer population admixture using the phased data, we used the ChromoPainter v2 algorithm [42] based on Li and Stephen’s [44] copying model. Initially, we estimated the switch rate (Ne) and the mutation rate by running Chromopainter (-a mode) on each individual and chromosome, using 10 steps of the expectation maximization (EM) algorithm (-i 10 -in -iM). Then, we averaged the inferred values of each parameter across chromosomes, weighting the average by the number of SNPs, and then averaged them across individuals. Finally, ChromoPainter was run for all chromosomes using the fixed global mutation (0.0135) and switch (306.197) rates, and the resulting co-ancestry matrices (count matrix and length matrix) were summed across chromosomes.

#### Sample clustering and estimation of admixture proportions

First, we used ChromoCombine to estimate the FineStructure C parameter. Then, to identify population structure, we used the FineStructure (v.4.0.1) algorithm [42] by performing 100,000 iterations of the Markov chain Monte Carlo (MCMC) method, sampling from the posterior distribution at every 1000 iterations following 100,000 ‘burn-in’ iterations (− x 100,000 − y 100,000 − z 1000). The FineStructure tree was then inferred using default options (− m T).

We applied the Globetrotter method [45] to assess the ancestral make-up of the Southeast/South Asian populations in terms of ancestral contributions from the Chinese populations. This approach uses the copy vectors from the ChromoPainter that represent the average chunk length that was copied to estimate the proportion of donor ancestry. In brief, we modeled the genome of each Southeast/South Asian breed as a linear mixture of Chinese donor populations using the method described in Leslie et al. [46].

### Linkage-disequilibrium analysis

To estimate the level of LD in each population, the squares of correlation coefficients (r^2^) between alleles at pairs of SNPs were calculated using the PopLDdecay (v-3.40) program [47], with default parameters. The average r^2^ was calculated for each length of distance between SNPs, and the patterns of LD decay were drawn using an R script for the three groups of duck populations.

### Genome scan of divergent regions

We compared the genetic diversity among 44 domestic ducks (25 domestic and 19 highly selected duck), which were considered as one group, to that of 19 wild ducks, and used 40-kb sliding windows across the genome, with steps of 10 kb to detect signatures of selection. The polymorphism levels (θπ, pairwise nucleotide variation as a measure of variability), population fixation statistics (F_ST_), and selection statistics (Tajima’s D, a measure of selection in the genome) were calculated using the VCFtools software (v-0.1.15) [48]. To detect regions with significant signatures of selection, we considered the distribution of the θπ ratios (θπ, wild/θπ, domestic) and of the F_ST_ values. Then, we Z-transformed the distribution of F_ST_ and calculated the log value of the θπ ratios. The significant high θπ ratios [corresponding to the top 5% level, where the log2(θπ) ratio was 0.69] and the F_ST_ values [the top 5% level where Z(F_ST_) was 1.86] were identified as regions with strong signatures of selection along the genome.

Genes located within the 40-kb regions with significant signatures of selection were annotated based on the *Anas platyrhynchos* reference genome. In total, 1049 genes were identified and submitted to the KEGG databases for enrichment analyses.

## Results

### Analysis of population structure

One hundred and nine ducks (*Anas platyrhynchos*), representing eight geographically diverse domestic populations/breeds, two wild populations, and two Muscovy duck populations in China, three local populations in Southeast Asia, and two local populations in South Asia were selected for genome re-sequencing analysis (Fig. 1a) and (see Additional file 1: Table S1). After quality control, we obtained a set of 3,902,414 high-quality SNPs, which were used to construct genetic relationships using a neighbor-joining maximum likelihood method and principal component analysis (PCA). Both methods revealed that these populations from different geographic regions clustered into three major genetic groups, i.e. local Chinese populations, wild populations, and Southeast/South Asian populations (Fig. 1b, c). In addition, it was clear that the Southeast Asian and South Asian populations were sister groups, as were the LC × BY duck (Liancheng white duck × White Kaiya duck) lineages from Fujian and the other Chinese domestic populations (Fig. 1b). It is worth noting that principal component 2 divided the Guangxi population into the Chinese domestic and Southeast/South Asian populations. Furthermore, the F_ST_ values between the Guangxi and the Southeast/South Asian populations (0.024–0.115) were close to those of the Guangxi and Chinese indigenous populations (0.052–0.135) (see Additional file 2: Figure S1).

Admixture analysis shows that the Chinese domestic populations, the Southeast/South Asian populations, and the Chinese wild populations separate into three distinct clusters (K = 3) (Fig. 1d) and (see Additional file 3: Figure S2). When the number of clusters (K) was set to 4, it was evident that individuals (LC × BY duck lineages) from Fujian with direct lineages were separated from the other Chinese domestic ducks. For K ranging from 5 to 9, the Taiwan individuals from the neighboring island of the Fujian area displayed a single ancestry, which differed from that of the Fujian and Southeast/South Asian populations (see Additional file 3: Figure S2). This result is consistent with the higher pairwise F_ST_ values of the Southeast/South Asian populations and Chinese populations (see Additional file 2: Figure S1). Up to K = 7, the Southeast Asian and South Asian populations were clearly separated (see Additional file 3: Figure S2).

### Demographic history

**Fig. 2:**
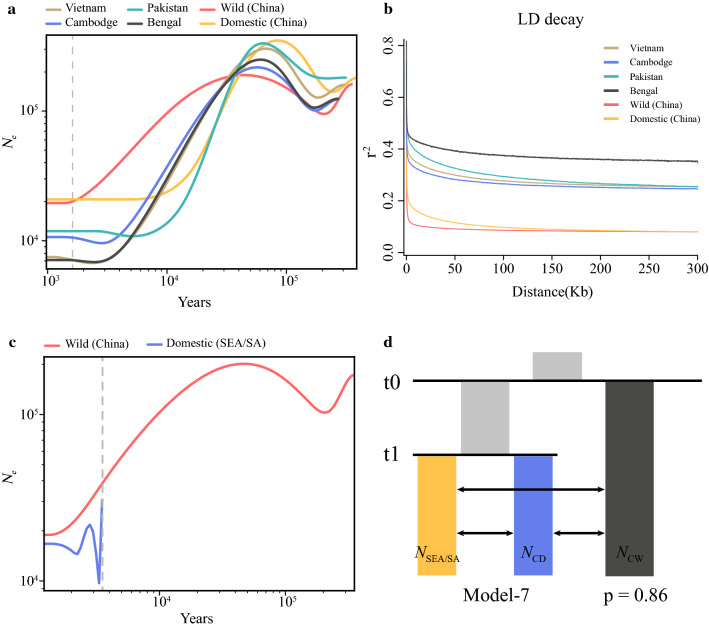
Demographic history of the studied duck populations. **a** Reconstruction of past variations in effective population sizes (Ne) inferred by SMC++. Dashed vertical lines correspond to the estimated splitting times between Chinese domestic ducks and Chinese wild ducks. We assume a mutation rate of 1.91e−9/bp per generation and a generation time of 1 year. **b** Linkage disequilibrium (LD) decay for six populations measured by *r*^2^. **c** Split time between Chinese wild ducks and Southeast/South Asian ducks. **d** The best model for our data. The ancestral population is in light grey, Chinese wild ducks in dark gray, Chinese domestic ducks in blue and SEA/SA ducks in yellow. T0 and T1 indicate the time of divergence between populations. The figures on the arrows indicate the gene flow between populations. The posterior probability value (p) was calculated by the ABCtoolbox

To better understand the evolutionary history of the duck populations with different geographical distributions, we inferred the size of the ancestral populations using the sequential Markov coalescent method implemented in the SMC++ software [36]. The single population approach revealed that the wild duck population experienced a significant expansion of its Ne (Ne ≈ 95,000 up to ≈ 189,000) (Fig. 2a) around 44 to 201 Kya and a subsequent severe Ne contraction (down to ≈ 94,000) around 10 to 44 Kya, which could have contributed to the long decline in Ne during the LGM period [49]. However, the Chinese domestic duck population experienced a stronger bottleneck than the Chinese wild duck population, which could be explained by both domestication and LGM. Similar demographic patterns were observed for the Southeast Asian (Vietnam and Cambodia) and South Asian ducks (Pakistan and Bangladesh), which showed a common trend with an expansion and contraction of population size or genetic diversity over time (10–180 Kya). The Southeast/South Asian duck populations experienced a more severe bottleneck than the Chinese populations, which resulted in the smaller effective population size (between ~ 7100 and ~ 11,900) of modern ducks. In addition, the Southeast/South Asian ducks have a higher level of LD between SNPs (Fig. 2b) and a lower nucleotide polymorphism (θπ) than the Chinese populations (see Additional file 4: Table S2), which suggests that the southern part of China was the core center of duck domestication.

The split analyses on the wild and domesticated ducks with 20 independent replicate tests showed that these two populations diverged about 1700 (between 1333 and 2347) years ago (Fig. 2a) and (see Additional file 5: Figure S3). In addition, divergence time between the Chinese wild and the Southeast/South Asian ducks was estimated at about 3500 (range from 2410 to 4146) years ago, with a marked decrease in population size providing evidence of a genetic bottleneck (Fig. 2c) and (see Additional file 5: Figure S3). To test the hypothesis of gene flow between the Chinese and the Southeast/South Asian ducks, we designed eight simulation models based on the inferred phylogenetic relationships. Remarkably, Model 7 had the highest posterior probability value (0.86), which indicates that gene flow was frequent between these two populations (Fig. 2d) and (see Additional file 6: Figure S4), and see Appendix for details.

### Detection of population admixture

**Fig. 3:**
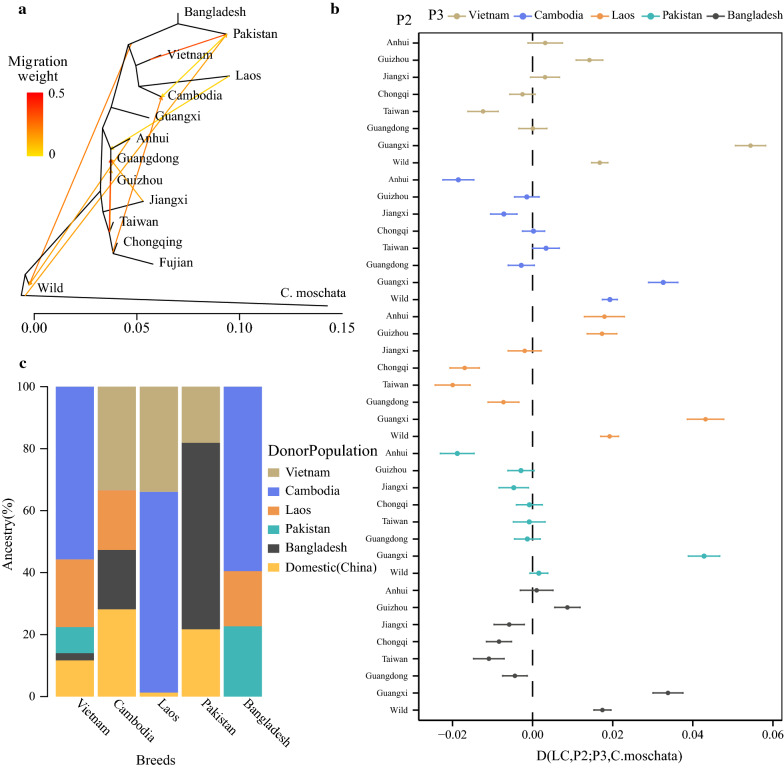
Gene flow between duck populations. **a** Maximum likelihood tree inferred from 15 populations with ten migration edges to detect gene flow. The tree is rooted by the *C. moschata* populations. Populations are colored according to their geographic locations. **b** D-statistics to test for potential gene flow between each Chinese population (P2) and each Southeast/South Asian population (P3). We set P1 as LC (Liancheng) individuals from Fujian with fewer admixture events and O as *Cairna moschata*. We computed the D-statistics (((P1, P2), P3) O). Vertical bars correspond to three standard errors. Positive D values indicate gene flow between P1 and P3, whereas negative D values indicate gene flow between P2 and P3. X and Y axes represent the values of the D-statistic and P2, respectively. **c** Inference of the proportion of ancestral component for each donor population in the genome of each Southeast/South Asian population using Globetrotter

In order to better understand the phylogenetic relationships between the Southeast and South Asian populations and the admixture between the Chinese populations, a phylogenetic tree was constructed using the maximum likelihood algorithm implemented in the Treemix software [39]. Using *C. moschata* as outgroup, the topology of the resulting phylogenetic tree clearly captured the known relationships among the Southeast/South Asian and Chinese duck populations (Fig. 3a) and (see Additional file 7: Figure S5). The tree shows that the indigenous duck populations were domesticated from the wild populations and that the Southeast/South Asian and the Chinese populations are clearly separated, which agrees with results of the analysis of population structure (Fig. 1b–d). The phylogenetic tree also reveals that population structure is mostly correlated with geographic distribution with, for instance, the Guangxi population being located in the middle of the branch of the Chinese domestic and Southeast/South Asian populations. For admixture events, we ran ten independent Treemix analyses. As the number of migration events increased, the proportion of variance in relatedness between populations explained by the model began to asymptote at 0.99 for ten migration edges (see Additional file 8: Figure S6). Across all ten Treemix analyses, gene flow from Chinese indigenous populations occurred mainly in the Jiangxi, Guangdong, Anhui, and Fujian populations (see Additional file 7: Figure S5 and Fig. 3a). In addition, the Chinese wild ducks are shown to have experienced admixture with ducks from Pakistan and Bangladesh. Our results also show that gene flow has occurred from the Laotian ducks to the Guangdong, Guizhou, and Anhui ducks and from the Chongqing and Fujian ducks (selected SM (n = 5) breed) to the Cambodian ducks, with very high migration rates between these populations (0.227), which mirrored a frequent gene flow between the Southeast Asian and Chinese populations. Remarkably, we found that gene flow has occurred at a high migration rate (0.43) between the Vietnamese and Pakistani populations, which indicates frequent gene flow between the Southeast Asian and South Asian populations.

To evaluate admixture, we used the threepop program implemented in the Treemix package [39] to compute *f*3-statistics. Significant negative values of *f*3-statistics (Z-score ≤ − 2) were obtained only when the Guangdong, Guizhou, and wild ducks were the targeted populations (see Additional file 9: Table S3), which indicates that these three populations have experienced admixture with other populations (including the Chinese and Southeast/South Asian populations). This result is consistent with those of the Admixture analysis, i.e. most of the Guangdong, Guizhou, and wild ducks had an ancestral component (see Additional file 3: Figure S2). We also found that these populations had a high rate of heterozygosity (see Additional file 10: Table S4).

### Admixture analysis between the Southeast/South Asian and Chinese domestic populations

We calculated the D-statistic for each individual of the target Guangxi population (P3) (Fig. 3b) and found significant non-zero values that ranged from 0.033 to 0.054 (Z > 3), which indicate that significant gene flow probably occurred between the Southeast/South Asian and the Guangxi populations. Furthermore, we also found a positive D-statistic value between the Anhui and Guizhou populations and the Laotian population (0.0179, 0.0173; Z > 3) (Fig. 3b), which suggests that gene flow occurred between the Laotian and the Chinese indigenous populations and confirms the result of the Treemix analysis (Fig. 3a). To investigate whether gene flow occurred between the Southeast Asian and South Asian populations, we fixed P1 for the Pakistani population, P2 for the Bangladeshi population, P3 for the Southeast Asian populations (Vietnam, Cambodia, and Laos), and O for the *C. moschata* population, which resulted in D-statistic values that confirmed the past occurrence of such gene flow (D = 0.0182–0.0645, Z > 3) (see Additional file 11: Table S5).

### Analysis of population admixture using phased SNPs

Haplotype-based approaches were used to dissect the genetic structure of the duck populations and their genetic relationships. The FineStructure analyses identified three major clusters in the data, comprising the Chinese wild populations, the Chinese domestic populations, and the Southeast Asian and South Asian populations (see Additional file 12: Figure S7). Several sub-clusters within these major geographical clusters indicate a higher level of resolution of the genetic structure. For example, all the Anhui, Guangxi, and two Guizhou ducks clustered into one clade that was positioned close to the branch of the Southeast Asian populations, which is consistent with the Treemix analysis. The Guangxi population also showed significant haplotype sharing with the Southeast/South Asian populations (see Additional file 12: Figure S7).

The Globetrotter analysis revealed that approximately 11.7, 27.7 and 21.7% of the ancestral component in Vietnamese, Cambodian and Pakistani ducks came from a Chinese donor (Fig. 3c), which agrees with results of the Admixture analysis (Fig. 1d). The Chongqing individuals contributed nearly 17% of the Chinese ancestral component as donor to the Cambodian receptor, which is evidence of migration between the Cambodian and Chinese populations (Fig. 3a). Furthermore, the results of the Admixture analysis with K set to 3, also showed that ~ 27.7 to 32.5% (except the pure individuals) and 25.9 to 45.8% of the ancestral component of the Vietnamese and Cambodian ducks came from Chinese domestic ducks. Results based on Globetrotter and Admixture analyses reflect a frequent gene flow between Southeast/South Asian and Chinese populations.

To complement the findings of the D-statistic, Treemix, and f3-statistic analyses, Admixture analysis showed that the Guangxi, Guizhou, and Anhui populations shared, respectively, 35.2, 9.1, and 14.9% of their ancestral component with the Southeast/South Asian populations (see Additional file 13: Figure S8). However, we did not detect the Guangdong population as an ancestor of these populations.

### Genome scan for introgressed regions between the Guangxi and Southeast/South Asian populations

**Fig. 4:**
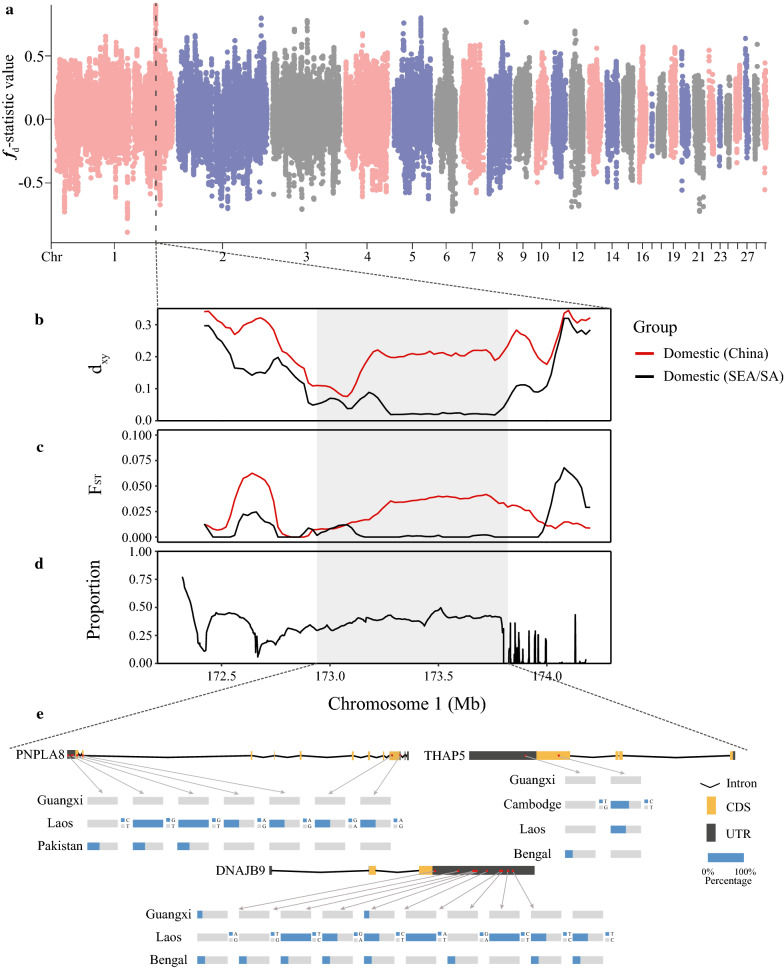
Introgressed regions between the Guangxi and Southeast/South Asian populations. **a** Genome-wide distribution of *fd* values calculated for 100-kb sliding windows with 20-kb steps across the genome to locate the introgressed genomic regions between the Guangxi and Southeast/South Asian populations. Each dot represents a 100-kb window and the dashed line indicates the genomic regions with the strongest introgression signal. **b** Mean pairwise sequence divergence (*d*_*xy*_) of the genomic region with the strongest introgression signal on chromosome 1 between the Guangxi and either the Southeast/South Asian or the Chinese domestic populations. **c** Population differentiation (F_ST_) around the genomic region with the strongest introgression signal on chromosome 1 between the Guangxi and either the Southeast/South Asian or the Chinese domestic populations. **d** Proportion of the Southeast/South Asian ancestral component in the Guangxi population around the genomic region with the strongest introgression signal on chromosome 1 using Chromopainter. **e** Three genes (*PNPLA8*, *THAP5*, and *DNAJB9*) in the genomic region with the strongest introgression signal (chr1: 172,940,001–173,820,000). The blue bars represent the percentage of individuals with mutations in exons

To locate the introgressed genomic regions between the Guangxi and Southeast/South Asian populations, we computed the modified f-statistic (*fd*) and identified 18 genomic regions with extreme *fd* peaks (*fd* $\ge$ 0.5) (see Additional file 14: Table S6). These introgressed regions include 116 genes that are primarily involved in the phosphatidylinositol signaling pathway (e.g., *DGKB, PLCG2,* and *PLCE1*) and glycerophospholipid metabolism (see Additional file 15: Table S7). Treemix analysis of these 18 genomic regions revealed that, compared with the whole-genome phylogenetic tree (Fig. 3a) and (see Additional file 7: Figure S5), the Guangxi population branch is within the cluster of the Southeast/South Asian populations for these regions and that the Guangdong, Guizhou, Anhui populations are genetically close to the Southeast/South Asian populations (see Additional file 16: Figure S9). These results are consistent with the D-statistic and *f*3-statistic analyses. The region with the strongest signal of introgression, which encompasses a 880-kb genomic region (chr1: 172,940,001–173,820,000 bp), showed the highest *fd* values in each Southeast/South Asian population (Fig. 4a) and (see Additional file 17: Figure S10). The mean pairwise sequence divergence (*d*_*xy*_) value for this region was significantly reduced in the Southeast/South Asian ducks but was highly increased in the Chinese indigenous populations (Fig. 4b) and (see Additional file 18: Figure S11). Compared with adjacent non-introgressed genomic regions, the F_ST_ value of the region with the strongest signal of introgression in the Southeast Asia/South Asia ducks is lower than that in the Chinese indigenous ducks (Fig. 4c) and (see Additional file 18: Figure S11). We also used Chromopainter v2, a haplotype-based approach, to ‘paint’ the genomic region with the strongest introgression signal in the Guangxi ducks, using the haplotypes of the Southeast/South Asian and Chinese indigenous populations as donors. The results showed that the proportion of the Southeast/South Asian ancestral component in the Guangxi population was higher in these introgressed regions than in the adjacent non-introgressed genomic regions (Fig. 4d).

We identified three genes (*PNPLA8*, *THAP5*, and *DNAJB9*) in the genomic region with the strongest introgression signal (Fig. 4e) and (see Additional file 14: Table S6). In the Laotian and Pakistani duck genomes, the exons of the *PNPLA8* gene (*patatin like phospholipase domain*) contain nine SNPs, among which one induces a change in the amino acid sequence (e.g., Val253Ala), codon (e.g., CAC > CGC), and the nucleotide sequence (e.g., 173,022,158 A>G). This missense mutation is conserved among birds (see Additional file 19: Figure S12). The *PNPLA8* protein catalyzes the cleavage of fatty acids from phospholipids and regulates the release of lipid second messengers and growth factors. There is also convincing evidence that the *PNPLA8* gene is involved in cell growth in mice [50]. Two exonic SNPs located in the *THAP5* (*thanatos-associated domain containing 5*) gene and 10 exonic SNPs in the *DNAJB9* (*DnaJ heat shock protein family (Hsp40) member B9*) gene, respectively, were detected only in the Laotian and Bangladeshi duck genomes. These genes may, therefore, have a role in the adaptation of ducks to local conditions in Laos and Bangladesh.

### Genome-wide selective sweep

To identify the potential mechanisms that may have been altered through the historical selection of ducks, we measured the genome-wide variations between 19 Chinese wild ducks, 25 Chinese domestic ducks, and 19 Chinese lineage ducks (LC × BY). Compared to wild ducks, the domestic ducks and the lineage ducks had a higher level of LD among loci (P < 2.2e−16, Mann–Whitney U test) (see Additional file 20: Figure S13), which reflects the relatively high degree of inbreeding in domestic populations.

**Fig. 5:**
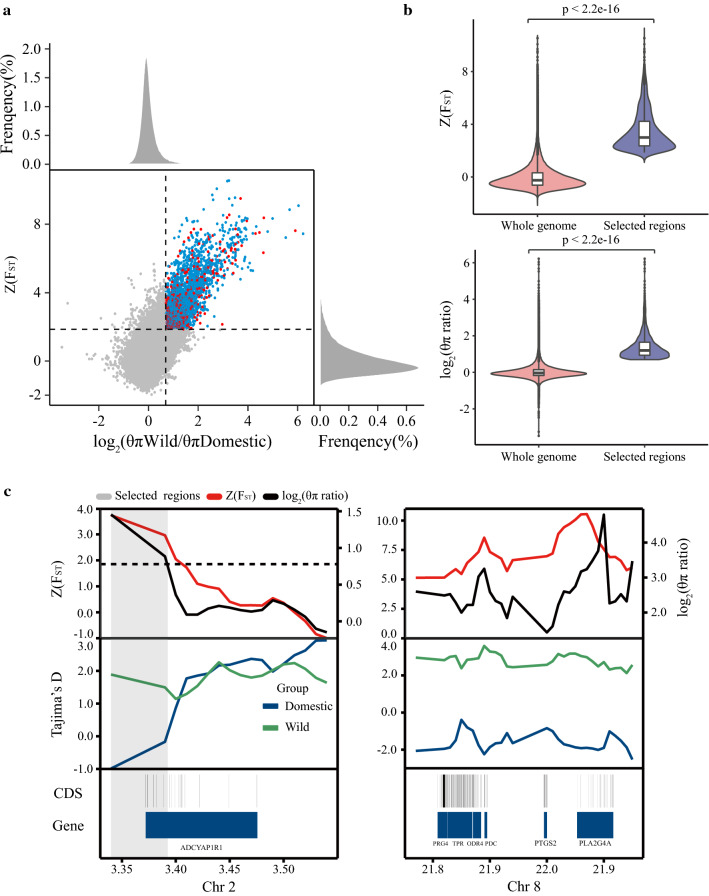
Genomic regions with strong selective sweep signals in wild and domestic duck populations. **a** Distribution of θπ ratios (θπ, Wild / θπ, Domestic) and F_ST_ values, which are calculated for 40-kb sliding windows in 10-kb steps. Data points in blue [corresponding to the top 5% θπ ratio distribution, where the log2 (θπ ratio) is 0.69, and the top 5% F_ST_ distribution, where Z(F_ST_) is 1.86] were identified as selected regions for the domestic ducks. The selected genomic regions contained 1048 genes and are marked in red. **b** Violin plot of F_ST_ values and θπ ratio for duck genomic regions that have undergone strong selection versus the whole genome. The statistical significance was calculated by the Mann–Whitney U test. **c** Example of genes with strong selective sweep signals in domestic ducks. F_ST_ values, θπ ratios and Tajima’s D values are plotted using a 10-kb sliding window. The gray shaded areas were termed as regions with strong selective sweep signals for domestic ducks. Genome annotations are shown at the bottom (black bar, coding sequences (CDS); blue bar, genes)

We selected windows with a significantly high level of polymorphism [θπ ratio (θπ, wild ducks/θπ, domestic)] [5% right tail, where log2(θπ ratio) was 0.69] and high F_ST_ values (5% right tail, where Z(F_ST_) was 1.86) as regions with strong signals of selection along the genome. In total, we identified 23.9 Mb of the domestic duck genome that contain strong signatures of selection, which represent 2.12% of the genome and 1048 genes (Fig. 5a). These regions also exhibited significant differences (P < 2.2e−16, Mann Whitney U test) in Z(F_ST_) and log2(θπ ratio) values compared to the whole genome (Fig. 5b). In addition, we found that the regions with significant differences in selection statistics clustered into two distinct groups (see Additional file 21: Figure S14 and see Additional file 22: Figure S15), i.e. the wild and the domestic ducks. This shows that some portions of the genome have remained distinctly adapted to natural or artificial selection, in spite of the overall similarity along the duck genome.

We identified 1048 genes with both a high F_ST_ and a high θπ-ratio. Several functional categories were highly enriched among these genes, including “Steroid hormone biosynthesis” (6 genes, P = 1.94E−03), “Caffeine metabolism” (3 genes, P = 1.94E−03), “VEGF signaling” (7 genes, P = 7.47E−03) and the FoxO signaling pathway (11 genes, P = 8.43E−03) (see Additional file 23: Table S8). These significantly overrepresented gene ontology terms were mostly related to the term ‘nervous system development’. Among these 1048 genes, we identified 17 genes that are associated with the neuroactive ligand-receptor interaction pathway (see Additional file 23: Table S8), in which a region of the *ADCYAP1R1* gene has undergone a significant selective sweep [Z(F_ST_) > 1.86, log2(θπ ratio) > 0.69] (Fig. 5c). In addition, the *ADCYAP1R1* gene has been reported to play a crucial role in neuronal development during yak domestication [51], which could be an important functional common feature of domestication in these unrelated species.

We also identified two VEGF-related genes (*PTGS2* and *PLA2G4A*) and one RNA transport-related gene (*TPR*) with strong signatures of selection in domestic ducks (Fig. 5c). Interestingly, the *PDC* gene that is adjacent to the VEGF-related genes has undergone a significant selective sweep [Z(F_ST_) = 8.55, log2(θπ ratio) = 3.25)] (Fig. 5c) during domestication. Previous reports have shown that the *PDC* gene has an important functional role in the phenotypic differentiation of wild and domestic ducks by altering the functional regulation of the developing brain and nervous system [6, 14].

In addition to several genes related to the nervous system, we also identified strong signatures of selection in genes that are associated with the VEGF signaling, FoxO signaling, and insulin signaling pathways (see Additional file 23: Table S8). The genes in these pathways have a pivotal role in the metabolic regulation of reproduction in vertebrates. For example, the *IGF1R* gene involved in the FoxO signaling pathway encodes a tyrosine kinase-containing transmembrane protein that has a major role in cell growth control [52]. This gene was also detected in a selective sweep during chicken domestication [53].

## Discussion

### Population structure and demographic history

Similar to previous studies that used a combination of multiple genetic methods based on mtDNA data or whole-genome data [6, 14, 54, 55], the results of our analysis of population structure clearly showed two major clusters of duck populations: local Chinese populations and wild populations (Fig. 1b–d). In addition, we revealed a clear pattern of the geographical distribution of the Southeast/South Asian and Chinese duck populations. Southeast Asian populations had a closer genetic relationship with the Guangxi and South Asian populations than the other Chinese domestic populations.

Compared with the MSMC software [56], SMC++ provides more accurate estimates of Ne for the recent past, and does not require phasing of the genomic data, thus avoiding the problem of phasing errors for demographic inference [36]. Our results show that severe bottlenecks caused a general decline in population size in the Chinese and Southeast/South Asian duck populations during the Quaternary glaciations, from about 1,000,000 to 10,000 years ago, which suggests that these populations may originate from a small number of individuals that were introduced and successfully spread over a wide area after the last ice age. In recent studies, divergence times have been estimated using a diffusion approximation method for the allele frequency spectrum (∂a∂i) [15] in chicken [57], dog [58] and cattle [59]. Compared with the SFS-based inference method ∂a∂i, SMC++ can analyze pairs of populations simultaneously to infer divergence times jointly with population size histories without phasing. Because the mutation rate in *Anas platyrhynchos* is not known, we used the mutation rate of a relatively close species, i.e. the chicken, which may have introduced a slight deviation in the accuracy of the ducks’ divergence time. Our results show that divergence between the domesticated ducks and the wild ducks occurred about 1333 to 2347 years ago, which is similar to previous reports, i.e. 1787 to 2669 in [6] or ~ 1563 years ago in [14].

Southeast Asia, especially the southern part of China, is the core area of the domestication of ducks, and the history of domestic ducks in these areas covers more than 2000 years [3]. Studies have shown that there is a high degree of genetic diversity in the center of domestication of animals [60]. The lower LD values and higher genetic diversity of the Chinese populations compared to the Southeast/South Asian populations support the evidence that the southern part of China may be the core area of the domestication of ducks. Of course, gene flow that occurred between Chinese and Southeast/South Asian ducks and artificial selection should be accounted for, because they can impact the levels of diversity.

### Detecting population admixture using multiple methods

In this study, to detect gene flow, we combined multiple methods, including Admixture, *f3*-statistics, Treemix, and D-statistics based on allele frequencies, and the Globetrotter software based on haplotypes. Admixture and Treemix analyses revealed extensive gene flow within Chinese domestic populations and between Chinese wild and domestic populations, which is consistent with previous reports [6, 14]. Remarkably, the various methods used identified extensive gene flow in the Southeast/South Asian and Chinese populations.

The Guangxi population showed significant gene flow with Southeast/South Asian populations, which is highly consistent with the Admixture and D-statistics analyses. Furthermore, we identified a genomic region that has a high probability of gene flow between the Guangxi and Southeast/South Asian populations based on different statistical approaches (*fd*, *d*_*xy*_, F_ST_ and Chromopainter). Further verification revealed that this region is the product of gene flow rather than a shared ancestral polymorphism, in accordance with the theoretical expectation that gene flow reduces *d*_*xy*_ in the target regions, while shared ancestry does not [61].

However, we also observed some inconsistent results when using the above methods to detect gene flow. For example, strong genetic drift after population admixture may hamper the detection of negative *f*3-statistic values [62] between Southeast/South Asian and Guangxi populations. Compared with the Treemix results, the D-statistics were not useful to determine whether gene flow had occurred between the Cambodian and Chongqing populations. It is likely that the genetic structure of the Fujian population (P1) and the Chongqing population (P2) were too similar (Fig. 1b–d) to observe significant D-statistics (the counts of the *ABBA* pattern were very close to the counts of the *BABA* pattern). Therefore, combining allele frequency and haplotype methods to detect gene flow can help extend the results.

## Conclusions

Based on genomic data and the use of a comprehensive and efficient workflow, we found that frequent gene flows occurred between the Chinese and Southeast/South Asian duck populations and between the Southeast Asian and South Asian duck populations. We inferred the history of duck populations with different geographical distributions and showed that the Chinese and Southeast/South Asian ducks share similar demographic characteristics. An intriguing finding is that the Southeast/South Asian populations have experienced more severe bottlenecks than the Chinese populations, which has contributed to smaller effective population sizes. In addition, our analyses revealed that population structure is mostly correlated with geographic distribution. For example, the Guangxi population, which is geographically distinct from the Chinese indigenous populations and the Southeast/South Asian populations, has experienced prolonged gene flow with the Southeast/South Asian populations. Regarding the domestication of ducks, we detected strong signatures of selection in genes that are involved in signaling pathways of the nervous system development and in morphological traits such as cell growth. Our results lay the foundation for future population genetics studies in duck.

### Supplementary Information

**Additional file 1: Table S1.** Sample information for the 78 sequenced individuals and 31 individuals from NCBI.

**Additional file 2: Figure S1.** Mean pairwise F_ST_ values between groups from 14 populations.

**Additional file 3: Figure S2.** Population structure plots with K = 2 to 10. The y axis quantifies the proportion of an individual’s genome from inferred ancestral populations, and the x axis shows the different populations. Refer to Additional file 1: Table S1 for breed abbreviations.

**Additional file 4: Table S2.** Nucleotide polymorphism (θπ) from different populations.

**Additional file 5: Figure S3.** Splitting times between Chinese wild ducks and either Chinese domestic populations or Southeast/South Asian populations with 20 independent replicate analyses.

**Additional file 6: Figure S4.** The model-testing approach compared eight models using the ABC approach.

**Additional file 7: Figure S5.** Maximum likelihood based phylogenetic tree with zero to ten migration. Scale bar shows 10 times the average standard error of the estimated entries in the sample covariance matrix. Populations are colored by their geographic locations.

**Additional file 8: Figure S6.** Fraction of variance in relatedness between populations explained by phylogenetic models with zero to 12 migration events.

**Additional file 9: Table S3.** Significant (Z-score ≤ − 2) negative *f*3-statistics values testing admixture of populations.

**Additional file 10: Table S4.** Observed (H_O_) and expected heterozygosity (H_E_) values of duck samples. Refer to Additional file 1: Table S1 for breed abbreviations.

**Additional file 11: Table S5.** The D-statistics detect whether there was gene flow between Southeast Asia and South Asian populations.

**Additional file 12: Figure S7.** Clustering of individuals based on the FineStructure algorithm. The color intensity indicates shared haplotypic segments based on the chunklength coancestry matrix generated by the ChromoPainter algorithm. Refer to Additional file 1: Table S1 for breed abbreviations.

**Additional file 13: Figure S8.** Inference of the ancestry proportion of different donor populations in the genome of Guangxi, Guangdong, Guizhou and Anhui populations.

**Additional file 14: Table S6.** Eighteen extreme *fd* peaks (*fd* >  = 0.5) with the introgressed genomic regions between Guangxi and Southeast/South Asian populations using the modified f-statistic (*fd*). Introgressed genomic regions were not shorter than 200 kb.

**Additional file 15: Table S7.** Functional gene categories enriched for the genes within the introgressed genomic regions.

**Additional file 16: Figure S9.** Treemix analysis of these 18 introgressed genomic regions.

**Additional file 17: Figure S10.** Introgressed genomic regions identified in each of the Southeast/South Asian population tested were inferred by the modified f-statistic (*fd*) values. The vertical shadow line corresponds to the strongest introgressed region on chromosome 1.

**Additional file 18: Figure S11.** Mean pairwise sequence divergence (*d*_*xy*_) and population differentiation (F_ST_) around the introgressed region between the Guangxi and Southeast/South Asian populations. The vertical shadow line corresponds to the strongest introgressed region on chromosome 1.

**Additional file 19: Figure S12.** Multiple sequence alignment of the *PNPLA8* gene in birds.

**Additional file 20: Figure S13.** The decay of linkage disequilibrium in Chinese wild populations, Chinese domestic populations and Lineages (LC × BY) populations measured by *r*^2^.

**Additional file 21: Figure S14.** A neighbor-joining phylogenetic tree is constructed to understand the phylogenetic relationship of wild ducks (n = 19) and domestic ducks (n = 44) using SNPs present in regions with strong selective sweep signals.

**Additional file 22: Figure S15.** Two-way PCA plot of duck breeds to understand the relationship of wild ducks (n = 19) and domestic ducks (n = 44) using SNPs in regions with strong selective sweep signals.

**Additional file 23: Table S8.** Functional gene categories enriched for the 1048 genes with strong selective sweep signals in domestic ducks.

**Additional file 24: Table S9.** Prior distribution of the parameters used to generate the eight models in the approximate Bayesian computation (ABC) analysis.

**Additional file 25: Table S10.** Summary statistics for the observed dataset computed by the program arlsumstat.

**Additional file 26: Table S11.** Results of eight models for model choice using nine components obtained by transforming 37 summary statistics with the PLSDA method.

## Data Availability

All the new sequencing data generated in this study are deposited in the Genome Sequence Archive [66] in National Genomics Data Center [67], Beijing Institute of Genomics (China National Center for Bioinformation), Chinese Academy of Sciences, under the Accession number CRA002628 that are publicly accessible at http://bigd.big.ac.cn/gsa.

## Appendix

### ABC approach to detect gene flow

#### Data preparation and processing

To further investigate the demographic history of ducks, we leveraged the approximate Bayesian computation (ABC) approach and whole-genome sequencing data to test various competing scenarios of different divergence patterns. In our study, three populations were chosen, i.e. Chinese indigenous populations (N_CD_), Chinese wild populations (N_CW_) and Southeast/South populations (N_SEA/SA_). In addition, the pure or least admixed individuals were selected from the Admixture analysis (see section on Admixture and heterozygosity results (see Fig.1d and Additional file 10 Table S4)).

The dataset was pruned using the PLINK software (v-1.90) [32] with parameters “-geno 0.01 -maf 0.01 -indep-pairwise 50 5 0.0001”. Finally, we extracted 9311 high-quality SNPs for the three tested populations, and randomly selected 100 SNPs to calculate the summary statistics used in the subsequent modeling analyses.

To reconstruct the demographic history of ducks, we compared eight alternative demographic scenarios. Among these three populations, the amount of gene flow is 1× in Models 1, 2 and 3, 2× in Models 4, 5 and 6, 3× in Model 7, and 0 in Model 8.

#### Prior distributions of model parameters

We tested 11 demographic parameters in each of the eight models. We applied a log uniform distribution to each prior to allow for a well-distributed sampling within the range of the prior. Specifically, the effective population size (Ne) for the three groups of duck populations (N_CD_, N_CW_, and N_SEA/SA_) ranged from 100 to 100,000 (see Additional file 24: Table S9). The time parameters (T0 and T1) were set between 50 and 5000 generations. In addition, the mutation rates were set to 1.91e−9 per base per generation and the generation time was 1 year (based on an estimation from the chicken genome) [37].

#### Data simulation and choice of model

For each model, we performed 3 × 105 coalescent simulations using fastsimcoal2 [63, 64] by randomly drawing the parameter sets from the parameter space. Sample size for each population was set to 20 haplotypes (10 individuals) and simulations were performed on 20 20-kb unlinked loci. The summary statistics, calculated by arlsumstat v.3.5.2.2 [65], were used to compare the simulated and observed data. Initially, 37 summary statistics were used to describe genetic variation within (i.e., K, mean number of alleles; H, mean heterozygosity; S, number of segregating sites; Pi, nucleotide diversity) and between (F_ST_) genetic groups (see Additional file 25: Table S10). In addition, we tried to transform the 37 summary statistics into nine components using the PLSDA method [38] to capture the main information in the summary statistics and to avoid random noise that is introduced by the large number of statistics. Then, this information was used to perform an ABC-GLM standard estimation implemented in the ABCtoolbox, in order to find the most suitable gene-flow model. Model 7 showed the highest Bayes factor and posterior probability (see Additional file 26: Table S11), which means that it is the best model to explain gene-flow in duck populations.
