# Supplementary figures and images for: Analysis of whole-genome re-sequencing data of ducks reveals a diverse demographic history and extensive gene flow between Southeast/South Asian and Chinese populations
Source: Genet Sel Evol. 2021 Apr 13;53:35. doi: 10.1186/s12711-021-00627-0 (PMC8042899; doi:10.1186/s12711-021-00627-0)

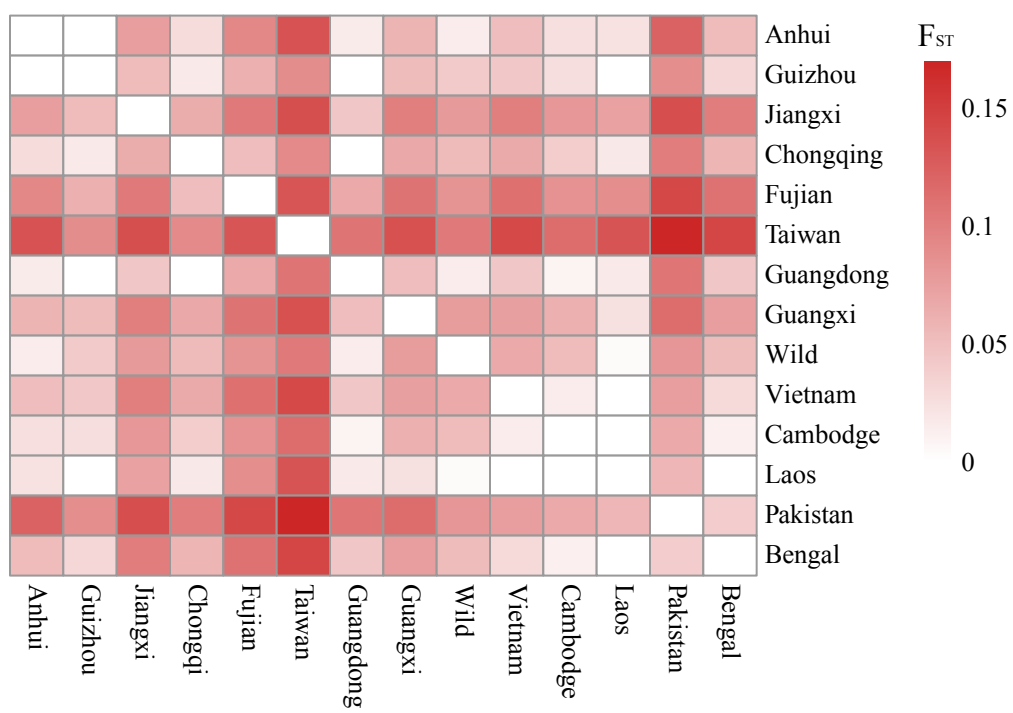

Supplement: Supplementary file 2 — Additional file 2: Figure S1. Mean pairwise FST values between groups from 14 populations. [file 12711_2021_627_MOESM2_ESM.pdf]

K=2

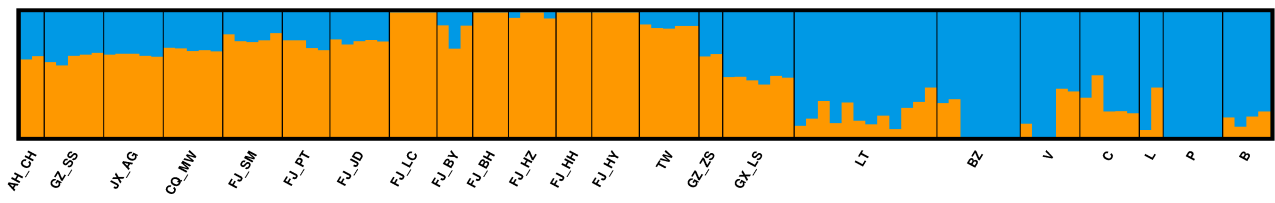

K=3

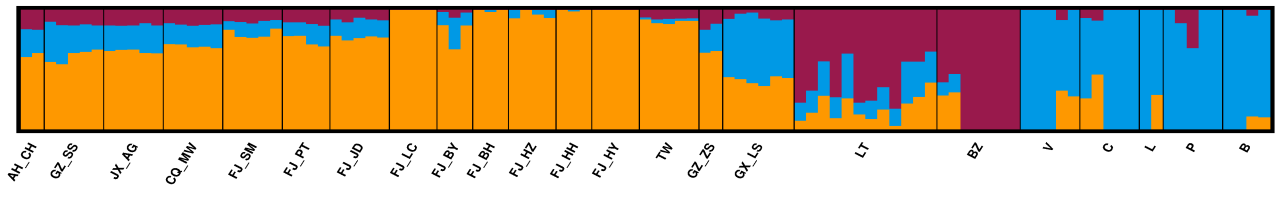

K=4

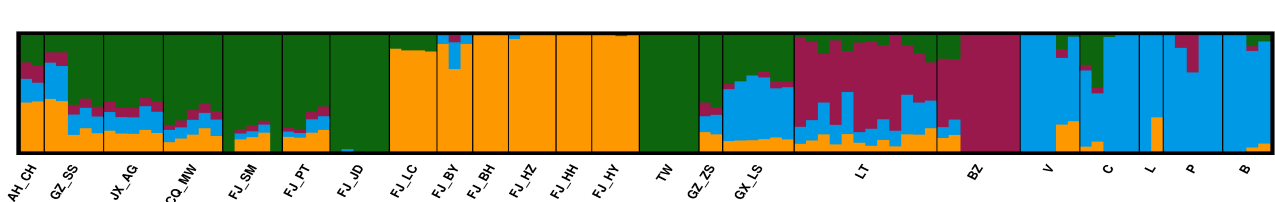

K=5

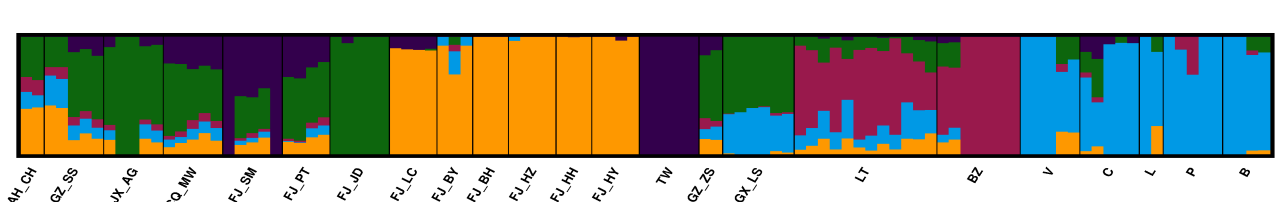

K=6

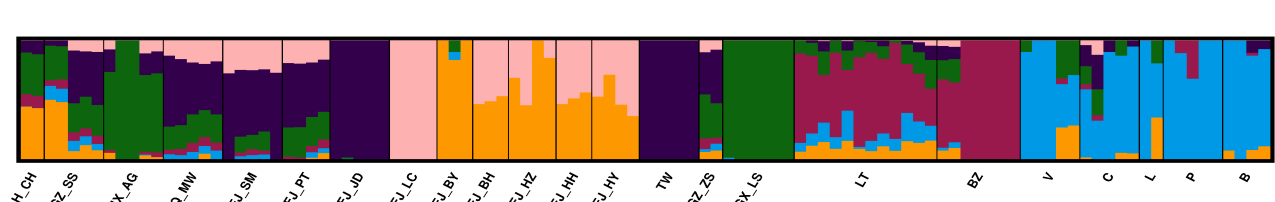

K=7

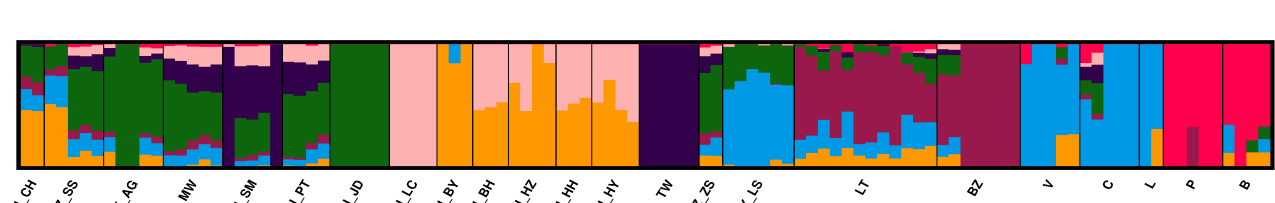

K=8

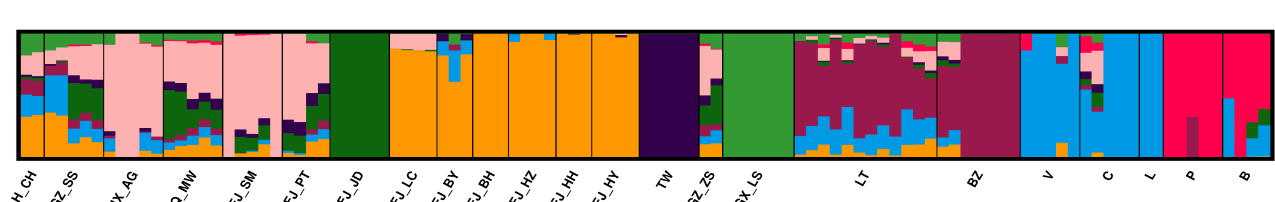

K=9

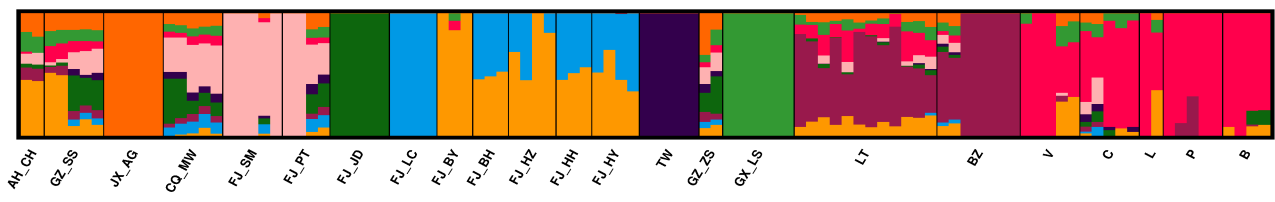

K=10

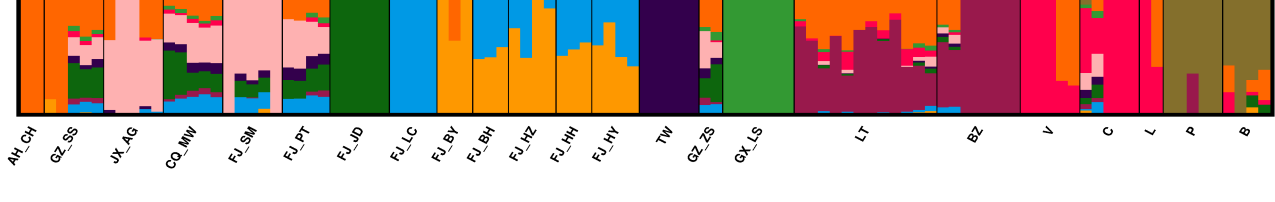

Supplement: Supplementary file 3 — Additional file 3: Figure S2. Population structure plots with K = 2 to 10. The y axis quantifies the proportion of an individual’s genome from inferred ancestral populations, and the x axis shows the different populations. Refer to Additional file 1: Table S1 for breed abbreviations. [file 12711_2021_627_MOESM3_ESM.pdf]

Years

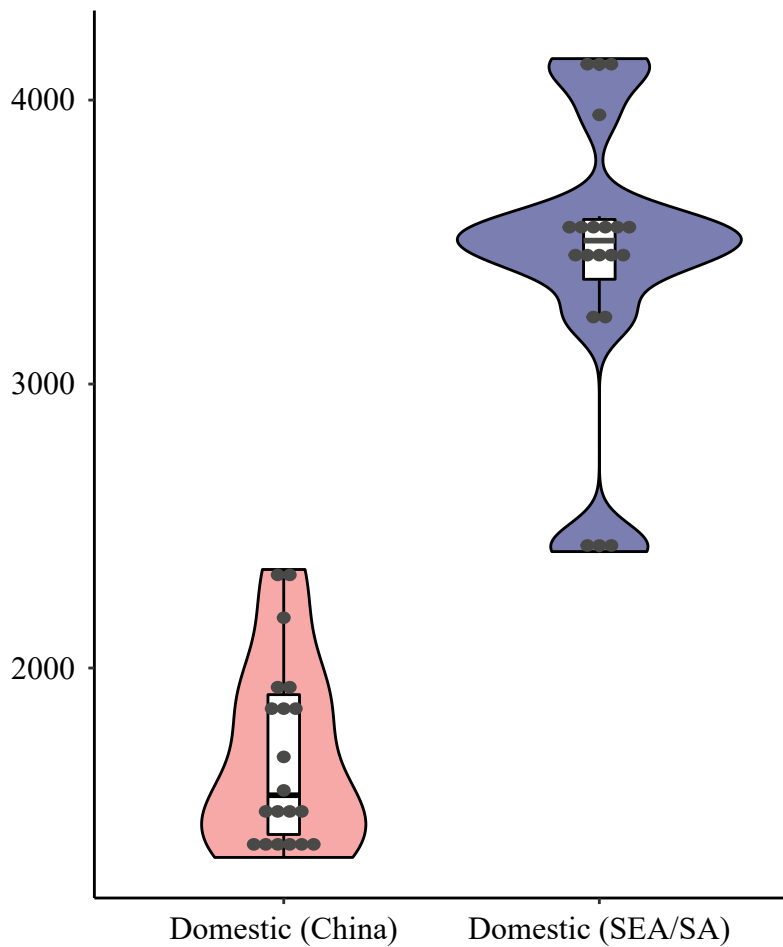

Supplement: Supplementary file 5 — Additional file 5: Figure S3. Splitting times between Chinese wild ducks and either Chinese domestic populations or Southeast/South Asian populations with 20 independent replicate analyses. [file 12711_2021_627_MOESM5_ESM.pdf]

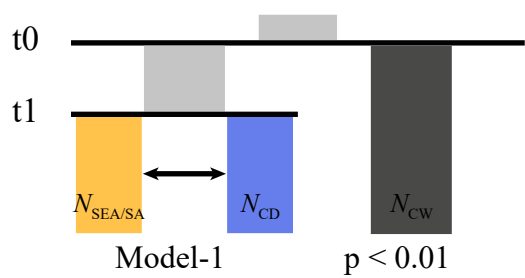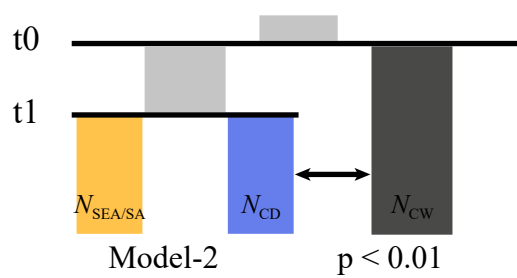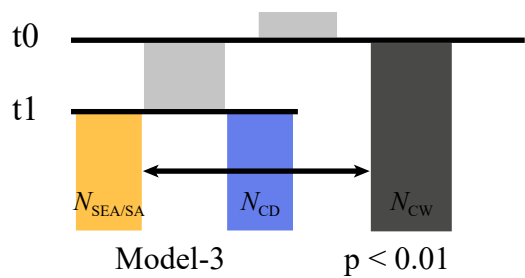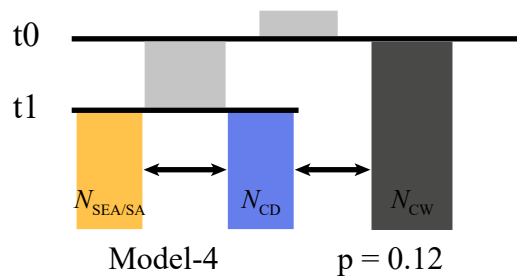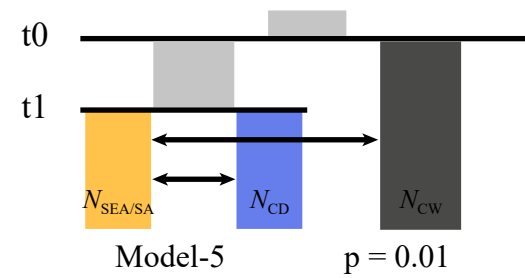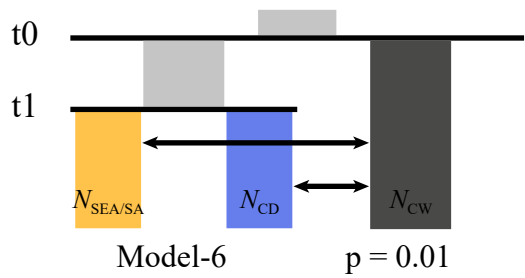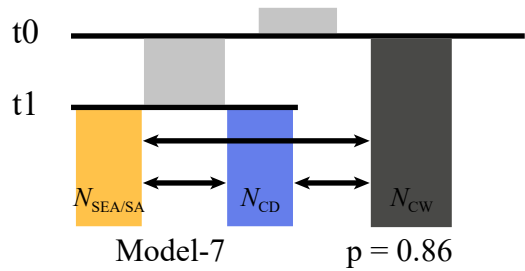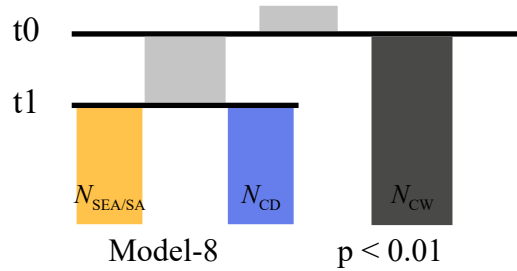

Supplement: Supplementary file 6 — Additional file 6: Figure S4. The model-testing approach compared eight models using the ABC approach. [file 12711_2021_627_MOESM6_ESM.pdf]

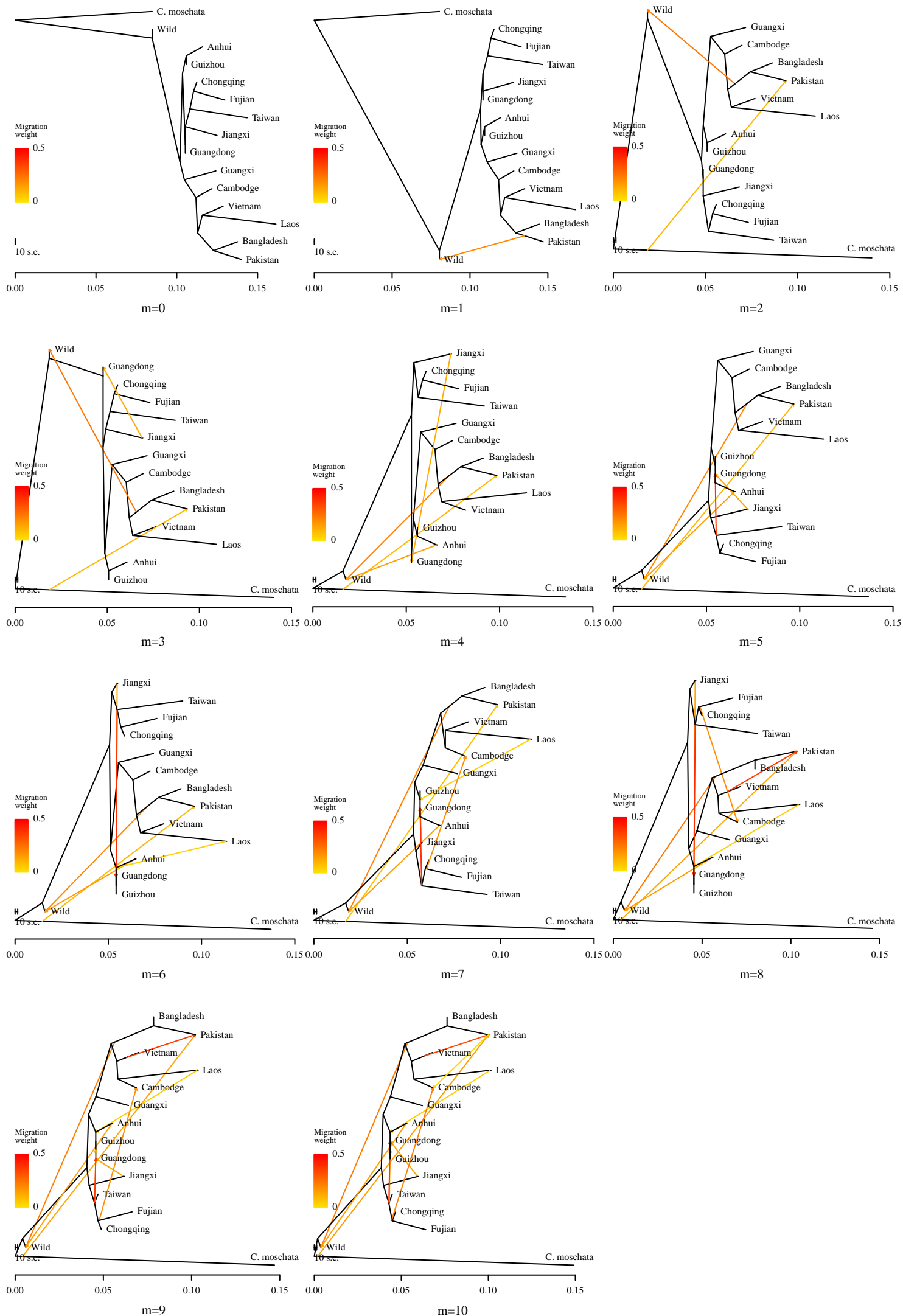

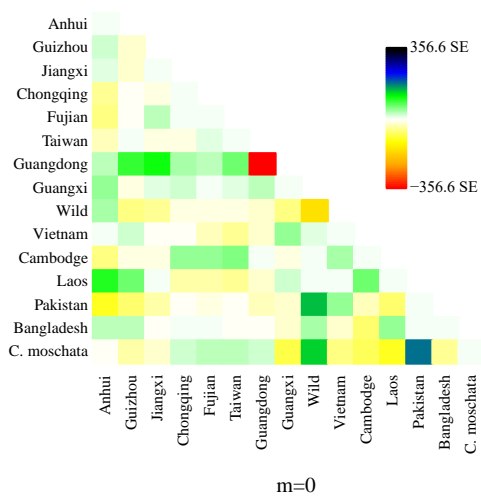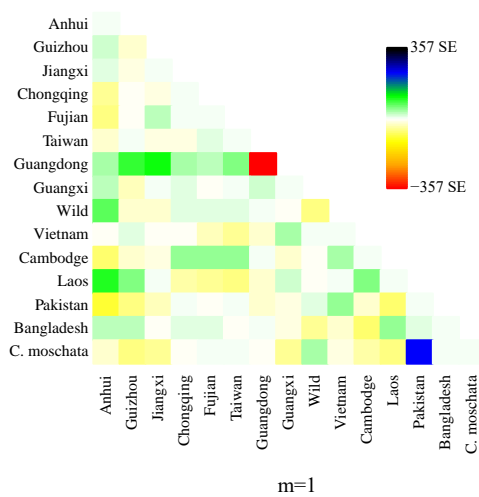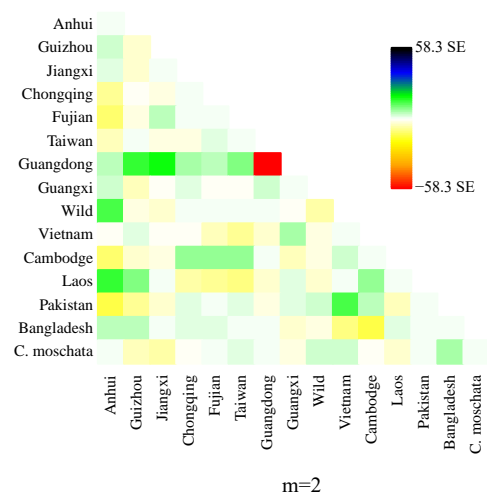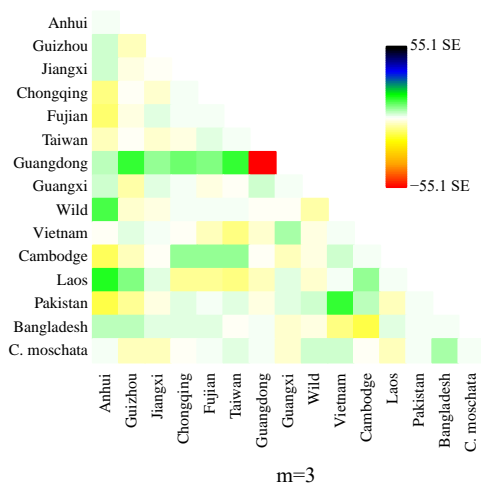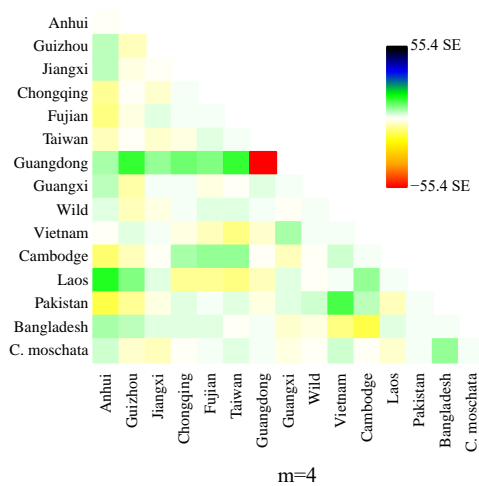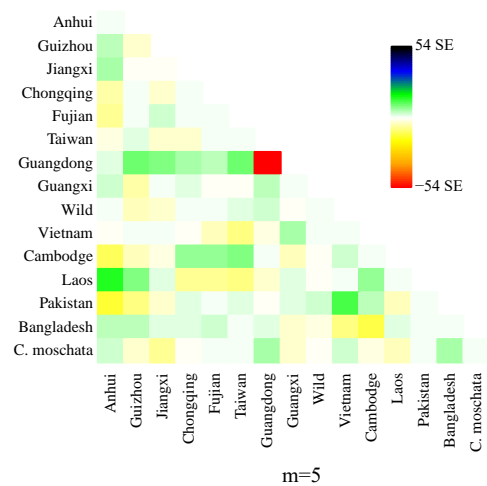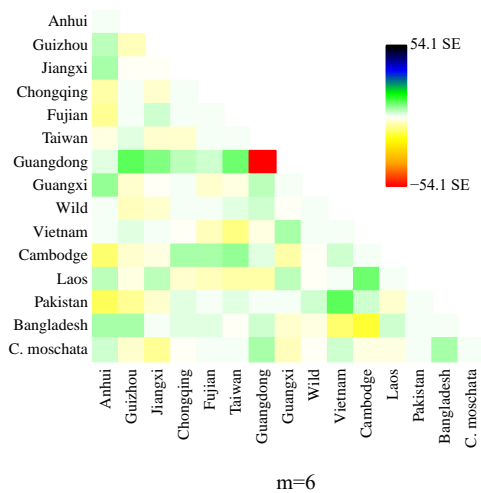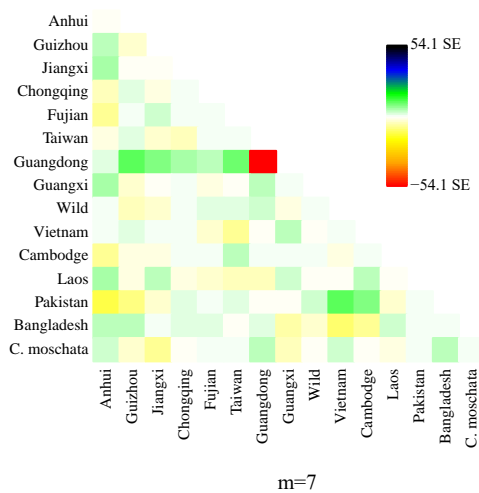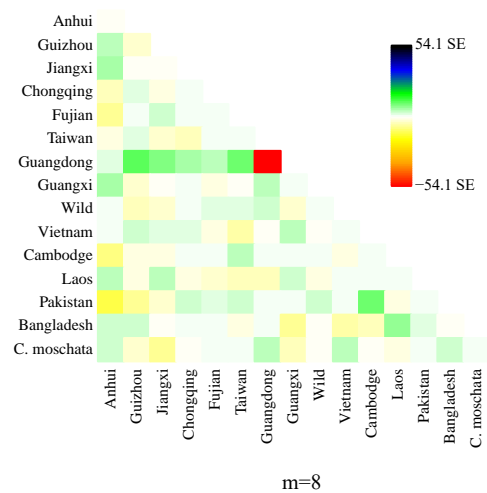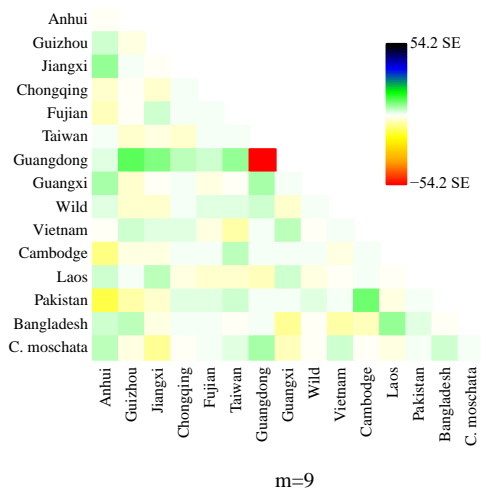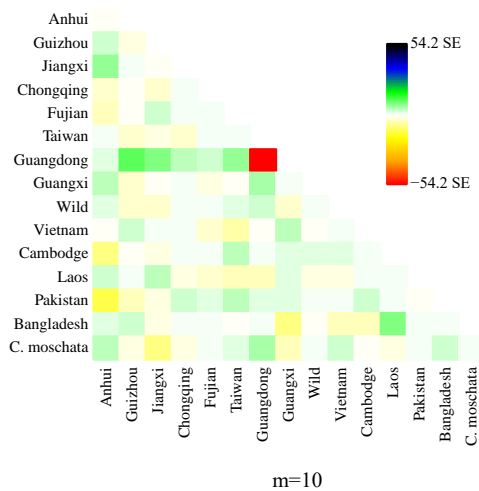

Supplement: Supplementary file 7 — Additional file 7: Figure S5. Maximum likelihood based phylogenetic tree with zero to ten migration. Scale bar shows 10 times the average standard error of the estimated entries in the sample covariance matrix. Populations are colored by their geographic locations. [file 12711_2021_627_MOESM7_ESM.pdf]

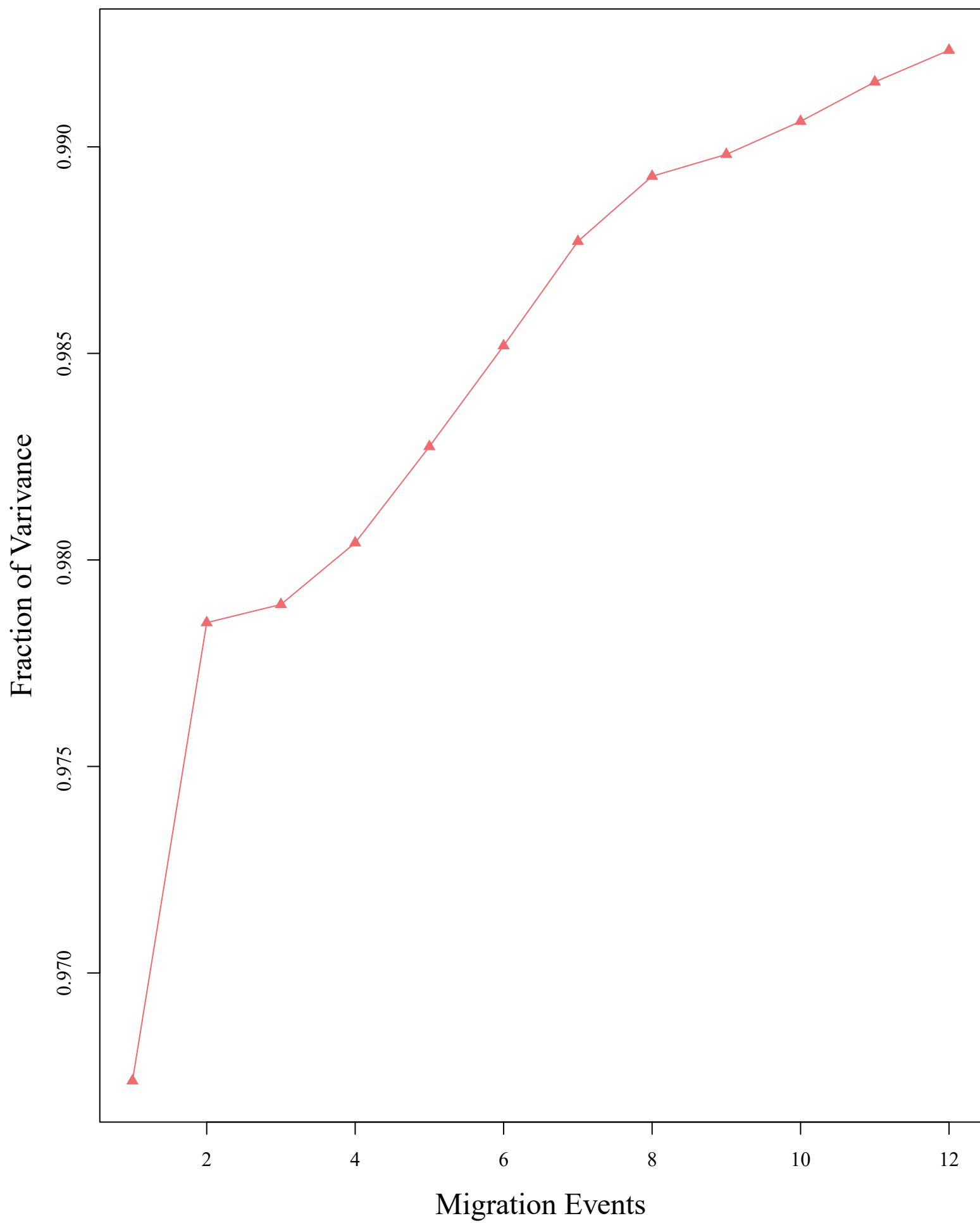

Supplement: Supplementary file 8 — Additional file 8: Figure S6. Fraction of variance in relatedness between populations explained by phylogenetic models with zero to 12 migration events. [file 12711_2021_627_MOESM8_ESM.pdf]

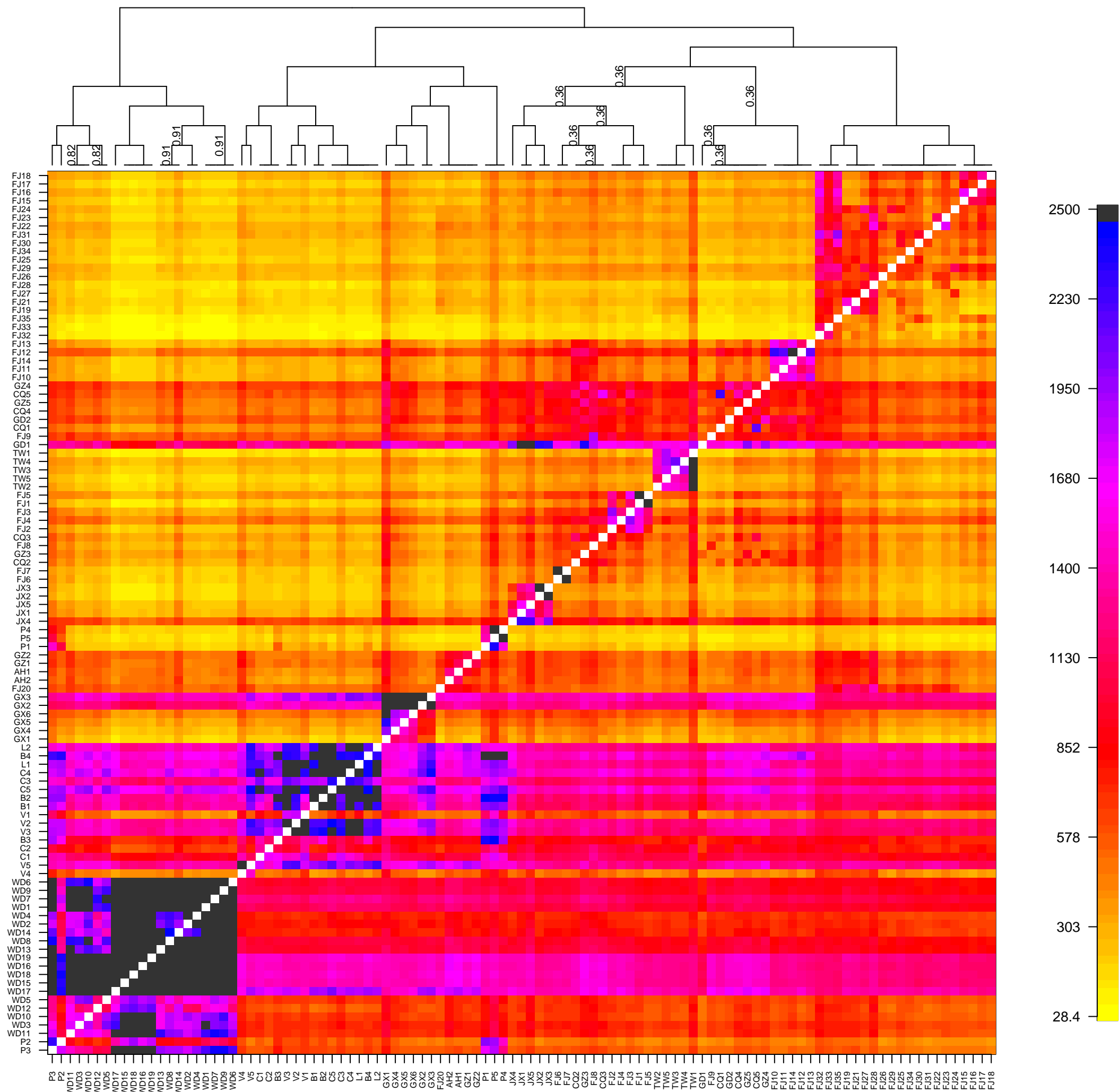

Supplement: Supplementary file 12 — Additional file 12: Figure S7. Clustering of individuals based on the FineStructure algorithm. The color intensity indicates shared haplotypic segments based on the chunklength coancestry matrix generated by the ChromoPainter algorithm. Refer to Additional file 1: Table S1 for breed abbreviations. [file 12711_2021_627_MOESM12_ESM.pdf]

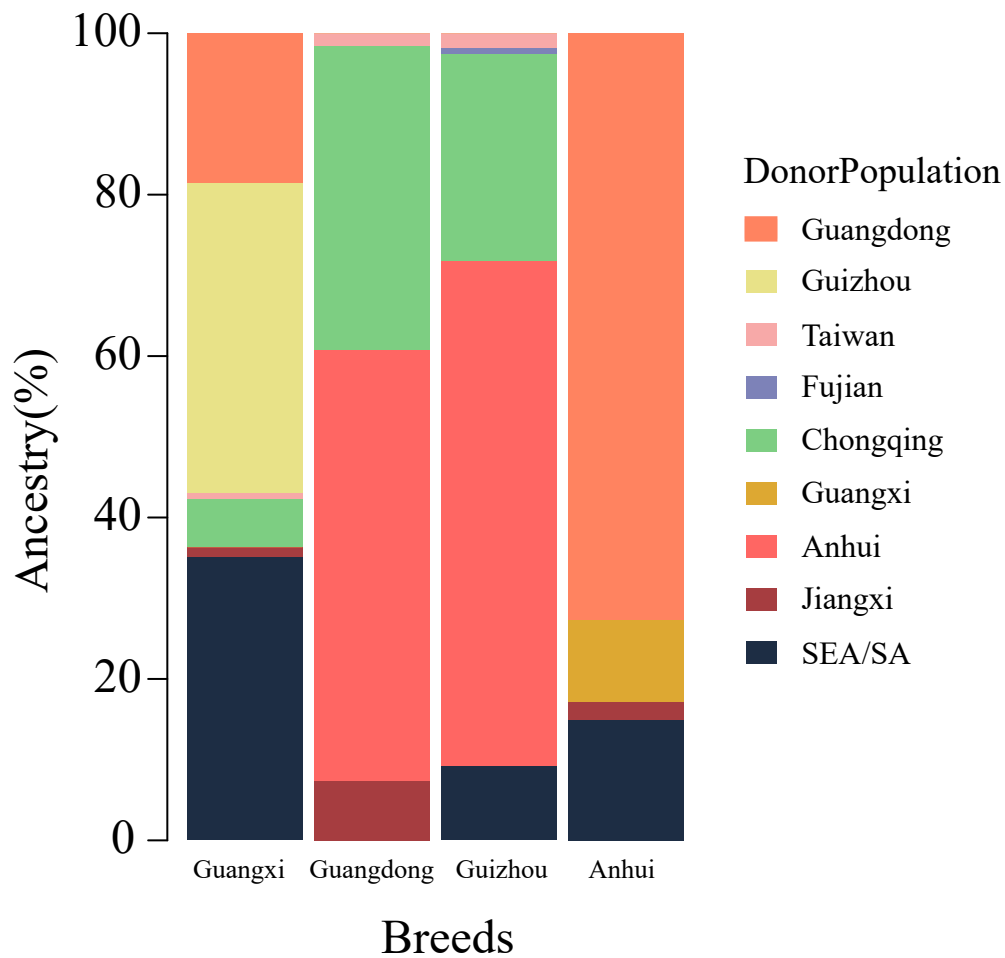

Supplement: Supplementary file 13 — Additional file 13: Figure S8. Inference of the ancestry proportion of different donor populations in the genome of Guangxi, Guangdong, Guizhou and Anhui populations. [file 12711_2021_627_MOESM13_ESM.pdf]

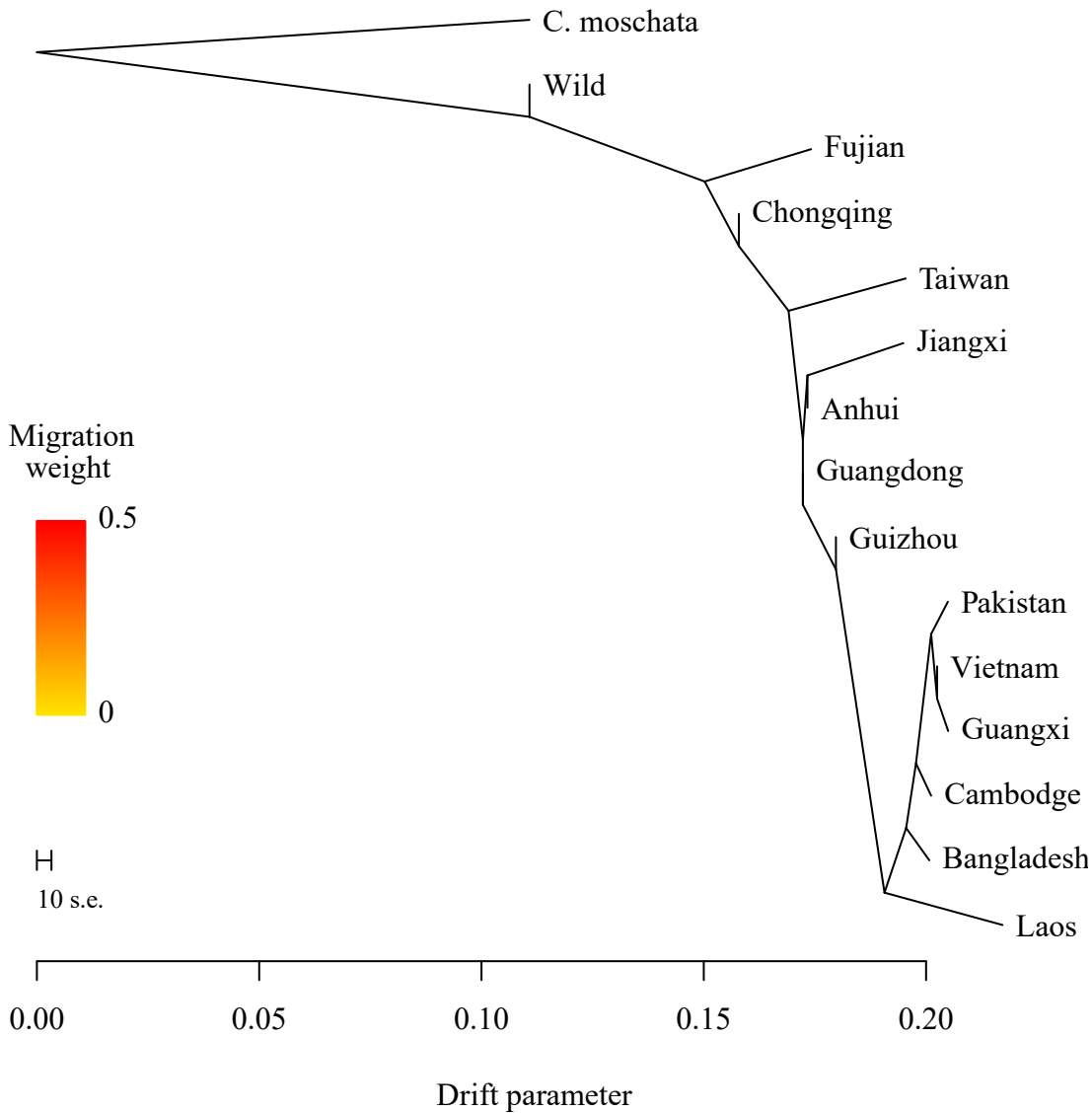

Supplement: Supplementary file 16 — Additional file 16: Figure S9. Treemix analysis of these 18 introgressed genomic regions. [file 12711_2021_627_MOESM16_ESM.pdf]

Vietnam

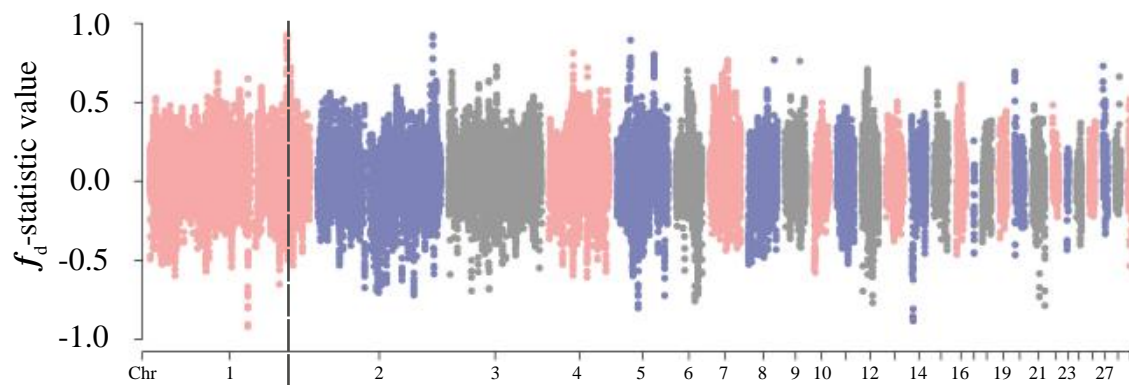

Cambodge

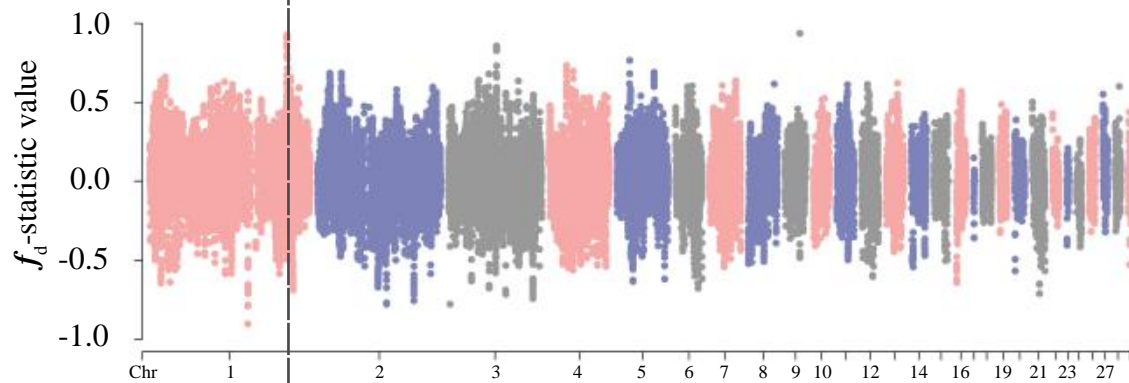

Laos

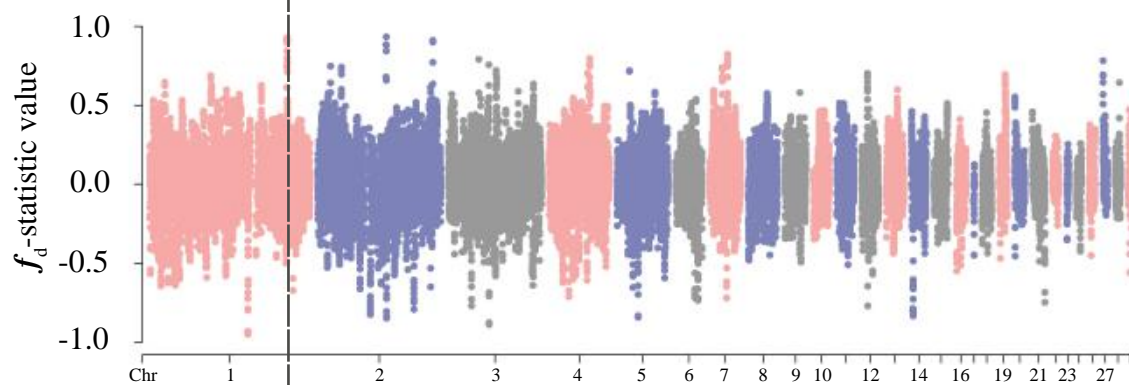

Pakistan

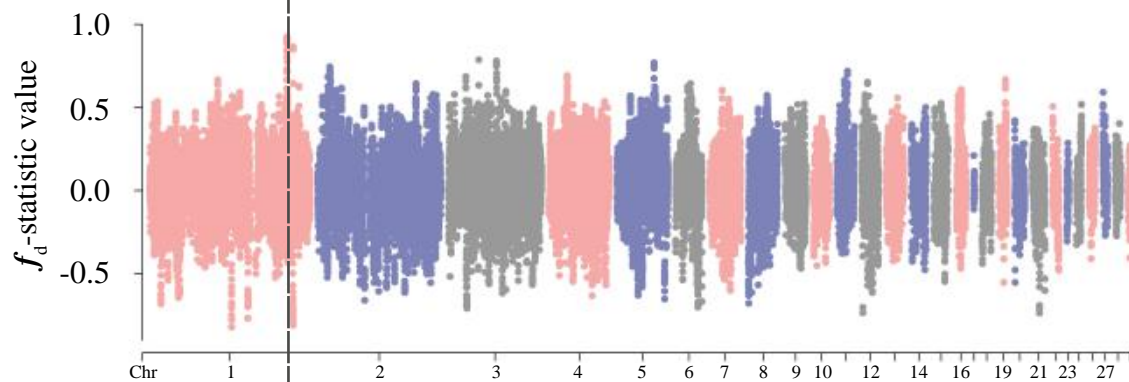

Bangladesh

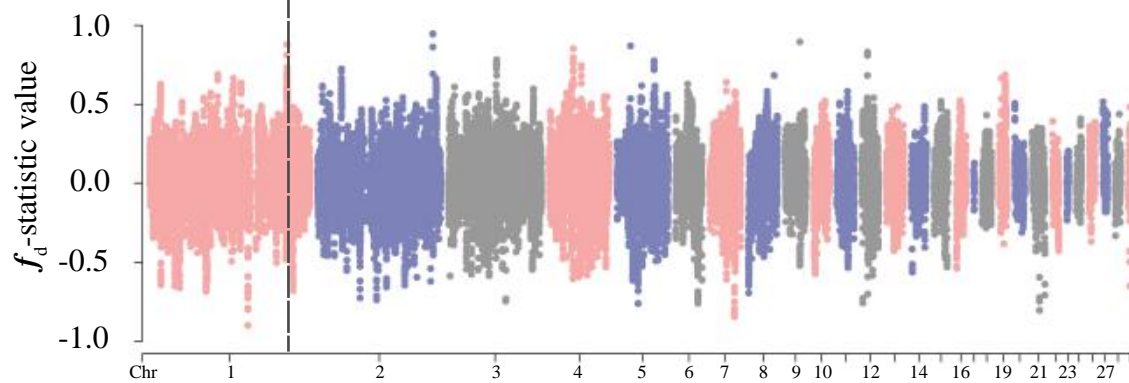

Chromosome

Supplement: Supplementary file 17 — Additional file 17: Figure S10. Introgressed genomic regions identified in each of the Southeast/South Asian population tested were inferred by the modified f-statistic (fd) values. The vertical shadow line corresponds to the strongest introgressed region on chromosome 1. [file 12711_2021_627_MOESM17_ESM.pdf]

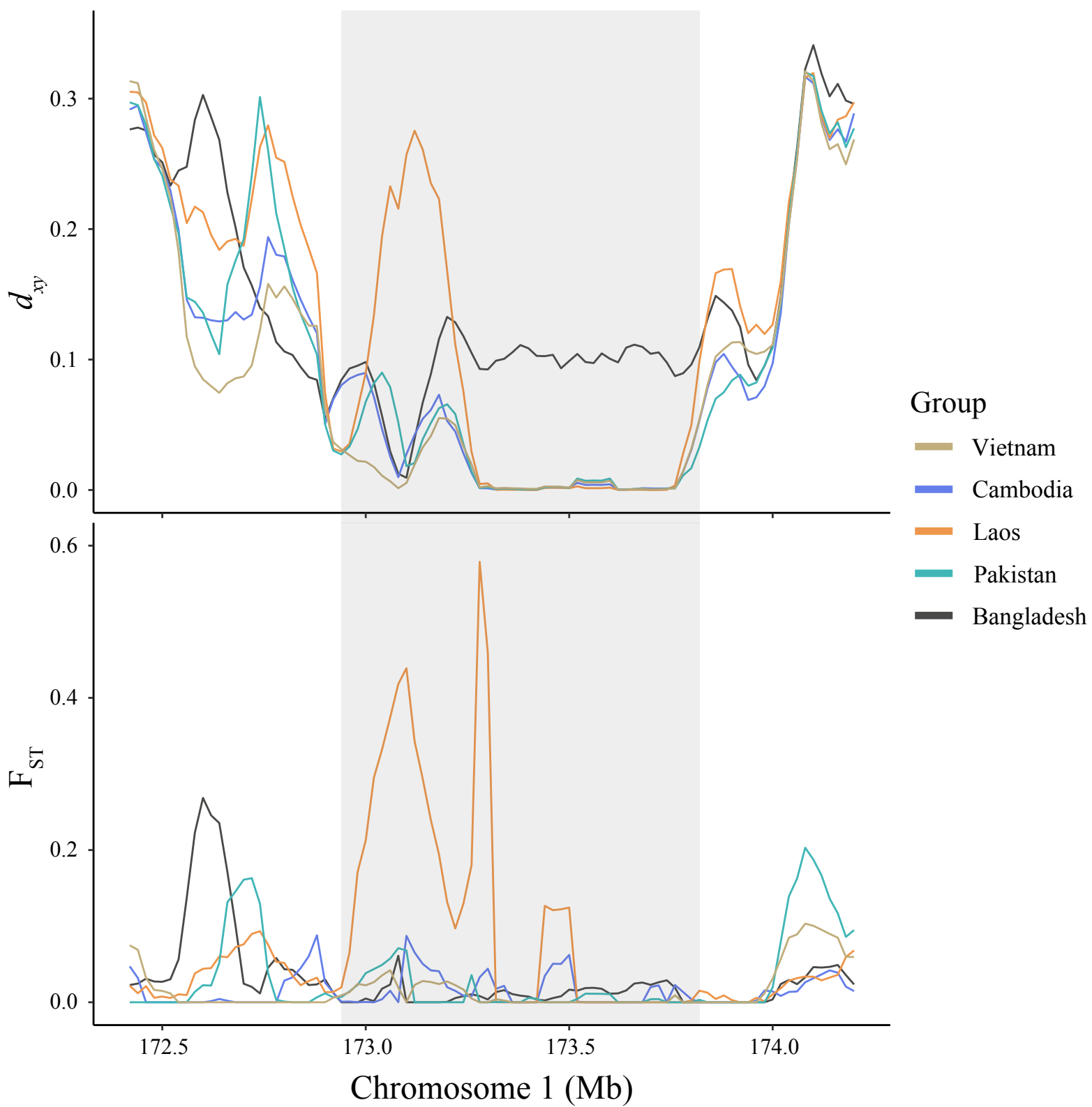

Supplement: Supplementary file 18 — Additional file 18: Figure S11. Mean pairwise sequence divergence (dxy) and population differentiation (FST) around the introgressed region between the Guangxi and Southeast/South Asian populations. The vertical shadow line corresponds to the strongest introgressed region on chromosome 1. [file 12711_2021_627_MOESM18_ESM.pdf]

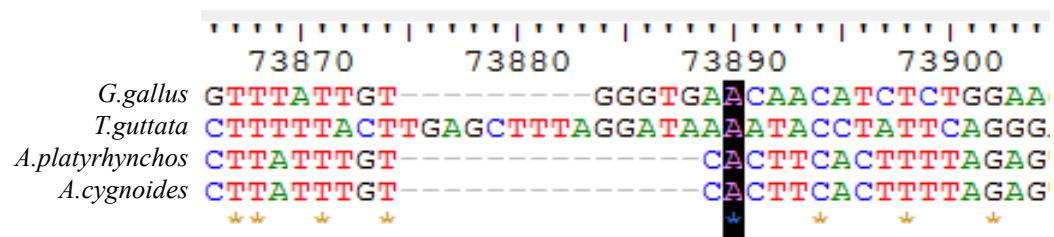

Supplement: Supplementary file 19 — Additional file 19: Figure S12. Multiple sequence alignment of the PNPLA8 gene in birds. [file 12711_2021_627_MOESM19_ESM.pdf]

## LD decay

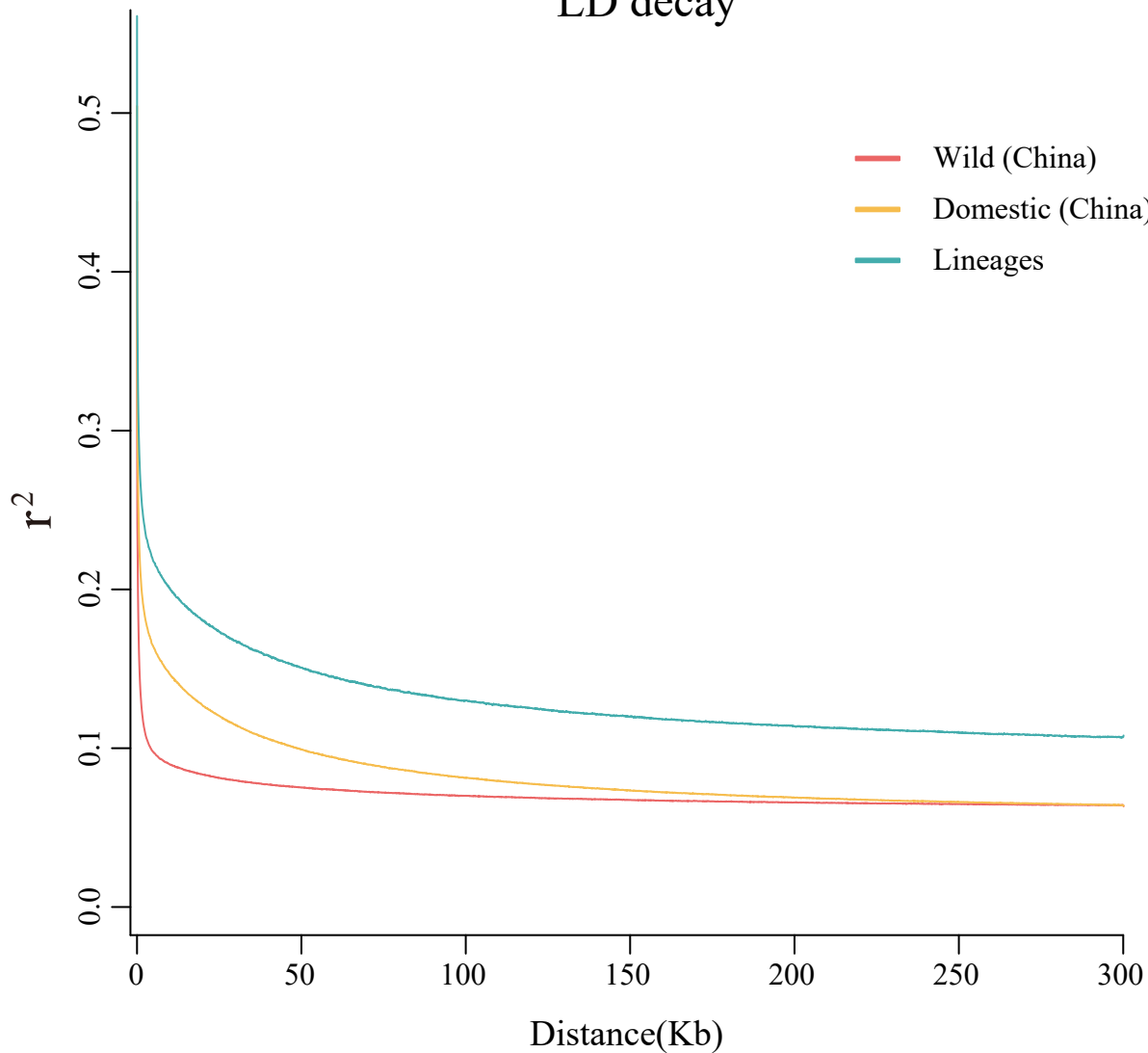

Supplement: Supplementary file 20 — Additional file 20: Figure S13. The decay of linkage disequilibrium in Chinese wild populations, Chinese domestic populations and Lineages (LC × BY) populations measured by r2. [file 12711_2021_627_MOESM20_ESM.pdf]

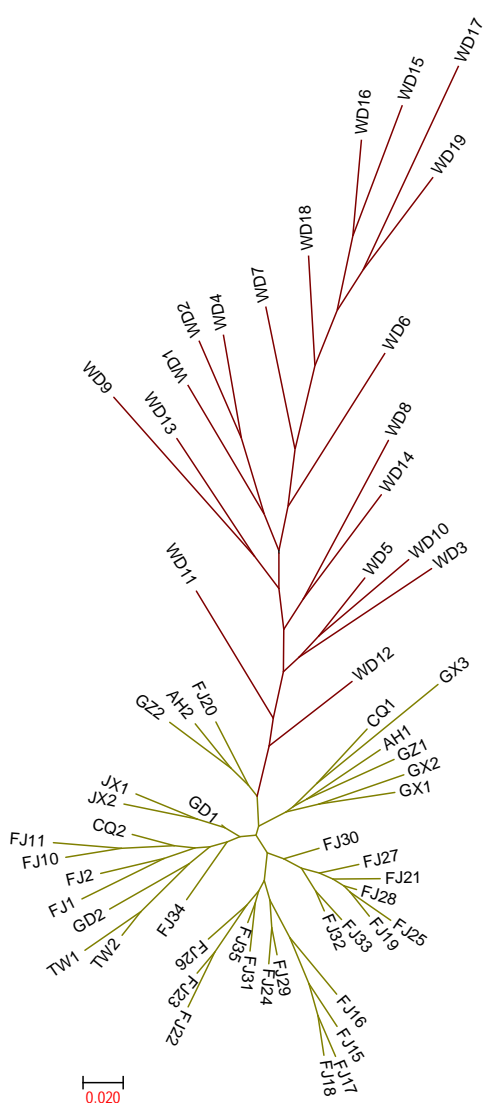

Supplement: Supplementary file 21 — Additional file 21: Figure S14. A neighbor-joining phylogenetic tree is constructed to understand the phylogenetic relationship of wild ducks (n = 19) and domestic ducks (n = 44) using SNPs present in regions with strong selective sweep signals. [file 12711_2021_627_MOESM21_ESM.pdf]

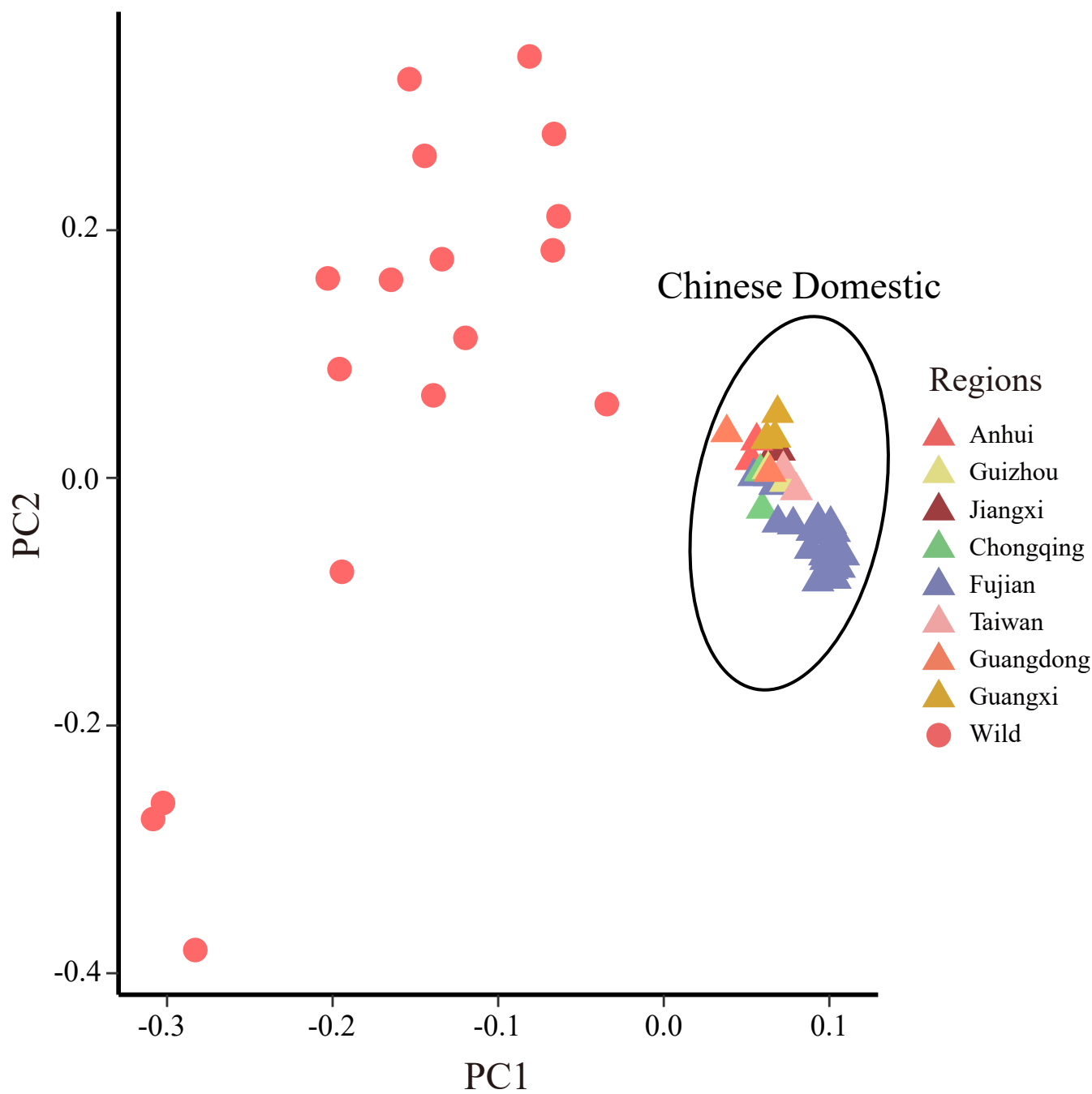

Supplement: Supplementary file 22 — Additional file 22: Figure S15. Two-way PCA plot of duck breeds to understand the relationship of wild ducks (n = 19) and domestic ducks (n = 44) using SNPs in regions with strong selective sweep signals. [file 12711_2021_627_MOESM22_ESM.pdf]
