# Supplementary material for: Analysis of whole-genome re-sequencing data of ducks reveals a diverse demographic history and extensive gene flow between Southeast/South Asian and Chinese populations
Source: Genet Sel Evol. 2021 Apr 13;53:35. doi: 10.1186/s12711-021-00627-0 (PMC8042899; doi:10.1186/s12711-021-00627-0)
Supplement: Supplementary file 4 — Additional file 4: Table S2. Nucleotide polymorphism (θπ) from different populations. [file 12711_2021_627_MOESM4_ESM.docx]

Table S2. The nucleotide polymorphism (θπ) from different populations

| **Chr** | **Wild** | **Domestic** | **Vietnam** | **Cambodge** | **Pakistan** | Bangladesh |
| --- | --- | --- | --- | --- | --- | --- |
| 1 | 0.2979 | 0.3064 | 0.2397 | 0.2473 | 0.2499 | 0.2085 |
| 2 | 0.3001 | 0.3048 | 0.2405 | 0.2466 | 0.2468 | 0.2035 |
| 3 | 0.2942 | 0.3057 | 0.2357 | 0.2462 | 0.237 | 0.2109 |
| 4 | 0.2956 | 0.3043 | 0.237 | 0.2412 | 0.25 | 0.1903 |
| 5 | 0.2999 | 0.3088 | 0.2359 | 0.2475 | 0.2423 | 0.214 |
| 6 | 0.2921 | 0.3111 | 0.2218 | 0.2409 | 0.2359 | 0.2035 |
| 7 | 0.291 | 0.3066 | 0.2229 | 0.2367 | 0.2525 | 0.1949 |
| 8 | 0.2863 | 0.3061 | 0.2209 | 0.2364 | 0.1953 | 0.1832 |
| 9 | 0.2908 | 0.3047 | 0.2095 | 0.24 | 0.2135 | 0.1942 |
| 10 | 0.2826 | 0.304 | 0.2475 | 0.2404 | 0.256 | 0.204 |
| 11 | 0.297 | 0.3067 | 0.231 | 0.2315 | 0.2134 | 0.1864 |
| 12 | 0.2744 | 0.3003 | 0.2364 | 0.251 | 0.2308 | 0.2094 |
| 13 | 0.2918 | 0.3049 | 0.2351 | 0.2327 | 0.2256 | 0.2027 |
| 14 | 0.2848 | 0.3022 | 0.2234 | 0.2402 | 0.2012 | 0.1979 |
| 15 | 0.2733 | 0.3013 | 0.2426 | 0.2494 | 0.1979 | 0.1757 |
| 16 | 0.294 | 0.3052 | 0.2251 | 0.2301 | 0.2224 | 0.2052 |
| 17 | 0.2731 | 0.3021 | 0.2156 | 0.2347 | 0.2311 | 0.1847 |
| 18 | 0.2828 | 0.2982 | 0.2235 | 0.2295 | 0.2177 | 0.1879 |
| 19 | 0.2692 | 0.2916 | 0.2299 | 0.2225 | 0.2165 | 0.1428 |
| 20 | 0.2839 | 0.3017 | 0.2249 | 0.2271 | 0.2055 | 0.1953 |
| 21 | 0.2816 | 0.2943 | 0.229 | 0.2219 | 0.1793 | 0.1968 |
| 22 | 0.2867 | 0.2995 | 0.2188 | 0.2171 | 0.2303 | 0.1796 |
| 23 | 0.2909 | 0.3059 | 0.178 | 0.2146 | 0.1798 | 0.1763 |
| 24 | 0.2776 | 0.2998 | 0.236 | 0.2266 | 0.2266 | 0.184 |
| 25 | 0.2717 | 0.2993 | 0.2037 | 0.2186 | 0.2421 | 0.182 |
| 27 | 0.2819 | 0.2933 | 0.1936 | 0.204 | 0.2049 | 0.1717 |
| 28 | 0.2835 | 0.3059 | 0.2472 | 0.2346 | 0.2508 | 0.1785 |
| 29 | 0.2881 | 0.2955 | 0.2106 | 0.2385 | 0.2575 | 0.175 |
