# Supplementary material for: Analysis of whole-genome re-sequencing data of ducks reveals a diverse demographic history and extensive gene flow between Southeast/South Asian and Chinese populations
Source: Genet Sel Evol. 2021 Apr 13;53:35. doi: 10.1186/s12711-021-00627-0 (PMC8042899; doi:10.1186/s12711-021-00627-0)
Supplement: Supplementary file 9 — Additional file 9: Table S3. Significant (Z-score ≤ − 2) negative f3-statistics values testing admixture of populations. [file 12711_2021_627_MOESM9_ESM.docx]

Table S3. Significant (Z-score ≤ -2) negative *f3*-statistics values testing admixture of populations

| **Population A** | **Population B** | **Population C** | ***f_3_*** | **Standard Error** | **Z-score** |
| --- | --- | --- | --- | --- | --- |
| Guizhou | Anhui | Chongqing | -0.00165485 | 0.000220024 | -7.52122 |
| Guizhou | Anhui | Fujian | -0.00177303 | 0.000258099 | -6.86956 |
| Guizhou | Anhui | Taiwan | -0.00126574 | 0.000280663 | -4.50984 |
| Guizhou | Anhui | Guangdong | -0.00189341 | 0.000233054 | -8.12435 |
| Guizhou | Anhui | Vietnam | -0.000908291 | 0.000260071 | -3.49247 |
| Guizhou | Anhui | Cambodia | -0.0015596 | 0.000243451 | -6.40624 |
| Guizhou | Anhui | Pakistan | -0.00137638 | 0.00028618 | -4.80949 |
| Guizhou | Guangdong | Vietnam | -0.000658344 | 0.000228931 | -2.87573 |
| Guizhou | Guangdong | Laos | -0.00189056 | 0.000247963 | -7.62437 |
| Guizhou | Guangdong | Bangladesh | -0.000486382 | 0.000233455 | -2.08341 |
| Guangdong | Anhui | Guizhou | -0.00466358 | 0.000289939 | -16.0847 |
| Guangdong | Anhui | Jiangxi | -0.00978237 | 0.000319058 | -30.6601 |
| Guangdong | Anhui | Chongqing | -0.00946926 | 0.000289288 | -32.733 |
| Guangdong | Anhui | Fujian | -0.00951224 | 0.000339562 | -28.0132 |
| Guangdong | Anhui | Taiwan | -0.00954452 | 0.000359398 | -26.557 |
| Guangdong | Anhui | Guangxi | -0.00521239 | 0.000340361 | -15.3143 |
| Guangdong | Anhui | Wild | -0.00401784 | 0.00027795 | -14.4552 |
| Guangdong | Anhui | Vietnam | -0.00491352 | 0.000370111 | -13.2758 |
| Guangdong | Anhui | Cambodia | -0.00696749 | 0.000330804 | -21.0623 |
| Guangdong | Anhui | Laos | -0.00235433 | 0.000408517 | -5.76312 |
| Guangdong | Anhui | Pakistan | -0.00716913 | 0.000381632 | -18.7855 |
| Guangdong | Anhui | Bangladesh | -0.00467471 | 0.000359944 | -12.9873 |
| Guangdong | Anhui | WY | -0.00614269 | 0.000307089 | -20.0029 |
| Guangdong | Guizhou | Jiangxi | -0.0120086 | 0.0002402 | -49.9941 |
| Guangdong | Guizhou | Chongqing | -0.00970782 | 0.000230478 | -42.1205 |
| Guangdong | Guizhou | Fujian | -0.00963263 | 0.000266569 | -36.1356 |
| Guangdong | Guizhou | Taiwan | -0.0101722 | 0.000272966 | -37.2654 |
| Guangdong | Guizhou | Guangxi | -0.00801443 | 0.000262848 | -30.4908 |
| Guangdong | Guizhou | Wild | -0.00782951 | 0.000208911 | -37.4777 |
| Guangdong | Guizhou | Vietnam | -0.00589865 | 0.000280325 | -21.0422 |
| Guangdong | Guizhou | Cambodia | -0.0073013 | 0.000257624 | -28.3409 |
| Guangdong | Guizhou | Laos | -0.00466643 | 0.000312494 | -14.9328 |
| Guangdong | Guizhou | Pakistan | -0.00768617 | 0.000289915 | -26.5118 |
| Guangdong | Guizhou | Bangladesh | -0.00607061 | 0.000276438 | -21.9601 |
| Guangdong | Guizhou | WY | -0.00845542 | 0.00022487 | -37.6014 |
| Guangdong | Jiangxi | Chongqing | -0.0104237 | 0.000260451 | -40.0218 |
| Guangdong | Jiangxi | Fujian | -0.00929491 | 0.000301731 | -30.8053 |
| Guangdong | Jiangxi | Taiwan | -0.0110766 | 0.000312637 | -35.4297 |
| Guangdong | Jiangxi | Guangxi | -0.00976311 | 0.000287179 | -33.9966 |
| Guangdong | Jiangxi | Wild | -0.0098846 | 0.000220304 | -44.868 |
| Guangdong | Jiangxi | Vietnam | -0.0088492 | 0.000305076 | -29.0066 |
| Guangdong | Jiangxi | Cambodia | -0.00965536 | 0.00028173 | -34.2717 |
| Guangdong | Jiangxi | Laos | -0.00858992 | 0.000330482 | -25.9921 |
| Guangdong | Jiangxi | Pakistan | -0.00956419 | 0.0003171 | -30.1615 |
| Guangdong | Jiangxi | Bangladesh | -0.00936734 | 0.000302003 | -31.0174 |
| Guangdong | Jiangxi | WY | -0.0103995 | 0.000243218 | -42.758 |
| Guangdong | Chongqing | Fujian | -0.00260557 | 0.000296014 | -8.80218 |
| Guangdong | Chongqing | Taiwan | -0.00611747 | 0.000303832 | -20.1344 |
| Guangdong | Chongqing | Guangxi | -0.00760256 | 0.000271868 | -27.9642 |
| Guangdong | Chongqing | Wild | -0.00728603 | 0.000215288 | -33.8431 |
| Guangdong | Chongqing | Vietnam | -0.0069739 | 0.000287352 | -24.2696 |
| Guangdong | Chongqing | Cambodia | -0.00625504 | 0.000273875 | -22.8391 |
| Guangdong | Chongqing | Laos | -0.00778865 | 0.000306654 | -25.3988 |
| Guangdong | Chongqing | Pakistan | -0.00676455 | 0.000304789 | -22.1942 |
| Guangdong | Chongqing | Bangladesh | -0.0071581 | 0.000283798 | -25.2225 |
| Guangdong | Chongqing | WY | -0.00749947 | 0.00024081 | -31.1427 |
| Guangdong | Fujian | Taiwan | -0.00526483 | 0.000355086 | -14.8269 |
| Guangdong | Fujian | Guangxi | -0.00781983 | 0.000311897 | -25.0718 |
| Guangdong | Fujian | Wild | -0.00704519 | 0.000251124 | -28.0547 |
| Guangdong | Fujian | Vietnam | -0.00737473 | 0.000333918 | -22.0855 |
| Guangdong | Fujian | Cambodia | -0.00600074 | 0.000319062 | -18.8075 |
| Guangdong | Fujian | Laos | -0.00770201 | 0.000363432 | -21.1924 |
| Guangdong | Fujian | Pakistan | -0.00673936 | 0.000360948 | -18.6713 |
| Guangdong | Fujian | Bangladesh | -0.00691967 | 0.000334988 | -20.6565 |
| Guangdong | Fujian | WY | -0.00704787 | 0.000281355 | -25.0498 |
| Guangdong | Taiwan | Guangxi | -0.00848861 | 0.00032534 | -26.0915 |
| Guangdong | Taiwan | Wild | -0.00782895 | 0.00026066 | -30.0351 |
| Guangdong | Taiwan | Vietnam | -0.00872704 | 0.000348983 | -25.0071 |
| Guangdong | Taiwan | Cambodia | -0.00670948 | 0.000325887 | -20.5884 |
| Guangdong | Taiwan | Laos | -0.00873268 | 0.000375969 | -23.2271 |
| Guangdong | Taiwan | Pakistan | -0.00733498 | 0.000377473 | -19.4318 |
| Guangdong | Taiwan | Bangladesh | -0.00811688 | 0.000352103 | -23.0526 |
| Guangdong | Taiwan | WY | -0.00776284 | 0.000296614 | -26.1715 |
| Guangdong | Guangxi | Wild | -0.00483632 | 0.000251721 | -19.213 |
| Guangdong | Guangxi | Cambodia | -0.00152505 | 0.000317223 | -4.80751 |
| Guangdong | Guangxi | Bangladesh | -0.00137255 | 0.000333128 | -4.1202 |
| Guangdong | Guangxi | WY | -0.00643088 | 0.000282233 | -22.7857 |
| Guangdong | Wild | Vietnam | -0.00164634 | 0.000280487 | -5.86955 |
| Guangdong | Wild | Cambodia | -0.00256424 | 0.000258712 | -9.91159 |
| Guangdong | Wild | Laos | -0.0017372 | 0.000305096 | -5.69395 |
| Guangdong | Wild | Bangladesh | -0.00136242 | 0.000277278 | -4.91355 |
| Guangdong | Vietnam | WY | -0.00448056 | 0.000306806 | -14.6039 |
| Guangdong | Cambodia | WY | -0.00565457 | 0.000282442 | -20.0203 |
| Guangdong | Laos | WY | -0.00561882 | 0.000335922 | -16.7266 |
| Guangdong | Bangladesh | WY | -0.00467574 | 0.00030654 | -15.2533 |
| Wild | Anhui | WY | -0.00785077 | 0.000148659 | -52.8107 |
| Wild | Guizhou | WY | -0.00635182 | 0.00012011 | -52.8835 |
| Wild | Jiangxi | WY | -0.00624083 | 0.000140719 | -44.3496 |
| Wild | Chongqing | WY | -0.00593936 | 0.000125778 | -47.2209 |
| Wild | Fujian | WY | -0.0057286 | 0.000148181 | -38.6594 |
| Wild | Taiwan | WY | -0.00565981 | 0.000160062 | -35.36 |
| Wild | Guangdong | WY | -0.00572592 | 0.000133952 | -42.7459 |
| Wild | Guangxi | WY | -0.00732048 | 0.000138294 | -52.9342 |
| Wild | Vietnam | WY | -0.00856014 | 0.000139998 | -61.1446 |
| Wild | Cambodia | WY | -0.00881624 | 0.00012976 | -67.9427 |
| Wild | Laos | WY | -0.00960754 | 0.000158515 | -60.6097 |
| Wild | Pakistan | WY | -0.00594883 | 0.000148954 | -39.9375 |
| Wild | Bangladesh | WY | -0.00903924 | 0.000144357 | -62.6173 |
