# Supplementary material for: Analysis of whole-genome re-sequencing data of ducks reveals a diverse demographic history and extensive gene flow between Southeast/South Asian and Chinese populations
Source: Genet Sel Evol. 2021 Apr 13;53:35. doi: 10.1186/s12711-021-00627-0 (PMC8042899; doi:10.1186/s12711-021-00627-0)
Supplement: Supplementary file 10 — Additional file 10: Table S4. Observed (HO) and expected heterozygosity (HE) values of duck samples. Refer to Additional file 1: Table S1 for breed abbreviations. [file 12711_2021_627_MOESM10_ESM.docx]

Table S4. Observed (HO) and expected heterozygosity (HE) values of duck samples. Refer to Table S1 for the breed abbreviations

| **INDV** | **O(HOM)** | **E(HOM)** | **N_SITES** | **HO** | **HE** |
| --- | --- | --- | --- | --- | --- |
| AH1 | 860314 | 902020.3 | 2016226 | 0.573304778 | 0.552619448 |
| AH2 | 909528 | 902020.3 | 2016978 | 0.549063996 | 0.552786248 |
| GZ1 | 2083937 | 1945824.8 | 3148830 | 0.338186882 | 0.382048316 |
| GZ2 | 2113236 | 1946367.6 | 3149736 | 0.329075199 | 0.382053734 |
| GZ3 | 1967793 | 1948796.2 | 3153993 | 0.376094684 | 0.382117779 |
| GZ4 | 2143076 | 1930207.2 | 3121614 | 0.313471813 | 0.381663716 |
| GZ5 | 2031623 | 1940035.4 | 3138805 | 0.352739976 | 0.381919106 |
| JX1 | 1521104 | 1616616.7 | 2698203 | 0.436252943 | 0.400854309 |
| JX2 | 1484585 | 1616422 | 2697868 | 0.449719186 | 0.40085208 |
| JX3 | 1552208 | 1613991.3 | 2693512 | 0.423723377 | 0.400785554 |
| JX4 | 1766536 | 1596365.5 | 2662347 | 0.336474171 | 0.400391647 |
| JX5 | 1639252 | 1616267.1 | 2697595 | 0.392328352 | 0.400848867 |
| CQ1 | 1905265 | 1904867 | 3100532 | 0.385503843 | 0.385632208 |
| CQ2 | 1834175 | 1907104.6 | 3104474 | 0.409183327 | 0.385691554 |
| CQ3 | 1930759 | 1905261.4 | 3101298 | 0.37743519 | 0.38565678 |
| CQ4 | 1961419 | 1898776.6 | 3089951 | 0.365226504 | 0.385499446 |
| CQ5 | 2035347 | 1887728.5 | 3070583 | 0.337146399 | 0.385221471 |
| FJ1 | 2767982 | 2719681.6 | 3923912 | 0.294586117 | 0.306895364 |
| FJ2 | 2786019 | 2719713.6 | 3923966 | 0.2899992 | 0.306896747 |
| FJ3 | 2794371 | 2712005.7 | 3912306 | 0.285748354 | 0.306801232 |
| FJ4 | 2931483 | 2705911.3 | 3903209 | 0.248955667 | 0.306747013 |
| FJ5 | 2898239 | 2699624.4 | 3893302 | 0.255583307 | 0.306597741 |
| FJ6 | 2988326 | 2719414.7 | 3923531 | 0.238357999 | 0.306896084 |
| FJ7 | 3023610 | 2718814.9 | 3922633 | 0.22918866 | 0.306890321 |
| FJ8 | 2812637 | 2719893.3 | 3924238 | 0.283265439 | 0.306898995 |
| FJ9 | 2696116 | 2717404.7 | 3920485 | 0.312300391 | 0.306870272 |
| FJ10 | 2955695 | 2716580.4 | 3919257 | 0.245853232 | 0.306863418 |
| FJ11 | 2849643 | 2718462.1 | 3922097 | 0.273438928 | 0.306885551 |
| FJ12 | 3041820 | 2696204.9 | 3888321 | 0.217703477 | 0.306588911 |
| FJ13 | 3066380 | 2714000.6 | 3915310 | 0.216823189 | 0.306823572 |
| FJ14 | 2992622 | 2713702 | 3915024 | 0.235605708 | 0.306849205 |
| FJ15 | 2964014 | 2717829.9 | 3921097 | 0.24408552 | 0.306870016 |
| FJ16 | 3020136 | 2716925.6 | 3919663 | 0.229490903 | 0.306847145 |
| FJ17 | 2944811 | 2719409.5 | 3923509 | 0.249444566 | 0.306893523 |
| FJ18 | 2862224 | 2718529 | 3922142 | 0.270239578 | 0.306876447 |
| FJ19 | 2846127 | 2719961.7 | 3924329 | 0.274748116 | 0.306897638 |
| FJ20 | 2856134 | 2717068.2 | 3919949 | 0.271384908 | 0.306861339 |
| FJ21 | 2779024 | 2720300.6 | 3924822 | 0.291936297 | 0.306898351 |
| FJ22 | 2895567 | 2716726.3 | 3919366 | 0.261215462 | 0.306845469 |
| FJ23 | 2769025 | 2717488.2 | 3920523 | 0.293710303 | 0.306855692 |
| FJ24 | 2789733 | 2719484.4 | 3923592 | 0.28898494 | 0.306889096 |
| FJ25 | 2718767 | 2719639.2 | 3923826 | 0.307113261 | 0.306890978 |
| FJ26 | 2828521 | 2718990.1 | 3922832 | 0.278959435 | 0.30688082 |
| FJ27 | 3084285 | 2719990.2 | 3924377 | 0.214070157 | 0.306898853 |
| FJ28 | 2837079 | 2720244.5 | 3924742 | 0.277129809 | 0.306898517 |
| FJ29 | 2783575 | 2716175.5 | 3918510 | 0.289634325 | 0.306834613 |
| FJ30 | 2650755 | 2720028.3 | 3924415 | 0.324547735 | 0.306895856 |
| FJ31 | 2782043 | 2716147.8 | 3918418 | 0.290008621 | 0.306825408 |
| FJ32 | 2845188 | 2720267 | 3924777 | 0.27507015 | 0.306898965 |
| FJ33 | 2851074 | 2720130.1 | 3924581 | 0.273534168 | 0.306899233 |
| FJ34 | 2814408 | 2719610.5 | 3923812 | 0.282736278 | 0.30689582 |
| FJ35 | 2832279 | 2720091.5 | 3924521 | 0.278312181 | 0.306898472 |
| TW1 | 1420003 | 1419357.8 | 2376860 | 0.402571881 | 0.402843331 |
| TW2 | 1400088 | 1418144.8 | 2374672 | 0.410407837 | 0.402803924 |
| TW3 | 1430822 | 1415041.4 | 2369106 | 0.396049818 | 0.402710812 |
| TW4 | 1551388 | 1410720.1 | 2361480 | 0.343044193 | 0.402611879 |
| TW5 | 1404139 | 1418238 | 2374843 | 0.408744494 | 0.40280768 |
| GD1 | 603840 | 1120619.5 | 2509167 | 0.759346429 | 0.55338983 |
| GD2 | 1331035 | 1120619.5 | 2503281 | 0.468283824 | 0.552339709 |
| GX1 | 1669052 | 1747443.8 | 2778830 | 0.399368799 | 0.371158437 |
| GX2 | 1992363 | 1674026.1 | 2654941 | 0.249564115 | 0.369467683 |
| GX3 | 2080661 | 1598460.3 | 2527463 | 0.176778849 | 0.367563323 |
| GX4 | 1924163 | 1744772 | 2774318 | 0.306437474 | 0.371098771 |
| GX5 | 1773504 | 1741420.2 | 2768637 | 0.359430651 | 0.371018953 |
| GX6 | 1800083 | 1736465.2 | 2760198 | 0.3478428 | 0.370891074 |
| WD1 | 2777892 | 2644615.8 | 3828720 | 0.274459349 | 0.309268946 |
| WD2 | 2791335 | 2634253.4 | 3813448 | 0.268028566 | 0.309220055 |
| WD3 | 2760181 | 2648493.5 | 3834799 | 0.280227986 | 0.30935272 |
| WD4 | 2788017 | 2644317.4 | 3828463 | 0.27176598 | 0.309300521 |
| WD5 | 2784050 | 2645146.9 | 3829888 | 0.273072737 | 0.309340926 |
| WD6 | 2818082 | 2644712.1 | 3829075 | 0.264030608 | 0.309307835 |
| WD7 | 2846201 | 2635551.9 | 3815348 | 0.25401274 | 0.30922372 |
| WD8 | 2775200 | 2645830.7 | 3830677 | 0.275532758 | 0.309304674 |
| WD9 | 2824879 | 2650765.6 | 3838213 | 0.264011924 | 0.309375066 |
| WD10 | 2750904 | 2649645 | 3836653 | 0.282993797 | 0.309386332 |
| WD11 | 2594849 | 2656838.3 | 3847381 | 0.325554449 | 0.309442371 |
| WD12 | 2607081 | 2657377.8 | 3848121 | 0.322505451 | 0.309434968 |
| WD13 | 2644267 | 2655798.3 | 3845801 | 0.312427502 | 0.309429089 |
| WD14 | 2658280 | 2657576.9 | 3848494 | 0.309267469 | 0.309450164 |
| WD15 | 2814413 | 2655964.1 | 3846161 | 0.268253981 | 0.309450618 |
| WD16 | 2814846 | 2655584.5 | 3845655 | 0.268045105 | 0.309458467 |
| WD17 | 3062652 | 2578983.3 | 3727749 | 0.17841786 | 0.308166054 |
| WD18 | 2817203 | 2656013.2 | 3846231 | 0.267541913 | 0.30945042 |
| WD19 | 2817576 | 2656053.8 | 3846282 | 0.267454648 | 0.309449021 |
| V1 | 1766837 | 1261422.2 | 2083458 | 0.151968986 | 0.394553574 |
| V2 | 1773833 | 1269058.3 | 2097687 | 0.154386236 | 0.395020182 |
| V3 | 1522660 | 1429342.5 | 2382013 | 0.360767552 | 0.399943451 |
| V4 | 1770150 | 1288771.7 | 2137593 | 0.171895679 | 0.397092103 |
| V5 | 1567828 | 1437495.8 | 2396377 | 0.345750689 | 0.400137875 |
| C1 | 1748769 | 1239601 | 2034748 | 0.140547626 | 0.390784018 |
| C2 | 1763829 | 1237796.5 | 2031360 | 0.131700437 | 0.39065626 |
| C3 | 1599248 | 1431778.2 | 2377869 | 0.327444868 | 0.39787339 |
| C4 | 1442653 | 1466260.2 | 2438617 | 0.408413457 | 0.398732888 |
| C5 | 1925388 | 1303210 | 2147651 | 0.10349121 | 0.393192842 |
| L1 | 607865 | 324680.8 | 839268 | 0.275720032 | 0.613138116 |
| L2 | 612444 | 324680.8 | 846362 | 0.276380556 | 0.616380698 |
| P1 | 1661726 | 1543014.3 | 2504317 | 0.336455409 | 0.383858234 |
| P2 | 1871465 | 1536174.3 | 2492535 | 0.249172028 | 0.383689978 |
| P3 | 1620722 | 1538276.5 | 2496017 | 0.350676698 | 0.383707523 |
| P4 | 1524573 | 1543084.4 | 2504441 | 0.39125218 | 0.38386075 |
| P5 | 1430370 | 1542690.4 | 2503766 | 0.428712587 | 0.383852005 |
| B1 | 1252568 | 906287.2 | 1662108 | 0.246397948 | 0.454736275 |
| B2 | 1035113 | 636306.4 | 1122427 | 0.07779036 | 0.433097743 |
| B3 | 1159179 | 730782.2 | 1302367 | 0.109944432 | 0.438881513 |
| B4 | 1016457 | 956731.4 | 1778198 | 0.428378055 | 0.461965765 |
