# Supplementary material for: Analysis of whole-genome re-sequencing data of ducks reveals a diverse demographic history and extensive gene flow between Southeast/South Asian and Chinese populations
Source: Genet Sel Evol. 2021 Apr 13;53:35. doi: 10.1186/s12711-021-00627-0 (PMC8042899; doi:10.1186/s12711-021-00627-0)
Supplement: Supplementary file 11 — Additional file 11: Table S5. The D-statistics detect whether there was gene flow between Southeast Asia and South Asian populations. [file 12711_2021_627_MOESM11_ESM.docx]

Table S5. D statistics detect whether there was gene flow between Southeast Asia and South Asian populations

| **P1** | **P2** | **P3** | **P4** | **AverageD** | **sdD** | **ZD** |
| --- | --- | --- | --- | --- | --- | --- |
| Pakistan | Bangladesh | Vietnam | Outgroup | 0.018200394 | 0.004370147 | 4.164709757 |
| Pakistan | Bangladesh | Cambodia | Outgroup | 0.024734344 | 0.004290477 | 5.764940282 |
| Pakistan | Bangladesh | Laos | Outgroup | 0.064541096 | 0.004894646 | 13.18606131 |
