# Supplementary material for: Analysis of whole-genome re-sequencing data of ducks reveals a diverse demographic history and extensive gene flow between Southeast/South Asian and Chinese populations
Source: Genet Sel Evol. 2021 Apr 13;53:35. doi: 10.1186/s12711-021-00627-0 (PMC8042899; doi:10.1186/s12711-021-00627-0)
Supplement: Supplementary file 14 — Additional file 14: Table S6. Eighteen extreme fd peaks (fd > = 0.5) with the introgressed genomic regions between Guangxi and Southeast/South Asian populations using the modified f-statistic (fd). Introgressed genomic regions were not shorter than 200 kb. [file 12711_2021_627_MOESM14_ESM.docx]

Table S6. Eighteen extreme *fd* peaks (*fd* >= 0.5) with the introgressed genomic regions between Guangxi and Southeast/South Asian populations using the modified *f*-statistic (*fd*)

|  | scaffold | start | end | dxy | Fst | fd | gene |
| --- | --- | --- | --- | --- | --- | --- | --- |
| 1 | 1 | 85360001 | 85460000 | 0.2021 | 0 | 0.5314 | DMD |
|  | 1 | 85380001 | 85480000 | 0.2035 | 0 | 0.5354 | DMD |
|  | 1 | 85400001 | 85500000 | 0.2078 | 0 | 0.5664 | DMD |
|  | 1 | 85420001 | 85520000 | 0.2342 | 0 | 0.5314 | DMD |
|  | 1 | 85440001 | 85540000 | 0.2334 | 0 | 0.5255 | DMD |
|  | 1 | 85460001 | 85560000 | 0.2502 | 0 | 0.5212 | DMD |
|  | 1 | 85480001 | 85580000 | 0.2621 | 0 | 0.5352 | DMD |
|  | 1 | 85900001 | 86000000 | 0.1138 | 0.0098 | 0.5568 | DMD |
|  | 1 | 85920001 | 86020000 | 0.0842 | 0.0136 | 0.619 | DMD |
|  | 1 | 85940001 | 86040000 | 0.0641 | 0.0165 | 0.6551 | DMD |
|  | 1 | 85960001 | 86060000 | 0.06 | 0.0075 | 0.6551 | DMD |
|  | 1 | 85980001 | 86080000 | 0.0783 | 0 | 0.6258 | DMD, LOC110353214 |
|  | 1 | 86000001 | 86100000 | 0.1215 | 0 | 0.5794 | DMD, LOC110353214 |
| 2 | 1 | 104240001 | 104340000 | 0.1494 | 0.011 | 0.5344 | LOC106019573 |
|  | 1 | 104260001 | 104360000 | 0.1341 | 0 | 0.6075 | LOC106019573 |
|  | 1 | 104280001 | 104380000 | 0.1381 | 0 | 0.5755 | LOC106019573 |
|  | 1 | 104300001 | 104400000 | 0.1465 | 0 | 0.5732 | LOC106019573, LOC113844489 |
|  | 1 | 104320001 | 104420000 | 0.1665 | 0 | 0.5492 | LOC106019573, LOC113844489 |
|  | 1 | 104340001 | 104440000 | 0.1729 | 0 | 0.5204 | LOC106019573, LOC113844489 |
|  | 1 | 104360001 | 104460000 | 0.1767 | 0 | 0.5082 | LOC106019573, LOC113844489 |
| 3 | 1 | 140800001 | 140900000 | 0.1282 | 0.0017 | 0.5435 | RESF1, LOC113845520,BICD1 |
|  | 1 | 140820001 | 140920000 | 0.1239 | 0.0053 | 0.5523 | RESF1, LOC113845520,BICD1 |
|  | 1 | 140840001 | 140940000 | 0.1296 | 0.007 | 0.5074 | RESF1, LOC113845520,BICD1 |
|  | 1 | 140860001 | 140960000 | 0.1271 | 0.01 | 0.5191 | BICD1 |
|  | 1 | 140880001 | 140980000 | 0.1373 | 0.01 | 0.5257 | BICD1 |
|  | 1 | 140900001 | 141000000 | 0.1363 | 0.0076 | 0.5223 | BICD1 |
|  | 1 | 140920001 | 141020000 | 0.1433 | 0.0062 | 0.5009 | BICD1 |
|  | 1 | 140940001 | 141040000 | 0.1457 | 0.003 | 0.5059 | BICD1 |
|  | 1 | 140960001 | 141060000 | 0.1558 | 0 | 0.5209 | BICD1 |
|  | 1 | 140980001 | 141080000 | 0.16 | 0 | 0.5217 | BICD1,FGD4 |
|  | 1 | 141000001 | 141100000 | 0.175 | 0 | 0.527 | BICD1,FGD4 |
|  | 1 | 141020001 | 141120000 | 0.1781 | 0 | 0.5436 | BICD1,FGD4 |
|  | 1 | 141040001 | 141140000 | 0.1796 | 0 | 0.5427 | BICD1,FGD4 |
|  | 1 | 141060001 | 141160000 | 0.1817 | 0.0004 | 0.509 | BICD1,FGD4 |
| 4 | 1 | 172940001 | 173040000 | 0.0645 | 0.0113 | 0.5246 | LOC113843620，LOC113843622，PNPLA8，LOC101796234 |
|  | 1 | 172960001 | 173060000 | 0.0543 | 0.0118 | 0.6323 | LOC113843620，LOC113843622，PNPLA8，LOC101796234 |
|  | 1 | 172980001 | 173080000 | 0.0379 | 0.0119 | 0.8227 | PNPLA8，LOC101796234 |
|  | 1 | 173000001 | 173100000 | 0.0385 | 0.0089 | 0.8398 | PNPLA8，LOC101796234，THAP5 |
|  | 1 | 173020001 | 173120000 | 0.0533 | 0.0033 | 0.7082 | PNPLA8，LOC101796234，THAP5，DNAJB9 |
|  | 1 | 173040001 | 173140000 | 0.0677 | 0.0012 | 0.5936 | LOC101796234，THAP5，DNAJB9 |
|  | 1 | 173060001 | 173160000 | 0.076 | 0 | 0.5416 | THAP5，DNAJB9 |
|  | 1 | 173140001 | 173240000 | 0.0527 | 0 | 0.5691 |  |
|  | 1 | 173160001 | 173260000 | 0.034 | 0 | 0.6741 |  |
|  | 1 | 173180001 | 173280000 | 0.0193 | 0 | 0.7972 |  |
|  | 1 | 173200001 | 173300000 | 0.0196 | 0 | 0.7998 |  |
|  | 1 | 173220001 | 173320000 | 0.0195 | 0 | 0.8748 |  |
|  | 1 | 173240001 | 173340000 | 0.0199 | 0.0007 | 0.865 |  |
|  | 1 | 173260001 | 173360000 | 0.0208 | 0.0013 | 0.8766 |  |
|  | 1 | 173280001 | 173380000 | 0.0218 | 0.0008 | 0.8744 |  |
|  | 1 | 173300001 | 173400000 | 0.0211 | 0.0011 | 0.8577 |  |
|  | 1 | 173320001 | 173420000 | 0.02 | 0.001 | 0.8609 |  |
|  | 1 | 173340001 | 173440000 | 0.0213 | 0.0005 | 0.8747 |  |
|  | 1 | 173360001 | 173460000 | 0.0215 | 0.0004 | 0.8855 |  |
|  | 1 | 173380001 | 173480000 | 0.0195 | 0.0006 | 0.8793 |  |
|  | 1 | 173400001 | 173500000 | 0.0201 | 0.0017 | 0.8739 |  |
|  | 1 | 173420001 | 173520000 | 0.0253 | 0 | 0.8679 |  |
|  | 1 | 173440001 | 173540000 | 0.0228 | 0 | 0.8719 | LOC106015046 |
|  | 1 | 173460001 | 173560000 | 0.0228 | 0 | 0.8674 | LOC106015046，LOC106015047 |
|  | 1 | 173480001 | 173580000 | 0.0241 | 0 | 0.8743 | LOC106015046，LOC106015047 |
|  | 1 | 173500001 | 173600000 | 0.0242 | 0 | 0.8928 | LOC106015046，LOC106015047 |
|  | 1 | 173520001 | 173620000 | 0.0189 | 0 | 0.8987 | LOC106015046，LOC106015047 |
|  | 1 | 173540001 | 173640000 | 0.021 | 0 | 0.8928 | LOC106015047 |
|  | 1 | 173560001 | 173660000 | 0.0218 | 0.0013 | 0.8752 | LOC106015047 |
|  | 1 | 173580001 | 173680000 | 0.0217 | 0.0017 | 0.8752 |  |
|  | 1 | 173600001 | 173700000 | 0.0204 | 0.002 | 0.8194 |  |
|  | 1 | 173620001 | 173720000 | 0.0206 | 0.0021 | 0.7723 |  |
|  | 1 | 173640001 | 173740000 | 0.0194 | 0.0014 | 0.7479 |  |
|  | 1 | 173660001 | 173760000 | 0.018 | 0 | 0.6932 |  |
|  | 1 | 173680001 | 173780000 | 0.0292 | 0 | 0.5921 |  |
|  | 1 | 173700001 | 173800000 | 0.0417 | 0 | 0.5777 |  |
|  | 1 | 173720001 | 173820000 | 0.0635 | 0 | 0.5285 |  |
| 5 | 2 | 15360001 | 15460000 | 0.1299 | 0.0143 | 0.5289 | LOC101790111，ZNF438，ADGRA1，LOC110351639 |
|  | 2 | 15380001 | 15480000 | 0.1069 | 0.0225 | 0.5745 | LOC101790111，LOC101789559，ADGRA1，LOC110351639，KNDC1，LOC110351640 |
|  | 2 | 15400001 | 15500000 | 0.0908 | 0.0232 | 0.6296 | ZNF438，LOC101789559，LOC110354173，SVIL |
|  | 2 | 15420001 | 15520000 | 0.095 | 0.0148 | 0.6444 | ZNF438，LOC101789559，LOC110354173，SVIL |
|  | 2 | 15440001 | 15540000 | 0.1021 | 0.0073 | 0.6406 | ZNF438，LOC101789559，LOC110354173，SVIL |
|  | 2 | 15460001 | 15560000 | 0.1092 | 0.0037 | 0.6296 | LOC101789559，LOC110354173，SVIL |
|  | 2 | 15480001 | 15580000 | 0.1146 | 0 | 0.6136 | LOC110354173，SVIL |
|  | 2 | 15500001 | 15600000 | 0.1249 | 0.007 | 0.554 | SVIL |
| 6 | 2 | 29680001 | 29780000 | 0.2829 | 0.0224 | 0.5985 | DGKB |
|  | 2 | 29700001 | 29800000 | 0.2581 | 0.0123 | 0.6099 | DGKB |
|  | 2 | 29720001 | 29820000 | 0.2541 | 0.0023 | 0.6052 | DGKB |
|  | 2 | 29740001 | 29840000 | 0.2492 | 0 | 0.5639 | DGKB |
|  | 2 | 29760001 | 29860000 | 0.2831 | 0 | 0.5787 | DGKB |
|  | 2 | 29780001 | 29880000 | 0.2868 | 0 | 0.545 |  |
|  | 2 | 29800001 | 29900000 | 0.296 | 0 | 0.5488 |  |
|  | 2 | 29820001 | 29920000 | 0.2998 | 0 | 0.5664 | AGMO |
|  | 2 | 29840001 | 29940000 | 0.3054 | 0 | 0.6612 | AGMO |
|  | 2 | 29880001 | 29980000 | 0.2434 | 0 | 0.6472 | AGMO |
|  | 2 | 29900001 | 30000000 | 0.2894 | 0 | 0.5901 | AGMO |
|  | 2 | 29920001 | 30020000 | 0.2787 | 0 | 0.5554 | AGMO |
|  | 2 | 29940001 | 30040000 | 0.277 | 0 | 0.5229 | AGMO |
|  | 2 | 29960001 | 30060000 | 0.2833 | 0 | 0.5362 | AGMO |
|  | 2 | 29980001 | 30080000 | 0.3041 | 0 | 0.5331 | AGMO |
|  | 2 | 30000001 | 30100000 | 0.2817 | 0 | 0.5321 | AGMO |
| 7 | 2 | 99380001 | 99480000 | 0.3336 | 0 | 0.5005 | LOC106018653 |
|  | 2 | 99400001 | 99500000 | 0.3314 | 0 | 0.6208 | LOC106018653 |
|  | 2 | 99420001 | 99520000 | 0.3294 | 0 | 0.5675 | LOC106018653 |
|  | 2 | 99440001 | 99540000 | 0.344 | 0 | 0.5596 | LOC106018653，DSEL |
|  | 2 | 99460001 | 99560000 | 0.2859 | 0.0007 | 0.5612 | LOC106018653，DSEL |
|  | 2 | 99480001 | 99580000 | 0.2756 | 0.0081 | 0.532 | LOC106018653，DSEL |
| 8 | 2 | 145400001 | 145500000 | 0.0995 | 0.0124 | 0.6575 |  |
|  | 2 | 145420001 | 145520000 | 0.0748 | 0.0133 | 0.7423 |  |
|  | 2 | 145440001 | 145540000 | 0.0696 | 0.0148 | 0.7971 |  |
|  | 2 | 145460001 | 145560000 | 0.0706 | 0.0157 | 0.7963 |  |
|  | 2 | 145480001 | 145580000 | 0.1062 | 0.0177 | 0.5936 | FAM84B |
|  | 2 | 149580001 | 149680000 | 0.1184 | 0.0441 | 0.5294 |  |
|  | 2 | 149600001 | 149700000 | 0.0987 | 0.0221 | 0.5706 | LOC106019374 |
|  | 2 | 149620001 | 149720000 | 0.0929 | 0.0121 | 0.5772 | LOC106019374，LOC113842930 |
|  | 2 | 149640001 | 149740000 | 0.0902 | 0.0013 | 0.5826 | LOC106019374，LOC113842930 |
|  | 2 | 149660001 | 149760000 | 0.0872 | 0 | 0.5591 | LOC106019374，LOC113842930 |
|  | 2 | 149680001 | 149780000 | 0.0842 | 0 | 0.5623 | LOC106019374，LOC113842930 |
|  | 2 | 149700001 | 149800000 | 0.0843 | 0 | 0.5826 | LOC113842930 |
|  | 2 | 149720001 | 149820000 | 0.0818 | 0 | 0.6069 | LOC113842930 |
|  | 2 | 149740001 | 149840000 | 0.087 | 0 | 0.5526 | LOC113842930，LOC113842931 |
| 9 | 3 | 60560001 | 60660000 | 0.0484 | 0.016 | 0.7246 | AKAP7，TRNAQ-UUG，EPB41L2 |
|  | 3 | 60580001 | 60680000 | 0.0422 | 0.0122 | 0.7699 | AKAP7，EPB41L2 |
|  | 3 | 60600001 | 60700000 | 0.0375 | 0.008 | 0.7781 | EPB41L2 |
|  | 3 | 60620001 | 60720000 | 0.0435 | 0.0094 | 0.7692 | EPB41L2 |
|  | 3 | 60640001 | 60740000 | 0.0468 | 0.0124 | 0.7761 | EPB41L2，SMLR1 |
|  | 3 | 60660001 | 60760000 | 0.0455 | 0.013 | 0.7585 | EPB41L2，SMLR1 |
|  | 3 | 60680001 | 60780000 | 0.0464 | 0.0122 | 0.6988 | EPB41L2，SMLR1 |
|  | 3 | 60700001 | 60800000 | 0.0611 | 0.0248 | 0.6277 | EPB41L2，SMLR1 |
|  | 3 | 60720001 | 60820000 | 0.0639 | 0.0266 | 0.5963 | EPB41L2，SMLR1 |
|  | 3 | 60740001 | 60840000 | 0.0711 | 0.0282 | 0.5941 |  |
|  | 3 | 60760001 | 60860000 | 0.0834 | 0.0293 | 0.564 |  |
|  | 3 | 60780001 | 60880000 | 0.1197 | 0.0396 | 0.526 | LOC113843232 |
| 10 | 3 | 107260001 | 107360000 | 0.1357 | 0.0018 | 0.5086 | LOC110351866 |
|  | 3 | 107280001 | 107380000 | 0.1232 | 0.0025 | 0.5346 | LOC110351866, LOC101802235,LOC106015549 |
|  | 3 | 107300001 | 107400000 | 0.1048 | 0.0053 | 0.5429 | LOC110351866, LOC101802235,LOC106015549 |
|  | 3 | 107320001 | 107420000 | 0.0796 | 0.0133 | 0.5662 | LOC101802235, LOC106015549 |
|  | 3 | 107340001 | 107440000 | 0.0827 | 0.0189 | 0.5675 | LOC101802235, LOC106015549 |
|  | 3 | 107360001 | 107460000 | 0.0875 | 0.0247 | 0.5586 | LOC101802235, LOC106015549,OSR1 |
| 11 | 5 | 16660001 | 16760000 | 0.1261 | 0.0119 | 0.5609 | EVL |
|  | 5 | 16680001 | 16780000 | 0.1001 | 0.0153 | 0.6025 | EVL，EML1 |
|  | 5 | 16700001 | 16800000 | 0.0984 | 0.0239 | 0.5799 | EVL，EML1 |
|  | 5 | 16720001 | 16820000 | 0.0975 | 0.0246 | 0.589 | EVL，EML1，LOC113843686 |
|  | 5 | 16740001 | 16840000 | 0.0995 | 0.0257 | 0.5762 | EVL，EML1，LOC113843686 |
|  | 5 | 16760001 | 16860000 | 0.1031 | 0.0268 | 0.561 | EML1，LOC113843686 |
|  | 5 | 16780001 | 16880000 | 0.0933 | 0.0243 | 0.5451 | EML1，LOC113843686 |
|  | 5 | 16800001 | 16900000 | 0.0771 | 0.0202 | 0.5539 | EML1，LOC113843686，LOC106017479 |
|  | 5 | 16820001 | 16920000 | 0.0652 | 0.0186 | 0.5441 | EML1，LOC106017479 |
|  | 5 | 16840001 | 16940000 | 0.0596 | 0.0167 | 0.6163 | EML1，LOC106017479，LOC101792124，LOC101792317 |
|  | 5 | 16860001 | 16960000 | 0.0545 | 0.0147 | 0.7606 | LOC106017479，LOC101792124，LOC101792317，HHIPL1 |
| 12 | 5 | 46520001 | 46620000 | 0.1244 | 0.0048 | 0.5264 | NOVA1，LOC113843837 |
|  | 5 | 46540001 | 46640000 | 0.083 | 0.0156 | 0.6678 | NOVA1，LOC113843837 |
|  | 5 | 46560001 | 46660000 | 0.0555 | 0.0167 | 0.7073 | LOC113843837 |
|  | 5 | 46580001 | 46680000 | 0.0584 | 0.0156 | 0.6924 | LOC113843837 |
|  | 5 | 46600001 | 46700000 | 0.0567 | 0.0192 | 0.6567 |  |
|  | 5 | 46620001 | 46720000 | 0.0684 | 0.0197 | 0.6872 |  |
|  | 5 | 46640001 | 46740000 | 0.0775 | 0.0202 | 0.7157 |  |
|  | 5 | 46660001 | 46760000 | 0.0872 | 0.0221 | 0.7381 |  |
|  | 5 | 46680001 | 46780000 | 0.0904 | 0.0233 | 0.7603 |  |
|  | 5 | 46700001 | 46800000 | 0.0954 | 0.0232 | 0.7674 |  |
|  | 5 | 46720001 | 46820000 | 0.095 | 0.0226 | 0.7884 |  |
|  | 5 | 46740001 | 46840000 | 0.0856 | 0.0214 | 0.7905 |  |
|  | 5 | 46760001 | 46860000 | 0.0822 | 0.0209 | 0.7989 |  |
|  | 5 | 46780001 | 46880000 | 0.0741 | 0.0217 | 0.7434 |  |
|  | 5 | 47480001 | 47580000 | 0.2052 | 0 | 0.5042 | LOC113843870，PRKD1 |
| 13 | 6 | 15060001 | 15160000 | 0.2041 | 0.0136 | 0.5068 | LOC101803823，ADGRA1 |
|  | 6 | 15080001 | 15180000 | 0.1967 | 0.0084 | 0.5177 | LOC101803823，ADGRA1 |
|  | 6 | 15100001 | 15200000 | 0.1941 | 0.0015 | 0.5671 | ADGRA1 |
|  | 6 | 15120001 | 15220000 | 0.1718 | 0 | 0.6402 | ADGRA1 |
|  | 6 | 15140001 | 15240000 | 0.1645 | 0 | 0.703 | ADGRA1 |
|  | 6 | 15160001 | 15260000 | 0.176 | 0 | 0.6634 | ADGRA1 |
|  | 6 | 15180001 | 15280000 | 0.1786 | 0 | 0.5933 | ADGRA1 |
|  | 6 | 15200001 | 15300000 | 0.1945 | 0 | 0.5387 | ADGRA1 |
|  | 6 | 15220001 | 15320000 | 0.2356 | 0 | 0.5368 | ADGRA1 |
|  | 6 | 15240001 | 15340000 | 0.2406 | 0 | 0.5605 | ADGRA1 |
|  | 6 | 15260001 | 15360000 | 0.2355 | 0 | 0.5529 | ADGRA1 |
|  | 6 | 15280001 | 15380000 | 0.2341 | 0 | 0.5958 | ADGRA1 |
|  | 6 | 15300001 | 15400000 | 0.228 | 0 | 0.5964 | ADGRA1 |
|  | 6 | 15320001 | 15420000 | 0.2233 | 0 | 0.594 | ADGRA1 |
|  | 6 | 15340001 | 15440000 | 0.2046 | 0 | 0.5794 | ADGRA1，LOC110351639 |
|  | 6 | 15360001 | 15460000 | 0.2265 | 0 | 0.5807 | LOC101790111，ZNF438，ADGRA1，LOC110351639 |
|  | 6 | 15380001 | 15480000 | 0.2434 | 0 | 0.5144 | LOC101790111，LOC101789559，ADGRA1，LOC110351639，KNDC1，LOC110351640 |
| 14 | 6 | 16980001 | 17080000 | 0.3305 | 0 | 0.5086 | PLCE1 |
|  | 6 | 17000001 | 17100000 | 0.2744 | 0 | 0.5892 | PLCE1，TBC1D12，NOC3L |
|  | 6 | 17020001 | 17120000 | 0.2629 | 0 | 0.6109 | PLCE1，TBC1D12，NOC3L |
|  | 6 | 17040001 | 17140000 | 0.2304 | 0 | 0.6837 | PLCE1，TBC1D12，NOC3L |
|  | 6 | 17060001 | 17160000 | 0.2504 | 0 | 0.5742 | PLCE1，TBC1D12，NOC3L，LOC101799912 |
|  | 6 | 17080001 | 17180000 | 0.26 | 0 | 0.581 | NOC3L，TBC1D12，LOC101799912，FGFBP3 |
|  | 6 | 17120001 | 17220000 | 0.2966 | 0 | 0.5057 | TBC1D12，LOC101799912，FGFBP3，TNKS2 |
| 15 | 7 | 23740001 | 23840000 | 0.2347 | 0 | 0.5299 | FASTKD1，KLHL41，BBS5，LOC113844119，LOC101804634，LRP2，LAMC1，LOC110352757，NPL，HEBP2 |
|  | 7 | 23760001 | 23860000 | 0.2418 | 0 | 0.5366 | BBS5，LOC101804634，LRP2，LAMC1，LOC110352757，NPL，HEBP2，LOC113844412，LOC113844403，LOC113844420，LOC113844414，LOC113844416，LOC113844415，LOC113844421，LOC113844402，LOC113844413，ZBTB37 |
|  | 7 | 23780001 | 23880000 | 0.2387 | 0 | 0.5584 | LOC101804634，LRP2，LAMC1，NPL，HEBP2，LOC113844412，LOC113844403，LOC113844420，LOC113844414，LOC113844416，LOC113844415，LOC113844402，LOC113844413，LOC113844421，ZBTB37，SERPINC1 |
|  | 7 | 23800001 | 23900000 | 0.2243 | 0 | 0.5488 | LOC101804634，LRP2，NPL，HEBP2，LOC113844412，LOC113844403，LOC113844420，LOC113844414，LOC113844416，LOC113844415，LOC113844402，LOC113844421，LOC113844413，ZBTB37，SERPINC1，RC3H1 |
|  | 7 | 23820001 | 23920000 | 0.2421 | 0 | 0.5829 | LRP2，NPL，HEBP2，LOC113844412，LOC113844403，LOC113844420，LOC113844414，LOC113844416，LOC113844415，LOC113844402，LOC113844421，LOC113844413，ZBTB37，SERPINC1，RC3H1 |
|  | 7 | 23840001 | 23940000 | 0.2438 | 0 | 0.5254 | LRP2，NPL，HEBP2，LOC113844412，LOC113844403，LOC113844420，LOC113844414，LOC113844416，LOC113844415，LOC113844402，LOC113844421，LOC113844413，ZBTB37，SERPINC1，RC3H1 |
| 16 | 8 | 23600001 | 23700000 | 0.1244 | 0 | 0.5592 | ARPC5，NCF2，SMG7，NMNAT2，LAMC2 |
|  | 8 | 23620001 | 23720000 | 0.1201 | 0 | 0.577 | SMG7，NMNAT2，LAMC2，LAMC1 |
|  | 8 | 23640001 | 23740000 | 0.1272 | 0 | 0.6069 | SMG7，NMNAT2，LAMC2，LAMC1 |
|  | 8 | 23660001 | 23760000 | 0.1442 | 0 | 0.5536 | SMG7，NMNAT2，LAMC2，LAMC1，LOC110352757 |
|  | 8 | 23680001 | 23780000 | 0.1467 | 0 | 0.5446 | NMNAT2，LAMC2，LAMC1，LOC110352757 |
|  | 8 | 23700001 | 23800000 | 0.1312 | 0 | 0.5384 | LAMC2，LAMC1，LOC110352757，NPL |
|  | 8 | 23720001 | 23820000 | 0.1108 | 0 | 0.5247 | LAMC1，LOC110352757，NPL |
|  | 8 | 23740001 | 23840000 | 0.1018 | 0 | 0.5144 | FASTKD1，KLHL41，BBS5，LOC113844119，LOC101804634，LRP2，LAMC1，LOC110352757，NPL，HEBP2 |
|  | 8 | 23760001 | 23860000 | 0.0913 | 0 | 0.5288 | BBS5，LOC101804634，LRP2，LAMC1，LOC110352757，NPL，HEBP2，LOC113844412，LOC113844403，LOC113844420，LOC113844414，LOC113844416，LOC113844415，LOC113844421，LOC113844402，LOC113844413，ZBTB37 |
|  | 8 | 23780001 | 23880000 | 0.0856 | 0 | 0.5287 | LOC101804634，LRP2，LAMC1，NPL，HEBP2，LOC113844412，LOC113844403，LOC113844420，LOC113844414，LOC113844416，LOC113844415，LOC113844402，LOC113844413，LOC113844421，ZBTB37，SERPINC1 |
|  | 8 | 23800001 | 23900000 | 0.0891 | 0 | 0.5044 | LOC101804634，LRP2，NPL，HEBP2，LOC113844412，LOC113844403，LOC113844420，LOC113844414，LOC113844416，LOC113844415，LOC113844402，LOC113844421，LOC113844413，ZBTB37，SERPINC1，RC3H1 |
|  | 8 | 23820001 | 23920000 | 0.1044 | 0 | 0.5519 | LRP2，NPL，HEBP2，LOC113844412，LOC113844403，LOC113844420，LOC113844414，LOC113844416，LOC113844415，LOC113844402，LOC113844421，LOC113844413，ZBTB37，SERPINC1，RC3H1 |
|  | 8 | 23840001 | 23940000 | 0.1182 | 0 | 0.5535 | LRP2，NPL，HEBP2，LOC113844412，LOC113844403，LOC113844420，LOC113844414，LOC113844416，LOC113844415，LOC113844402，LOC113844421，LOC113844413，ZBTB37，SERPINC1，RC3H1 |
|  | 8 | 23860001 | 23960000 | 0.1547 | 0 | 0.5187 | ZBTB37，SERPINC1，RC3H1，LOC113844358 |
| 17 | 12 | 6980001 | 7080000 | 0.1083 | 0.0309 | 0.5148 | CDH13，PISD，SFI1，EIF4ENIF1，DRG1，PATZ1 |
|  | 12 | 7000001 | 7100000 | 0.1096 | 0.0293 | 0.5126 | CDH13，LOC106017289，SFI1，EIF4ENIF1，DRG1，PATZ1，PIK3IP1，LIMK2 |
|  | 12 | 7100001 | 7200000 | 0.0766 | 0.0237 | 0.5066 | LOC106017289，MPHOSPH6，LOC101791654 |
|  | 12 | 7120001 | 7220000 | 0.0693 | 0.0239 | 0.6012 | LOC106017289，MPHOSPH6，LOC101791654，LOC101791850，LOC101791461 |
|  | 12 | 7140001 | 7240000 | 0.0524 | 0.0126 | 0.6949 | LOC106017289，MPHOSPH6，LOC101791654，LOC101791850，LOC101791461 |
|  | 12 | 7160001 | 7260000 | 0.0472 | 0.0111 | 0.6897 | MPHOSPH6，LOC101791654，LOC101791850，LOC101791461，HSD17B2 |
|  | 12 | 7180001 | 7280000 | 0.0485 | 0.0106 | 0.6818 | MPHOSPH6，LOC101791654，LOC101791850，LOC101791461，HSD17B2，LOC106017288，SDR42E1 |
|  | 12 | 7200001 | 7300000 | 0.0448 | 0.0079 | 0.6728 | LOC101791654，LOC101791850，LOC101791461，HSD17B2，LOC106017288，SDR42E1，PLCG2 |
| 18 | 16 | 6860001 | 6960000 | 0.1472 | 0 | 0.5087 | YWHAH，ANHX，DEPDC5，PRR14L，LOC101796956 |
|  | 16 | 6880001 | 6980000 | 0.1311 | 0 | 0.5723 | ANHX，DEPDC5，PRR14L，LOC101796956，PISD |
|  | 16 | 6900001 | 7000000 | 0.1188 | 0 | 0.5633 | DEPDC5，PRR14L，LOC101796956，PISD，SFI1 |
|  | 16 | 6920001 | 7020000 | 0.123 | 0 | 0.5658 | DEPDC5，PRR14L，LOC101796956，PISD，SFI1 |
|  | 16 | 6940001 | 7040000 | 0.1321 | 0 | 0.5425 | PRR14L，LOC101796956，PISD，SFI1，EIF4ENIF1 |
|  | 16 | 6960001 | 7060000 | 0.1263 | 0 | 0.5585 | LOC101796956，PISD，SFI1，EIF4ENIF1，DRG1 |
|  | 16 | 6980001 | 7080000 | 0.1254 | 0 | 0.5411 | CDH13，PISD，SFI1，EIF4ENIF1，DRG1，PATZ1 |
|  | 16 | 7000001 | 7100000 | 0.1074 | 0 | 0.5377 | CDH13，LOC106017289，SFI1，EIF4ENIF1，DRG1，PATZ1，PIK3IP1，LIMK2 |
|  | 19 | 7880001 | 7980000 | 0.1491 | 0.0168 | 0.5323 | TOM1L1，LOC101790138，STXBP4，LOC110352057 |
|  | 19 | 7900001 | 8000000 | 0.1464 | 0.0292 | 0.5184 | TOM1L1，LOC101790138，STXBP4，LOC110352057 |
|  | 19 | 7920001 | 8020000 | 0.1423 | 0.0437 | 0.5668 | TOM1L1，LOC101790138，STXBP4，LOC110352057 |
|  | 19 | 7940001 | 8040000 | 0.1337 | 0.0423 | 0.5545 | TOM1L1，LOC101790138，STXBP4，LOC110352057，LOC106015891，HLF |
|  | 19 | 7960001 | 8060000 | 0.1366 | 0.0462 | 0.5366 | STXBP4，LOC110352057，LOC106015891，HLF |

The introgressed genomic regions were not less than 200 kb
