# Supplementary material for: Analysis of whole-genome re-sequencing data of ducks reveals a diverse demographic history and extensive gene flow between Southeast/South Asian and Chinese populations
Source: Genet Sel Evol. 2021 Apr 13;53:35. doi: 10.1186/s12711-021-00627-0 (PMC8042899; doi:10.1186/s12711-021-00627-0)
Supplement: Supplementary file 15 — Additional file 15: Table S7. Functional gene categories enriched for the genes within the introgressed genomic regions. [file 12711_2021_627_MOESM15_ESM.docx]

Table S7. Functional gene categories enriched for the genes with the introgressed genomic regions

| Term | Gene number | P-Value | Gene name |
| --- | --- | --- | --- |
| Phosphatidylinositol signaling system | 3 | 0.001922 | DGKB\|PLCG2\|PLCE1 |
| Inositol phosphate metabolism | 2 | 0.015484 | PLCG2\|PLCE1 |
| ECM-receptor interaction | 2 | 0.022201 | LAMC2\|LAMC1 |
| Glycerophospholipid metabolism | 2 | 0.026711 | PISD\|DGKB |
| AGE-RAGE signaling pathway in diabetic complications | 2 | 0.027759 | PLCG2\|PLCE1 |
| Metabolic pathways | 7 | 0.045768 | NPL\|PISD\|PLCG2\|PLCE1\|DGKB\|HSD17B2\|NMNAT2 |
| Nicotinate and nicotinamide metabolism | 1 | 0.069667 | NMNAT2 |
| Calcium signaling pathway | 2 | 0.081183 | PLCG2\|PLCE1 |
| Regulation of actin cytoskeleton | 2 | 0.081969 | LIMK2\|ARPC5 |
| Steroid hormone biosynthesis | 1 | 0.089376 | HSD17B2 |
| Focal adhesion | 2 | 0.091579 | LAMC2\|LAMC1 |
| Hedgehog signaling pathway | 1 | 0.103887 | LRP2 |
| Amino sugar and nucleotide sugar metabolism | 1 | 0.106283 | NPL |
| Glycerolipid metabolism | 1 | 0.146069 | DGKB |
| mRNA surveillance pathway | 1 | 0.148354 | SMG7 |
| VEGF signaling pathway | 1 | 0.148354 | PLCG2 |
| RNA degradation | 1 | 0.168653 | MPHOSPH6 |
| Salmonella infection | 1 | 0.170879 | ARPC5 |
| ErbB signaling pathway | 1 | 0.186296 | PLCG2 |
| C-type lectin receptor signaling pathway | 1 | 0.205704 | PLCG2 |
| Oocyte meiosis | 1 | 0.224656 | YWHAH |
| Cell cycle | 1 | 0.257256 | YWHAH |
| Phagosome | 1 | 0.26913 | NCF2 |
| mTOR signaling pathway | 1 | 0.298005 | DEPDC5 |
| Endocytosis | 1 | 0.426362 | ARPC5 |
