# Supplementary material for: Analysis of whole-genome re-sequencing data of ducks reveals a diverse demographic history and extensive gene flow between Southeast/South Asian and Chinese populations
Source: Genet Sel Evol. 2021 Apr 13;53:35. doi: 10.1186/s12711-021-00627-0 (PMC8042899; doi:10.1186/s12711-021-00627-0)
Supplement: Supplementary file 23 — Additional file 23: Table S8. Functional gene categories enriched for the 1048 genes with strong selective sweep signals in domestic ducks. [file 12711_2021_627_MOESM23_ESM.docx]

Table S8. Functional gene categories enriched for the 1,048 genes with strong selective sweep signals for domestic ducks

| **Term** | **Gene number** | **P value** | **Gene name** |
| --- | --- | --- | --- |
| Steroid hormone biosynthesis | 6 | 0.001944491 | LOC101803865\|SRD5A2\|HSD17B2\|LOC101791068\|STS\|LOC101797494 |
| Caffeine metabolism | 3 | 0.002293929 | LOC101791850\|LOC101791461\|LOC101791654 |
| Metabolic pathways | 59 | 0.003121139 | LOC101799286\|CTPS2\|LOC101791461\|PTGS2\|B3GALT1\|GBE1\|ACSS2\|NMNAT2\|COQ2\|LOC101799098\|SPTLC3\|RGN\|QARS\|GALNTL6\|PIGC\|ENPP1\|NDUFA9\|BTD\|LOC101791654\|LOC101803865\|PFKP\|PLCG2\|PLCE1\|SMS\|HPSE\|LOC101791850\|LOC101794647\|HSD17B2\|MAN1A2\|PPCS\|LOC101794817\|GPAM\|POLR2C\|FAH\|PCK1\|DEGS1\|ENPP3\|PLA2G4A\|ALG5\|BCO1\|GCNT2\|LOC101798644\|PEMT\|NANP\|LOC101791068\|IDO2\|SPAM1\|DEGS2\|NDUFA5\|RPIA\|ATP6AP1\|GALNT7\|GLUD1\|LOC101797037\|SUCLG1\|MTHFD1L\|NT5M\|LAMA5\|ACSL1 |
| Focal adhesion | 16 | 0.003262005 | EGFR\|LOC101795371\|CCND2\|LAMC2\|HRAS\|LOC101799042\|IGF1R\|LOC101795759\|LAMC1\|RAF1\|ITGA4\|PIK3CB\|RELN\|LAMA5\|CRKL\|MAPK8 |
| ErbB signaling pathway | 9 | 0.003843949 | EGFR\|PLCG2\|ERBB4\|HRAS\|LOC101795759\|RAF1\|PIK3CB\|CRKL\|MAPK8 |
| ABC transporters | 6 | 0.005339383 | ABCG5\|ABCC4\|ABCA2\|ABCG8\|ABCG2\|ABCD2 |
| VEGF signaling pathway | 7 | 0.007466787 | PLCG2\|PTGS2\|PLA2G4A\|HRAS\|LOC101795759\|RAF1\|PIK3CB |
| FoxO signaling pathway | 11 | 0.008432781 | EGFR\|GABARAPL1\|PCK1\|CCND2\|HRAS\|IGF1R\|LOC101795759\|RAF1\|FOXG1\|PIK3CB\|MAPK8 |
| Glycosaminoglycan degradation | 4 | 0.008647724 | LOC101799286\|SPAM1\|LOC101799098\|HPSE |
| Starch and sucrose metabolism | 5 | 0.011352425 | ENPP3\|LOC101798644\|LOC101794647\|GBE1\|ENPP1 |
| AGE-RAGE signaling pathway in diabetic complications | 8 | 0.026889785 | AGTR1\|PLCG2\|PLCE1\|HRAS\|LOC101795759\|PIK3CB\|EGR1\|MAPK8 |
| Nicotinate and nicotinamide metabolism | 4 | 0.027221787 | NT5M\|ENPP3\|NMNAT2\|ENPP1 |
| Protein processing in endoplasmic reticulum | 11 | 0.02742994 | EDEM3\|MBTPS2\|SEC62\|SSR1\|RNF185\|SEL1L\|LOC101799042\|PRKN\|RRBP1\|MAN1A2\|MAPK8 |
| Cell adhesion molecules (CAMs) | 9 | 0.031400225 | NRCAM\|NRXN3\|CNTN1\|NLGN4X\|CDH2\|CDH4\|CNTNAP2\|ITGA4\|CLDN10 |
| Progesterone-mediated oocyte maturation | 7 | 0.032151934 | CDC16\|PIK3CB\|LOC101795759\|RAF1\|RPS6KA2\|IGF1R\|MAPK8 |
| Pantothenate and CoA biosynthesis | 3 | 0.033849077 | ENPP3\|PPCS\|ENPP1 |
| Adrenergic signaling in cardiomyocytes | 9 | 0.052114876 | AGTR1\|RAPGEF4\|PPP2R3A\|LOC101799806\|LOC101795759\|MYL3\|PIK3CB\|PPP2R2B\|CALM1 |
| Regulation of actin cytoskeleton | 12 | 0.052819343 | EGFR\|LOC101795371\|LIMK2\|CFL2\|WASL\|LOC101795759\|RAF1\|ITGA4\|PIK3CB\|HRAS\|CRKL\|FGFR3 |
| Neuroactive ligand-receptor interaction | 17 | 0.053211603 | ADCYAP1R1\|GLP1R\|LOC101789807\|PTH1R\|GRIA2\|PARD3\|GRID2\|GRM8\|MC4R\|GRIK1\|LOC101796234\|SSTR1\|GRPR\|AGTR1\|CHRNA9\|GRIA4\|GLRB |
| Pentose phosphate pathway | 3 | 0.072248879 | RPIA\|PFKP\|RGN |
| Insulin signaling pathway | 8 | 0.0776064 | PCK1\|HRAS\|LOC101795759\|CALM1\|RAF1\|PIK3CB\|CRKL\|MAPK8 |
| Biosynthesis of unsaturated fatty acids | 3 | 0.085675701 | ELOVL2\|LOC101800182\|SCD |
| SNARE interactions in vesicular transport | 3 | 0.085675701 | STX7\|STX2\|STX1A |
| Ribosome biogenesis in eukaryotes | 5 | 0.094348853 | MDN1\|FCF1\|GNL3\|NVL\|RRP7A |
| Drug metabolism - other enzymes | 3 | 0.100069967 | LOC101791850\|LOC101791461\|LOC101791654 |
| GnRH signaling pathway | 6 | 0.102847025 | EGFR\|PLA2G4A\|HRAS\|CALM1\|RAF1\|MAPK8 |
| PPAR signaling pathway | 5 | 0.108591424 | PCK1\|LOC101798298\|PPARG\|SCD\|ACSL1 |
| Fatty acid metabolism | 4 | 0.117820718 | ELOVL2\|LOC101800182\|SCD\|ACSL1 |
| Sphingolipid metabolism | 4 | 0.130352798 | DEGS1\|DEGS2\|LOC101794817\|SPTLC3 |
| mRNA surveillance pathway | 5 | 0.134479874 | LOC101798009\|PPP2R2B\|PPP2R3A\|SMG7\|ETF1 |
| Tight junction | 8 | 0.134820111 | LOC101795371\|PPP2R2B\|PARD3\|HRAS\|MAP3K20\|CLDN10\|EPB41L2\|YBX3 |
| Pyruvate metabolism | 3 | 0.148274585 | PCK1\|ACYP2\|ACSS2 |
| Carbon metabolism | 6 | 0.158559421 | PFKP\|RPIA\|ACSS2\|GLUD1\|RGN\|SUCLG1 |
| ECM-receptor interaction | 5 | 0.162826675 | RELN\|LAMC2\|LAMA5\|LAMC1\|ITGA4 |
| mTOR signaling pathway | 8 | 0.167544092 | WNT8B\|PIK3CB\|HRAS\|MAPKAP1\|LOC101795759\|RAF1\|RPS6KA2\|IGF1R |
| MAPK signaling pathway | 11 | 0.181228073 | EGFR\|RAF1\|PLA2G4A\|HRAS\|LOC101799806\|MAP3K20\|FGFR3\|RPS6KA2\|CRKL\|MAPK8\|TAB2 |
| Protein export | 2 | 0.187798699 | IMMP2L\|SEC62 |
| Phototransduction | 2 | 0.187798699 | CNGA1\|CALM1 |
| Apoptosis | 7 | 0.200958059 | HRAS\|LOC101799042\|LOC101795759\|RAF1\|PIK3CB\|MAPK8\|LOC101805041 |
| Adipocytokine signaling pathway | 4 | 0.20767205 | PCK1\|SLC2A1\|ACSL1\|MAPK8 |
| Hedgehog signaling pathway | 3 | 0.211729059 | FBXW11\|CCND2\|GLI3 |
| Endocytosis | 12 | 0.217098483 | EGFR\|AMPH\|PARD3\|ERBB4\|GBF1\|WASL\|FGFR3\|IGF1R\|RAB22A\|HRAS\|GRK5\|PDCD6IP |
| Glycosphingolipid biosynthesis - lacto and neolacto series | 2 | 0.227859159 | GCNT2\|B3GALT1 |
| Dorso-ventral axis formation | 2 | 0.227859159 | EGFR\|PIWIL1 |
| Gap junction | 5 | 0.245730149 | EGFR\|LOC101805041\|HRAS\|RAF1\|PRKG2 |
| RNA degradation | 4 | 0.246388347 | EXOSC8\|MPHOSPH6\|PFKP\|HSPA9 |
| Glycosylphosphatidylinositol(GPI)-anchor biosynthesis | 2 | 0.25488241 | PIGZ\|PIGC |
| Fatty acid elongation | 2 | 0.25488241 | ELOVL2\|LOC101800182 |
| Phosphatidylinositol signaling system | 5 | 0.259382047 | PIK3CB\|LOC101795759\|PLCG2\|CALM1\|PLCE1 |
| Citrate cycle (TCA cycle) | 2 | 0.268424387 | PCK1\|SUCLG1 |
| Oocyte meiosis | 5 | 0.273207236 | RPS6KA2\|FBXW11\|IGF1R\|CALM1\|CDC16 |
| Propanoate metabolism | 2 | 0.295475948 | ACSS2\|SUCLG1 |
| Calcium signaling pathway | 8 | 0.298959679 | PPIF\|EGFR\|AGTR1\|PLCG2\|PLCE1\|ERBB4\|GRPR\|CALM1 |
| Ubiquinone and other terpenoid-quinone biosynthesis | 1 | 0.307837473 | COQ2 |
| Glycolysis / Gluconeogenesis | 3 | 0.319543337 | PCK1\|ACSS2\|PFKP |
| Adherens junction | 4 | 0.327460217 | IGF1R\|EGFR\|PARD3\|WASL |
| Vascular smooth muscle contraction | 5 | 0.344129877 | KCNMA1\|RAF1\|AGTR1\|PLA2G4A\|CALM1 |
| Mucin type O-Glycan biosynthesis | 2 | 0.349008051 | GALNT7\|GALNTL6 |
| Ascorbate and aldarate metabolism | 1 | 0.356955673 | RGN |
| Purine metabolism | 7 | 0.357388303 | ENPP3\|POLR2C\|PDE8A\|NT5M\|ENPP1\|ENTPD2\|FHIT |
| Toll-like receptor signaling pathway | 4 | 0.393432835 | PIK3CB\|LOC101795759\|MAPK8\|TAB2 |
| Fatty acid biosynthesis | 1 | 0.402592311 | ACSL1 |
| Nitrogen metabolism | 1 | 0.424182768 | GLUD1 |
| Nucleotide excision repair | 2 | 0.426406141 | RFC3\|ERCC6 |
| Tryptophan metabolism | 2 | 0.438848288 | LOC101797494\|IDO2 |
| Inositol phosphate metabolism | 3 | 0.456666452 | PIK3CB\|PLCG2\|PLCE1 |
| Ether lipid metabolism | 2 | 0.463279342 | LOC101794817\|PLA2G4A |
| Amino sugar and nucleotide sugar metabolism | 2 | 0.475257927 | NPL\|NANP |
| Arginine biosynthesis | 1 | 0.484389435 | GLUD1 |
| Glycosaminoglycan biosynthesis - chondroitin sulfate / dermatan sulfate | 1 | 0.484389435 | UST |
| Salmonella infection | 3 | 0.484690677 | DYNC2H1\|MAPK8\|WASL |
| NOD-like receptor signaling pathway | 2 | 0.487072613 | MAPK8\|TAB2 |
| Melanogenesis | 4 | 0.489942808 | WNT8B\|RAF1\|CALM1\|HRAS |
| Steroid biosynthesis | 1 | 0.50302708 | LOC101797037 |
| One carbon pool by folate | 1 | 0.50302708 | MTHFD1L |
| Wnt signaling pathway | 5 | 0.50724866 | FBXW11\|CCND2\|MMP7\|MAPK8\|WNT8B |
| Arachidonic acid metabolism | 2 | 0.51019559 | PTGS2\|PLA2G4A |
| N-Glycan biosynthesis | 2 | 0.53262357 | MAN1A2\|ALG5 |
| Phagosome | 5 | 0.534195285 | ATP6AP1\|STX7\|LOC101805041\|M6PR\|DYNC2H1 |
| Mismatch repair | 1 | 0.53830799 | RFC3 |
| Peroxisome | 3 | 0.547184257 | ABCD2\|HACL1\|ACSL1 |
| RNA transport | 5 | 0.553957971 | TPR\|NUPL2\|NUP205\|THOC7\|LOC101798009 |
| Spliceosome | 4 | 0.557958416 | ISY1\|LOC101798009\|TCERG1\|PPIH |
| Influenza A | 5 | 0.560453817 | PIK3CB\|LOC101795759\|RAF1\|MAPK8\|IVNS1ABP |
| RNA polymerase | 1 | 0.601541746 | POLR2C |
| Regulation of autophagy | 1 | 0.601541746 | GABARAPL1 |
| Cardiac muscle contraction | 2 | 0.605443102 | LOC101799806\|MYL3 |
| alpha-Linolenic acid metabolism | 1 | 0.615949256 | PLA2G4A |
| Butanoate metabolism | 1 | 0.615949256 | AACS |
| Linoleic acid metabolism | 1 | 0.615949256 | PLA2G4A |
| Biosynthesis of amino acids | 2 | 0.6339093 | PFKP\|RPIA |
| DNA replication | 1 | 0.668560391 | RFC3 |
| p53 signaling pathway | 2 | 0.66932951 | CCND2\|COP1 |
| Ubiquitin mediated proteolysis | 4 | 0.671661277 | FBXW11\|PRKN\|COP1\|CDC16 |
| Glycerophospholipid metabolism | 3 | 0.678707917 | GPAM\|PEMT\|PLA2G4A |
| Fructose and mannose metabolism | 1 | 0.68054733 | PFKP |
| beta-Alanine metabolism | 1 | 0.68054733 | SMS |
| Galactose metabolism | 1 | 0.68054733 | PFKP |
| Pyrimidine metabolism | 3 | 0.68548635 | POLR2C\|CTPS2\|NT5M |
| Retinol metabolism | 1 | 0.692101267 | BCO1 |
| Intestinal immune network for IgA production | 1 | 0.703237823 | ITGA4 |
| Metabolism of xenobiotics by cytochrome P450 | 1 | 0.703237823 | LOC101797494 |
| Proteasome | 1 | 0.713972058 | PSME4 |
| Glycine, serine and threonine metabolism | 1 | 0.713972058 | LOC101794341 |
| Tyrosine metabolism | 1 | 0.72431849 | FAH |
| Alanine, aspartate and glutamate metabolism | 1 | 0.72431849 | GLUD1 |
| Glutathione metabolism | 1 | 0.743903412 | SMS |
| Oxidative phosphorylation | 3 | 0.747430013 | ATP6AP1\|NDUFA5\|NDUFA9 |
| Cysteine and methionine metabolism | 1 | 0.753168395 | SMS |
| Fatty acid degradation | 1 | 0.753168395 | ACSL1 |
| Lysosome | 3 | 0.774541283 | ABCA2\|ATP6AP1\|M6PR |
| Arginine and proline metabolism | 1 | 0.786999157 | SMS |
| Cell cycle | 3 | 0.789632251 | CCND2\|STAG2\|CDC16 |
| Aminoacyl-tRNA biosynthesis | 1 | 0.809296342 | QARS |
| RIG-I-like receptor signaling pathway | 1 | 0.822850275 | MAPK8 |
| Fanconi anemia pathway | 1 | 0.829262031 | FAAP24 |
| Valine, leucine and isoleucine degradation | 1 | 0.829262031 | AACS |
| Herpes simplex infection | 3 | 0.893708527 | PER2\|MAPK8\|TAB2 |
| Glycerolipid metabolism | 1 | 0.898115605 | GPAM |
| TGF-beta signaling pathway | 1 | 0.939222762 | TGIF1 |
| Ribosome | 1 | 0.980661864 | RPL35 |
| Cytokine-cytokine receptor interaction | 2 | 0.983134883 | EGFR\|TNFRSF11B |
