# Supplementary material for: Analysis of whole-genome re-sequencing data of ducks reveals a diverse demographic history and extensive gene flow between Southeast/South Asian and Chinese populations
Source: Genet Sel Evol. 2021 Apr 13;53:35. doi: 10.1186/s12711-021-00627-0 (PMC8042899; doi:10.1186/s12711-021-00627-0)
Supplement: Supplementary file 24 — Additional file 24: Table S9. Prior distribution of the parameters used to generate the eight models in the approximate Bayesian computation (ABC) analysis. [file 12711_2021_627_MOESM24_ESM.docx]

Table S9. Prior distribution of the parameters used to generate the eight models in the approximate Bayesian computation (ABC) analysis

|  | Parameter | Distribution |
| --- | --- | --- |
| Population size | log10_P1 | U[2, 5] |
|  | log10_P2 | U[2, 5] |
|  | log10_P3 | U[2, 5] |
|  | log10_ROOT1 | U[4, 6] |
|  | log10_ROOT2 | U[4, 6] |
| Migration rate | LOGn12 | U[-1.5, 2] |
|  | LOGn21 | U[-1.5, 2] |
|  | LOGn13 | U[-1.5, 2] |
|  | LOGn31 | U[-1.5, 2] |
|  | LOGn23 | U[-1.5, 2] |
|  | LOGn32 | U[-1.5, 2] |
| Split time | T0 | U[50, 5000] |
|  | T1 | U[50, 5000] |
