# Supplementary material for: Analysis of whole-genome re-sequencing data of ducks reveals a diverse demographic history and extensive gene flow between Southeast/South Asian and Chinese populations
Source: Genet Sel Evol. 2021 Apr 13;53:35. doi: 10.1186/s12711-021-00627-0 (PMC8042899; doi:10.1186/s12711-021-00627-0)
Supplement: Supplementary file 25 — Additional file 25: Table S10. Summary statistics for the observed dataset computed by the program arlsumstat. [file 12711_2021_627_MOESM25_ESM.docx]

Table S10. Summary statistics for the observed dataset computed by the program arlsumstat

| Summary statistics | Vaule |
| --- | --- |
| K_1 | 1.99979 |
| K_2 | 2 |
| K_3 | 1.99495 |
| Ksd_1 | 0.0146553 |
| Ksd_2 | 0 |
| Ksd_3 | 0.0708721 |
| mean_K | 1.99825 |
| sd_K | 0.00285436 |
| H_1 | 0.462652 |
| H_2 | 0.484314 |
| H_3 | 0.433891 |
| Hsd_1 | 0.0733155 |
| Hsd_2 | 0.0392123 |
| Hsd_3 | 0.0999579 |
| mean_H | 0.460285 |
| sd_H | 0.0252948 |
| tot_H | 0.497782 |
| S_1 | 9309 |
| S_2 | 9311 |
| S_3 | 9264 |
| prS_1 | 0 |
| prS_2 | 0 |
| prS_3 | 0 |
| mean_S | 9294.67 |
| sd_S | 26.5769 |
| tot_S | 9311 |
| Pi_1 | 4307.75 |
| Pi_2 | 4509.45 |
| Pi_3 | 4039.96 |
| mean_Pi | 4285.72 |
| sd_Pi | 235.52 |
| FST_2_1 | 0.0742741 |
| FST_3_1 | 0.126977 |
| FST_3_2 | 0.102307 |
| PI_2_1 | 4768.58 |
| PI_3_1 | 4779.67 |
| PI_3_2 | 4778.38 |
