# Supplementary material for: Analysis of whole-genome re-sequencing data of ducks reveals a diverse demographic history and extensive gene flow between Southeast/South Asian and Chinese populations
Source: Genet Sel Evol. 2021 Apr 13;53:35. doi: 10.1186/s12711-021-00627-0 (PMC8042899; doi:10.1186/s12711-021-00627-0)
Supplement: Supplementary file 26 — Additional file 26: Table S11. Results of eight models for model choice using nine components obtained by transforming 37 summary statistics with the PLSDA method. [file 12711_2021_627_MOESM26_ESM.docx]

Table S11. Results of eight models for model choice using nine components

|  | model1 | model2 | model3 | model4 | model5 | model6 | model7 | model8 |
| --- | --- | --- | --- | --- | --- | --- | --- | --- |
| BayesFactor | 2.15E-20 | 2.02E-20 | 2.55E-33 | 0.13226 | 0.014935 | 0.011893 | 5.97941 | 3.69E-31 |
| posteriorProbability | 2.15E-20 | 2.02E-20 | 2.55E-33 | 0.116811 | 0.014715 | 0.011753 | 0.856721 | 3.69E-31 |
